# Barcoding Nemo: DNA-Based Identifications for the Ornamental Fish Trade

**DOI:** 10.1371/journal.pone.0006300

**Published:** 2009-07-21

**Authors:** Dirk Steinke, Tyler S. Zemlak, Paul D. N. Hebert

**Affiliations:** Canadian Centre for DNA Barcoding, Biodiversity Institute of Ontario, University of Guelph, Guelph, Ontario, Canada; American Museum of Natural History, United States of America

## Abstract

**Background:**

Trade in ornamental fishes represents, by far, the largest route for the importation of exotic vertebrates. There is growing pressure to regulate this trade with the goal of ensuring that species are sustainably harvested and that their point of origin is accurately reported. One important element of such regulation involves easy access to specimen identifications, a task that is currently difficult for all but specialists because of the large number of species involved. The present study represents an important first step in making identifications more accessible by assembling a DNA barcode reference sequence library for nearly half of the ornamental fish species imported into North America.

**Methodology/Principal Findings:**

Analysis of the cytochrome *c* oxidase subunit I (COI) gene from 391 species from 8 coral reef locations revealed that 98% of these species exhibit distinct barcode clusters, allowing their unambiguous identification. Most species showed little intra-specific variation (adjusted mean = 0.21%), but nine species included two or three lineages showing much more divergence (2.19–6.52%) and likely represent overlooked species complexes. By contrast, three genera contained a species pair or triad that lacked barcode divergence, cases that may reflect hybridization, young taxa or taxonomic over-splitting.

**Conclusions/Significance:**

Although incomplete, this barcode library already provides a new species identification tool for the ornamental fish industry, opening a realm of applications linked to collection practices, regulatory control and conservation.

## Introduction

Over the last 50 years, the international trade in ornamental fishes has grown rapidly. Beginning as a small export fishery in parts of the Indo-Pacific region during the early 20^th^ century, the industry now involves most tropical and subtropical regions, generating some US$200–300 million annually for fishes alone [1]. Target species derive from freshwater and marine environments and include invertebrates (corals, crustaceans, anemones) and vertebrates (fishes) from both natural and captive breeding sources. Most marine fishes derive from wild populations collected from coral reef habitats along the coastal margins of the Atlantic, Pacific and Indian Oceans. Some 800 marine fish species, about 5% of all marine taxa, are involved in this trade with 70% of sales directed to North America [1].

DNA barcoding, the analysis of sequence diversity in a standardized gene region, has gained considerable validation as a tool for species identification and discovery. Several studies have demonstrated its effectiveness for identifying both marine and freshwater fishes [2]–[4], provoking an effort to build a barcode library for all fish species [5]. Currently, records are available for 41771 fishes, representing 6566 fish species on the Barcode of Life Data System, BOLD [6]. DNA barcoding also provides an independent means of testing the validity of existing taxonomic systems, revealing cases of inappropriate synonymy or overlooked taxa. For example, Ward *et al.* [7] and Zemlak *et al.* [8] found several likely cases of overlooked diversity in marine fishes. These results suggest that the species boundaries need to be examined for the heavily exploited populations targeted by the aquarium trade, to properly inform conservation strategies and planning.

The current study has constructed a DNA barcode database for marine fishes that are commonly imported by the pet trade to Canada. This investigation not only provides a further test of the capacity of DNA barcoding to deliver accurate species identifications, but also employs DNA barcodes to highlight potentially cryptic species and discusses some likely impacts of a DNA-based identification system on the ornamental fish trade.

## Materials and Methods

### Taxonomic Coverage

Whenever possible, at least 5 adults were analyzed per species with a total of 1638 individuals, representing 391 species. All specimens are deposited as vouchers in the Biodiversity Institute of Ontario, Guelph, Canada. Collection details are available from the Barcode of Life website (www.barcodinglife.org) in the project file “Aquarium Imports” and are listed in Table S1 by taxonomic rank following Nelson [9]. All samples were wild caught, ‘dead on arrival’ specimens provided by a Canadian importer of marine ornamental fishes. Specimens were frozen immediately and subsequently imaged on a flatbed scanner following a standard protocol [10].

### DNA Analysis

A sample of muscle tissue from each specimen was extracted using an automated Glass Fiber protocol [11]. The 650 bp barcode region of COI was subsequently amplified under the following thermal conditions: 2 min at 95°C; 35 cycles of 0.5 min at 94°C, 0.5 min at 52°C, and 1 min at 72°C; 10 min at 72°C; held at 4°C. The 12.5 µl PCR reaction mixes included 6.25 µl of 10% trehalose, 2.00 µl of ultrapure water, 1.25 µl 10X PCR buffer [200 mM Tris-HCl (pH 8.4), 500 mM KCl], 0.625 µl MgCl2 (50 mM), 0.125 µl of each primer cocktail (0.01 mM, using primer cocktails C_FishF1t1 and C_FishR1t1 from [12], 0.062 µl of each dNTP (10 mM), 0.060 µl of Platinum® Taq Polymerase (Invitrogen), and 2.0 µl of DNA template. PCR amplicons were visualized on a 1.2% agarose gel E-Gel® (Invitrogen) and bidirectionally sequenced using sequencing primers M13F or M13R [12] and the BigDye® Terminator v.3.1 Cycle Sequencing Kit (Applied Biosystems, Inc.) on an ABI 3730 capillary sequencer following manufacturer's instructions.

Sequence data are available on both the Barcode of Life Data System (BOLD, http://www.boldsystems.org, see [6]) and GenBank (Accession numbers in Table S1). Specimen and collection data, sequences, specimen images, and trace files are listed in the same project folder as collection data (Aquarium Imports) on BOLD. A Kimura 2-parameter (K2P) distance metric was employed for sequence comparisons [13]; genetic distances and initial Neighbor-joining (NJ) clustering used the BOLD Management & Analysis System. Confidence in estimated relationships of NJ tree topologies was evaluated by a bootstrap analysis with 1,000 replicates with MEGA version 3.1 [14]. A threshold of 2.0% intra-specific sequence divergence was employed to screen for overlooked species following the recommendation that a sequence divergence value set at 10X the average within species variation (0.21 in this study- see later) is likely to be effective in this regard [15].

## Results

COI amplicons were recovered from all 1638 individuals and there was no evidence of indels or stop codons which might signal the amplification of a NUMT. Sequence length averaged 645 bp (range = 459 to 652 bp), and 98% of the read lengths were greater than 600 bp.

**Figure 1:**
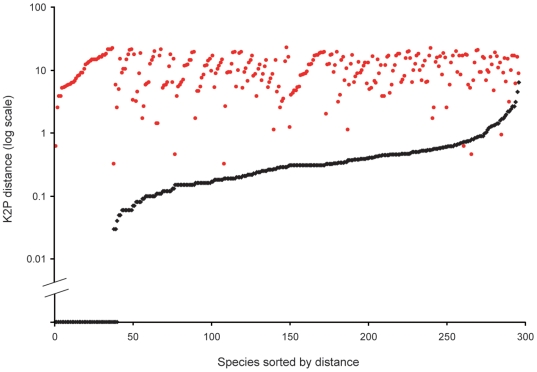
Half-logarithmic dot plot of genetic distances within each species against genetic distances to nearest-neighbor. For each species, there is a black dot showing intraspecific K2P distance and a red dot directly above or below it which shows the distance to its nearest neighbor. Sorting by intra- and interspecific distance allows the relative distances for each species to be seen. This graph indicates that few species have nearest-neighbor distances that are less than the mean intraspecific distance for that species. A line drawn at 1% separates most intraspecific from interspecific values.

A NJ tree of COI sequence divergences (K2P) indicated that most species formed cohesive units with little sequence variation (Figure S1). Mean K2P sequence distance between congeneric species (10.81%) was approximately 26-fold higher than within species variation (0.42%, uncorrected). The clear division between intra- and interspecific sequence variation is further illustrated in the half-logarithmic dot plot displayed which contrasted genetic distances within each species with the distance to its nearest neighbour (Figure 1).

**Figure 2:**
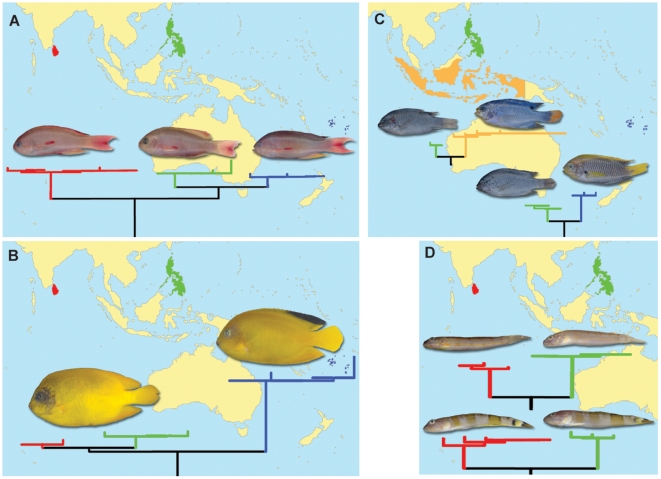
Provisional splits of recognized species with intraspecific distances above 2.0% threshold species with groups associated with spatial differences. (A) Pseudanthias squamipinnis, (B) Centropyge heraldi, (C) Chrysiptera cyanea and Chrysiptera starcki, (D) Valenciennea puellaris and Valenciennea wardii. Branch colors correspond to countries of specimen origin.

Among the 307 species in which two or more specimens were examined, 9 displayed intra-specific divergences greater than 2.0% (Table 1). The mean sequence divergence for these cases averaged 4.46%, with values ranging from 2.19–6.53%. Eight of the nine species formed two distinct clusters, while one (*Pseudanthias squamipinnis*) included three groups. In six of these cases, the lineages were allopatric (Figure 2). Re-analysis of intra-specific divergence values for other samples, after excision of these taxa showing deep divergence, produced an ‘adjusted’ conspecific mean divergence of 0.21%.

**Table 1:** Provisional splits of recognized species with intraspecific distances above the 2.0% threshold. Bootstrap supports for provisional species clusters are shown.

Species	Maximum intraspecific distance	Bootstrap
*Centropyge heraldi*	5.92	87/98
*Chrysiptera cyanea*	2.19	97/98
*Chrysiptera starcki*	2.42	92/99
*Elacatinus evelynae*	3.15	99/99
*Forcipiger flavissimus*	4.86	99/99
*Pseudanthias squamipinnis*	4.06	94/97/99
*Scatophagus argus*	6.52	99/99
*Valenciennea puellaris*	5.13	99/99
*Valenciennea wardii*	5.92	99/99

Sequence divergences between most congeneric taxa were high, averaging 10.81%, but there were exceptions. Three of the thirteen clownfishes (*Amphiprion akallopsisos, A. perideraion* and *A. sandarcinos*) showed sequence sharing as did two species of butterfly fishes (*Chaetodon punctatofasciatus*, *C. multicinctus*) and two species of surgeons (*Zebrasoma flavenscens*, *Z. scopas*). In all of these cases, COI sequences were tightly clustered, differing by less than 0.3% divergence.

## Discussion

More than 98% of the 391 species of ornamental fishes examined in this study possess COI sequences that permit their separation from any other taxon included in this study (or any of the other 6175 fish species on BOLD). This fact reflects the observation that sequence divergence between congeneric taxa was typically high, averaging 10.81%. Conversely, within-species variation for most taxa was very low [adjusted mean = 0.21%], matching the lowest levels of conspecific variation reported in prior barcoding studies on fishes [2], [3], [8], [16]. There were a few exceptions to these general patterns. Nine species showed markedly deeper COI variation, ranging from 2.19–6.52%. Conversely, a few cases were encountered where barcode divergence was either very limited or absent between recognized species. The next sections of the discussion consider these cases in more detail.

### Deep Sequence Divergence within Species

While the 9 species with component lineages showing more than 2% divergence likely represent overlooked species, they might alternatively reflect deep phylogeographic variants linked to female philopatry. While the possibility of sex-biased dispersal has been suspected in a few species of fishes, the idea is still controversial and mainly rests on post-hoc generalizations [17]–[19]. It is unlikely that a blanket explanation of sex-biased dispersal can explain multiple, if any divergences in the present case. We are limited to speculation at the current time because of the complexities involved with the multi-locus frameworks necessary to answer these questions for several taxonomic pairs or triads. We encourage supplemental analysis involving both population genetic and taxonomic contexts. Although this still needs testing, Zink & Barrowclough [20] found that genetic structure at mitochondrial loci was rarely contradicted by nuclear markers. Moreover, there are ‘names- in-waiting’ for some of the taxa in this study.

#### 1. *Centropyge heraldi* Yellow Angelfish

*C. woodheadi*, a very similar species to the yellow angelfish, was described from Fiji [21], but Randall and Carlson [22] synonymized it with *C. heraldi* as no diagnostic morphological characters were apparent. However, the present results support the resurrection of *C. woodheadi* because South Pacific specimens show marked COI divergence from individuals of *C. heraldi* from the Philippine Sea and the Indian Ocean (Figure 2B).

#### 2 & 3. *Chrysiptera spp.* Demoiselles

Two species of *Chrysiptera* showed allopatric divergence and one of these cases may also reflect inappropriate synonymization. *Chrysiptera punctatoperculare* [23] was described from the South China Sea, but Allen [24] synonymized it with *Chrysiptera cyanea*. However, COI divergence between *C. cyanea* from Indonesian and Australian waters (D. Steinke pers. comm.) and those from the Philippines suggest that *C. punctatoperculare* is a valid taxon. Individuals of *C. starcki* from the Philippines and Tonga also differ markedly at COI (Figure 2C), suggesting further overlooked diversity – in this case involving an undescribed demoiselle species.

#### 4. *Pseudanthias squamipinnis* Sea Goldie

Widely distributed in the Indo-Pacific, *Pseudanthias squamipinnis* has a complex taxonomic history being placed, at one time or another, in *Anthias* [25] and *Franzia* [26]. Although all variants of this taxon have now been synonymized as *P. squamipinnis* [27], color pattern differences exist between fishes from different localities [28]. The present barcode results (Figure 2A) suggest that this morphological diversity likely reflects overlooked species, one in the Indian Ocean, a second in the Philippine Sea and a third in the South Pacific.

#### 5 & 6. *Valenciennea species* Gobies

The gobies *Valenciennea puellaris* and *V. wardii* each show more than 5% sequence divergence between lineages from Sri Lankan and Philippine waters (Figure 2D). Cryptic speciation is not uncommon in gobies [29], [30], and DNA barcoding has already helped reveal overlooked species [31], [32]. The present study has likely revealed two more cases.

#### 7–9. Three Cases of Sympatric Divergence

The remaining species (*Forcipiger flavissimus*, *Elacatinus evelynae*, and *Scatophagus argus*) were either collected from a single location, or belong to a group with few barcode records. One specimen of *F. flavissimus* showed 4.86% sequence divergence from the other 7 specimens. This genus contains only two described species and the other taxon (*F. longirostris*) is barcode divergent from both lineages of *F. flavissimus*. *S. argus* from Sri Lanka also showed sympatric divergence with one specimen more than 6% divergent from the other 4 individuals analyzed. Finally, two lineages of the goby *E. evelynae* with more than 3% sequence divergence were collected at the same locality in the Caribbean Sea. Because our collections included few specimens of these taxa, more samples are needed to draw firmer conclusions on species status.

### Cases of Low Inter-specific Variation

Cases where different sympatrically occurring species shared closely similar or identical barcodes were detected in three genera, including three clownfishes (*Amphiprion*) two butterfly fishes (*Chaetodon*) and two surgeons (*Zebrasoma*). Such cases can have three explanations - hybridization, incomplete lineage sorting or over-splitting.

#### 1. *Amphiprion* Clownfishes

The genus *Amphiprion* includes several species with very similar coloration and overlapping variation at otherwise diagnostic morphological characters that make species very difficult to differentiate [33]. The subgenus *Phalerebus* represents a prime example and it includes three species (*A. akallopisos*, *A. periderarion*, *A. sandaracinos*) which show little, if any, barcode divergence. Molecular clock estimates suggest that *A. perideraion* and *A. sandaracinos* diverged from a common ancestor 0.5–1.5 MYA, following an initial separation from *A. akallopisos* 1.1–4.8 MYA [33], providing enough time for reciprocal monophyly at COI, making incomplete lineage sorting an unlikely explanation for their sequence sharing. However, the three species are widely sympatric on reefs in the south Pacific, meaning that hybridization is a possible explanation for sequence sharing.

#### 2. *Chaetodon* Butterflyfishes

*C. multicinctus* and *C. punctatofasciatus* are recognized as a young species pair (∼250,000 years [34]) making incomplete lineage sorting a likely explanation for their barcode sharing.

#### 3. *Zebrasoma*

Sequence sharing by the yellow and brown tang, *Z. flavescens* and *Z. scopas* [35] may similarly be due to incomplete lineage sorting. However, it may also reflect a case of over-splitting because some authors view these taxa as color forms of a single species [36].

### Conclusion

Fast access to biodiversity information is critical. Rising risks of species extinction linked to over-exploitation of natural resources require accurate, up-to-date information to deliver appropriate action. The DNA barcode library constructed in this study provides a basis for reliable species identifications of nearly half of the species exploited by the aquarium industry, opening new ways to manage commercial practices, and providing an independent means of testing existing taxonomic systems. The aquarium trade targets species having a combination of aesthetic appeal, as well as life history attributes that aid survival in captivity. However, the identification of tropical marine fishes using morphological characters is often difficult and usually requires expert consultation. Collectors, wholesalers and retailers, as well as regulatory control agencies will undoubtedly benefit from identification services available from a comprehensive barcoding framework.

Furthermore, present collection methods, which are often destructive to coral reef habitats through direct disturbance by humans or the use of toxic chemicals [37], [38], are evoking substantial concern. Alternative less invasive methods of capture, such as the collection of larval stage fishes with crest nets [39] are, in part, unpopular because juvenile and larval morphology is often distressingly uniform among species, making reliable identifications elusive. The utility of DNA barcodes, regardless of developmental stage [40], [41], provides an attractive means to obtain species identifications and potentially facilitating non-invasive sampling practices.

## Supporting Information

Table S1 This table shows all specimens listed by taxonomic rank following Nelson (1994) with SampleID, BOLD process ID and GenBank Accession No.
(0.63 MB PDF)

Figure S1 A neighbour-joining tree of COI sequence divergences (K2P) in all 1638 individuals of this study. Species names, BOLD process ID, Sample ID, sequence length, and numbers of ambiguous bases are given at branch tips.
(1.59 MB PDF)
