# Supplementary material for: Barcoding Nemo: DNA-Based Identifications for the Ornamental Fish Trade
Source: PLoS One. 2009 Jul 21;4(7):e6300. doi: 10.1371/journal.pone.0006300 (PMC2708913; doi:10.1371/journal.pone.0006300)
Supplement: Table S1 — This table shows all specimens listed by taxonomic rank following Nelson (1994) with SampleID, BOLD process ID and GenBank Accession No. (0.63 MB PDF) [file pone.0006300.s001.pdf]

			Sample#	BOLD #	GenBank Acc#
Class Actinopterygii					
Division Teleostei					
Superorder Acanthopterygii					
Order Beryciformes					
	Family Anomalopidae	*Anomalops katoptron*	HLC-12125	TZAIB014-06	FJ582850
			HLC-12126	TZAIB015-06	FJ582851
			HLC-12127	TZAIB016-06	FJ582852
			HLC-12081	TZAIB158-06	FJ582853
	Family Holocentridae	*Myripristis adusta*	HLC-12134	TZAIB023-06	FJ583669
			HLC-12057	TZAIB134-06	FJ583670
		Myripristis jacobus	HLC-13058	TZAIB435-06	FJ583671
			HLC-13059	TZAIB436-06	FJ583672
			HLC-13060	TZAIB437-06	FJ583673
			HLC-13061	TZAIB438-06	FJ583674
			HLC-15132	TZAIB814-07	FJ583675
		Myripristis murdjan	HLC-13190	TZAIB567-06	FJ583676
		Myripristis violacea	HLC-13191	TZAIB568-06	FJ583677
Order Perciformes					
	Family Acanthuridae	*Acanthurus bahianus*	HLC-12003	TZAIB268-06	FJ582652
			HLC-11999	TZAIB264-06	FJ582653
			HLC-12000	TZAIB265-06	FJ582654
			HLC-12001	TZAIB266-06	FJ582655
			HLC-12002	TZAIB267-06	FJ582656
		Acanthurus chirurgus	HLC-12344	TZAIB327-06	FJ582657
		Acanthurus coeruleus	HLC-11991	TZAIB256-06	FJ582658
			HLC-11987	TZAIB252-06	FJ582659
			HLC-11988	TZAIB253-06	FJ582660
			HLC-11989	TZAIB254-06	FJ582661
			HLC-11990	TZAIB255-06	FJ582662
		Acanthurus japonicus	HLC-11132	TZAIC424-05	FJ582663
			HLC-10936	TZAIC236-05	FJ582664
			HLC-10938	TZAIC238-05	FJ582665
			HLC-10937	TZAIC237-05	FJ582666
		Acanthurus leucosternon	HLC-11784	TZAIC722-06	FJ582667
			HLC-11783	TZAIC721-06	FJ582668
			HLC-11782	TZAIC720-06	FJ582669
			HLC-11120	TZAIC412-05	FJ582670
			HLC-10927	TZAIC227-05	FJ582671
		Acanthurus lineatus	HLC-11871	TZAIC809-06	FJ582672
			HLC-15125	TZAIB807-07	FJ582673
			HLC-15126	TZAIB808-07	FJ582674
			HLC-15127	TZAIB809-07	FJ582675
			HLC-11872	TZAIC810-06	FJ582676
			HLC-11613	TZAIC545-06	FJ582677
			HLC-11612	TZAIC544-06	FJ582678
			HLC-10806	TZAIC106-05	FJ582679
			HLC-10805	TZAIC105-05	FJ582680
		Acanthurus nigricans	HLC-12025	TZAIB102-06	FJ582681
			HLC-12026	TZAIB103-06	FJ582682
			HLC-12027	TZAIB104-06	FJ582683
		Acanthurus nigrofuscus	HLC-13289	TZAIB666-06	FJ582684
			HLC-13290	TZAIB667-06	FJ582685
			HLC-13291	TZAIB668-06	FJ582686
		Acanthurus pyroferus	HLC-13268	TZAIB645-06	FJ582687
			HLC-11651	TZAIC583-06	FJ582688
			HLC-11650	TZAIC582-06	FJ582689
			HLC-10803	TZAIC103-05	FJ582690
		Acanthurus sp.	HLC-11614	TZAIC546-06	FJ582691
		Acanthurus tennentii	HLC-11618	TZAIC550-06	FJ582692
		Acanthurus triostegus	HLC-11967	TZAIB232-06	FJ582693
			HLC-11971	TZAIB236-06	FJ582694
			HLC-11968	TZAIB233-06	FJ582695
			HLC-11969	TZAIB234-06	FJ582696
			HLC-11970	TZAIB235-06	FJ582697
			HLC-10787	TZAIC087-05	FJ582698
			HLC-10788	TZAIC088-05	FJ582699
			HLC-10789	TZAIC089-05	FJ582700
			HLC-11615	TZAIC547-06	FJ582701
		Acanthurus xanthopterus	HLC-11150	TZAIC442-05	FJ582702
		Ctenochaetus striatus	HLC-13214	TZAIB591-06	FJ583302
			HLC-11681	TZAIC613-06	FJ583303
			HLC-11110	TZAIC402-05	FJ583304
		Ctenochaetus strigosus	HLC-12365	TZAIB348-06	FJ583305
			HLC-12366	TZAIB349-06	FJ583306
			HLC-12009	TZAIB274-06	FJ583307
			HLC-12010	TZAIB275-06	FJ583308
			HLC-12011	TZAIB276-06	FJ583309
		Naso lituratus	HLC-11672	TZAIC604-06	FJ583679

		HLC-12376	TZAIB359-06	FJ583680
		HLC-12370	TZAIB353-06	FJ583681
		HLC-12374	TZAIB357-06	FJ583682
		HLC-12375	TZAIB358-06	FJ583683
		HLC-12381	TZAIB364-06	FJ583684
		HLC-12028	TZAIB105-06	FJ583685
		HLC-13090	TZAIB467-06	FJ583686
		HLC-15220	TZAIB890-07	FJ583687
		HLC-15221	TZAIB891-07	FJ583688
		HLC-11937	TZAIB202-06	FJ583689
		HLC-12139	TZAIB028-06	FJ583690
		HLC-10792	TZAIC092-05	FJ583691
		HLC-11597	TZAIC529-06	FJ583692
		HLC-11596	TZAIC528-06	FJ583693
		HLC-10791	TZAIC091-05	FJ583694
	Paracanthurus hepatus	HLC-11810	TZAIC748-06	FJ583801
		HLC-12377	TZAIB360-06	FJ583802
		HLC-12289	TZAIC930-06	FJ583803
		HLC-11809	TZAIC747-06	FJ583804
		HLC-11632	TZAIC564-06	FJ583805
		HLC-11118	TZAIC410-05	FJ583806
		HLC-11115	TZAIC407-05	FJ583807
		HLC-11116	TZAIC408-05	FJ583808
		HLC-11117	TZAIC409-05	FJ583809
	Zebrasoma flavescens	HLC-13068	TZAIB445-06	FJ584263
		HLC-11013	TZAIC305-05	FJ584264
		HLC-11015	TZAIC307-05	FJ584265
		HLC-11017	TZAIC309-05	FJ584266
		HLC-11014	TZAIC306-05	FJ584267
		HLC-11016	TZAIC308-05	FJ584268
	Zebrasoma scopas	HLC-15209	TZAIB879-07	FJ584269
		HLC-11691	TZAIC623-06	FJ584270
		HLC-11573	TZAIC505-06	FJ584271
		HLC-11152	TZAIC444-05	FJ584272
		HLC-11154	TZAIC446-05	FJ584273
		HLC-11153	TZAIC445-05	FJ584274
		HLC-10895	TZAIC195-05	FJ584275
	Zebrasoma veliferum	HLC-11807	TZAIC745-06	FJ584276
		HLC-10804	TZAIC104-05	FJ584277
		HLC-11806	TZAIC744-06	FJ584278
		HLC-11161	TZAIC453-05	FJ584279
		HLC-11162	TZAIC454-05	FJ584280
		HLC-11808	TZAIC746-06	FJ584281
		HLC-11163	TZAIC455-05	FJ584282
Family Apogonidae	*Apogon dovii*	HLC-12347	TZAIB330-06	FJ582860
	Apogon maculatus	HLC-15133	TZAIB815-07	FJ582861
		HLC-15066	TZAIB680-06	FJ582862
	Apogon sealei	HLC-13192	TZAIB569-06	FJ582863
		HLC-13193	TZAIB570-06	FJ582864
		HLC-13195	TZAIB572-06	FJ582865
		HLC-13196	TZAIB573-06	FJ582866
	Apogon townsendi	HLC-15134	TZAIB816-07	FJ582867
	Pterapogon kauderni	HLC-10728	TZAIC028-05	FJ583995
		HLC-10732	TZAIC032-05	FJ583996
		HLC-10729	TZAIC029-05	FJ583997
		HLC-10730	TZAIC030-05	FJ583998
		HLC-10731	TZAIC031-05	FJ583999
	Rhabdamia gracilis	HLC-15141	TZAIB823-07	FJ584049
		HLC-15145	TZAIB827-07	FJ584050
		HLC-15143	TZAIB825-07	FJ584051
		HLC-15144	TZAIB826-07	FJ584052
		HLC-15142	TZAIB824-07	FJ584053
	Sphaeramia nematoptera	HLC-10734	TZAIC034-05	FJ584119
		HLC-10735	TZAIC035-05	FJ584120
		HLC-10737	TZAIC037-05	FJ584121
		HLC-10733	TZAIC033-05	FJ584122
		HLC-10736	TZAIC036-05	FJ584123
Family Blenniidae	*Aspidontus taeniatus*	HLC-11168	TZAIC460-05	FJ582891
	Ecsenius bicolor	HLC-12039	TZAIB116-06	FJ583381
		HLC-12040	TZAIB117-06	FJ583382
		HLC-12208	TZAIC849-06	FJ583383
	Istiblennius edentulus	HLC-11622	TZAIC554-06	FJ583583
	Meiacanthus oualanensis	HLC-11679	TZAIC611-06	FJ583647
		HLC-12311	TZAIB294-06	FJ583648
	Meiacanthus smithi	HLC-13243	TZAIB620-06	FJ583649
		HLC-13131	TZAIB508-06	FJ583650
	Ophioblennius atlanticus	HLC-12088	TZAIB165-06	FJ583745
		HLC-12085	TZAIB162-06	FJ583746
		HLC-12328	TZAIB311-06	FJ583747

		HLC-12329	TZAIB312-06	FJ583748
		HLC-12330	TZAIB313-06	FJ583749
		HLC-12331	TZAIB314-06	FJ583750
		HLC-12332	TZAIB315-06	FJ583751
		HLC-12083	TZAIB160-06	FJ583752
		HLC-12084	TZAIB161-06	FJ583753
		HLC-12086	TZAIB163-06	FJ583754
		HLC-12087	TZAIB164-06	FJ583755
	Salarias fasciatus	HLC-10977	TZAIC277-05	FJ584071
		HLC-10976	TZAIC276-05	FJ584072
		HLC-11736	TZAIC674-06	FJ584073
		HLC-11735	TZAIC673-06	FJ584074
		HLC-11734	TZAIC672-06	FJ584075
		HLC-11733	TZAIC671-06	FJ584076
		HLC-10886	TZAIC186-05	FJ584077
		HLC-10975	TZAIC275-05	FJ584078
		HLC-10974	TZAIC274-05	FJ584079
		HLC-10973	TZAIC273-05	FJ584080
Family Callionymidae	*Dactylopus dactylopus*	HLC-13266	TZAIB643-06	FJ583316
		HLC-13265	TZAIB642-06	FJ583317
		HLC-15158	TZAIB840-07	FJ583318
		HLC-15159	TZAIB841-07	FJ583319
		HLC-15160	TZAIB842-07	FJ583320
	Synchiropus picturatus	HLC-10760	TZAIC060-05	FJ584137
		HLC-10761	TZAIC061-05	FJ584138
		HLC-10759	TZAIC059-05	FJ584139
		HLC-10757	TZAIC057-05	FJ584140
		HLC-10758	TZAIC058-05	FJ584141
	Synchiropus splendidus	HLC-10780	TZAIC080-05	FJ584142
		HLC-10779	TZAIC079-05	FJ584143
		HLC-10778	TZAIC078-05	FJ584144
		HLC-11884	TZAIC822-06	FJ584145
		HLC-11883	TZAIC821-06	FJ584146
		HLC-11882	TZAIC820-06	FJ584147
		HLC-11881	TZAIC819-06	FJ584148
		HLC-11880	TZAIC818-06	FJ584149
		HLC-10777	TZAIC077-05	FJ584150
	Synchiropus stellatus	HLC-11727	TZAIC665-06	FJ584151
		HLC-11176	TZAIC664-06	FJ584152
		HLC-10817	TZAIC117-05	FJ584153
Family Carangidae	*Selene vomer*	HLC-15113	TZAIB796-07	FJ584093
Family Chaenopsidae	*Emblemaria pandionis*	HLC-13082	TZAIB459-06	FJ583394
Family Chaetodontidae	*Chaetodon animaculatus*	HLC-13182	TZAIB559-06	FJ583017
	Chaetodon argentatus	HLC-13185	TZAIB562-06	FJ583018
		HLC-13186	TZAIB563-06	FJ583019
		HLC-13184	TZAIB561-06	FJ583020
	Chaetodon auriga	HLC-13273	TZAIB650-06	FJ583021
		HLC-15060	TZAIB697-06	FJ583022
		HLC-15061	TZAIB698-06	FJ583023
		HLC-15062	TZAIB699-06	FJ583024
		HLC-15063	TZAIB700-06	FJ583025
		HLC-10951	TZAIC251-05	FJ583026
	Chaetodon auripes	HLC-15008	TZAIB701-06	FJ583027
		HLC-15009	TZAIB702-06	FJ583028
		HLC-15011	TZAIB703-06	FJ583029
		HLC-15012	TZAIB704-06	FJ583030
	Chaetodon burgessi	HLC-11790	TZAIC728-06	FJ583031
	Chaetodon capistratus	HLC-12335	TZAIB318-06	FJ583032
		HLC-13282	TZAIB659-06	FJ583033
		HLC-13283	TZAIB660-06	FJ583034
		HLC-13129	TZAIB506-06	FJ583035
		HLC-15131	TZAIB813-07	FJ583036
	Chaetodon collare	HLC-12378	TZAIB361-06	FJ583037
		HLC-12379	TZAIB362-06	FJ583038
		HLC-12380	TZAIB363-06	FJ583039
		HLC-11171	TZAIC659-06	FJ583040
		HLC-11722	TZAIC654-06	FJ583041
		HLC-11785	TZAIC723-06	FJ583042
		HLC-11067	TZAIC359-05	FJ583043
		HLC-11068	TZAIC360-05	FJ583044
	Chaetodon decussatus	HLC-10800	TZAIC100-05	FJ583045
		HLC-12279	TZAIC920-06	FJ583046
		HLC-12278	TZAIC919-06	FJ583047
		HLC-11649	TZAIC581-06	FJ583048
		HLC-11648	TZAIC580-06	FJ583049
		HLC-11646	TZAIC578-06	FJ583050
	Chaetodon ephippium	HLC-10950	TZAIC250-05	FJ583051
		HLC-15002	TZAIB705-06	FJ583052
	Chaetodon flavirostris	HLC-11716	TZAIC648-06	FJ583053

Chaetodon kleinii	HLC-12017	TZAIB282-06	FJ583054
Chaetodon lunula	HLC-15218	TZAIB888-07	FJ583055
	HLC-15219	TZAIB889-07	FJ583056
	HLC-11124	TZAIC416-05	FJ583057
	HLC-10939	TZAIC239-05	FJ583058
	HLC-10941	TZAIC241-05	FJ583059
	HLC-10940	TZAIC240-05	FJ583060
Chaetodon lunulatus	HLC-13032	TZAIB409-06	FJ583061
	HLC-13039	TZAIB416-06	FJ583062
Chaetodon meyeri	HLC-11936	TZAIB201-06	FJ583063
	HLC-11870	TZAIC808-06	FJ583064
	HLC-11654	TZAIC586-06	FJ583065
Chaetodon multicinctus	HLC-12079	TZAIB156-06	FJ583066
	HLC-11111	TZAIC403-05	FJ583067
Chaetodon ocellatus	HLC-13206	TZAIB583-06	FJ583068
Chaetodon ocellicaudus	HLC-15086	TZAIB707-06	FJ583069
Chaetodon ornatissimus	HLC-12346	TZAIB329-06	FJ583070
	HLC-13147	TZAIB524-06	FJ583071
Chaetodon punctatofasciatus	HLC-15169	TZAIB851-07	FJ583072
	HLC-12271	TZAIC912-06	FJ583073
	HLC-11630	TZAIC562-06	FJ583074
Chaetodon quadrimaculatus	HLC-13269	TZAIB646-06	FJ583075
	HLC-13250	TZAIB627-06	FJ583076
Chaetodon rafflesii	HLC-13225	TZAIB602-06	FJ583077
	HLC-13226	TZAIB603-06	FJ583078
	HLC-13227	TZAIB604-06	FJ583079
	HLC-13228	TZAIB605-06	FJ583080
	HLC-13229	TZAIB606-06	FJ583081
	HLC-11652	TZAIC584-06	FJ583082
Chaetodon semeion	HLC-11906	TZAIC844-06	FJ583083
Chaetodon speculum	HLC-13065	TZAIB442-06	FJ583084
Chaetodon striatus	HLC-12326	TZAIB309-06	FJ583085
	HLC-12327	TZAIB310-06	FJ583086
	HLC-13285	TZAIB662-06	FJ583087
	HLC-13286	TZAIB663-06	FJ583088
	HLC-13287	TZAIB664-06	FJ583089
Chaetodon ulietensis	HLC-13197	TZAIB574-06	FJ583101
	HLC-11096	TZAIC388-05	FJ583102
Chaetodon vagabundus	HLC-13270	TZAIB647-06	FJ583103
	HLC-13271	TZAIB648-06	FJ583104
	HLC-11893	TZAIC831-06	FJ583105
	HLC-11779	TZAIC717-06	FJ583106
	HLC-11778	TZAIC716-06	FJ583107
	HLC-11777	TZAIC715-06	FJ583108
	HLC-11647	TZAIC579-06	FJ583109
Chaetodon wiebeli	HLC-13029	TZAIB406-06	FJ583110
Chaetodon xanthurus	HLC-13091	TZAIB468-06	FJ583111
	HLC-13092	TZAIB469-06	FJ583112
	HLC-15001	TZAIB708-06	FJ583113
	HLC-11892	TZAIC830-06	FJ583114
	HLC-11675	TZAIC607-06	FJ583115
	HLC-11674	TZAIC606-06	FJ583116
	HLC-11673	TZAIC605-06	FJ583117
	HLC-10949	TZAIC249-05	FJ583118
Chelmon rostratus	HLC-13042	TZAIB419-06	FJ583126
	HLC-13043	TZAIB420-06	FJ583127
	HLC-13167	TZAIB544-06	FJ583128
	HLC-13168	TZAIB545-06	FJ583129
	HLC-13169	TZAIB546-06	FJ583130
	HLC-11847	TZAIC785-06	FJ583131
	HLC-11845	TZAIC783-06	FJ583132
	HLC-11844	TZAIC782-06	FJ583133
	HLC-11843	TZAIC781-06	FJ583134
Forcipiger flavissimus	HLC-15064	TZAIB709-06	FJ583405
	HLC-15065	TZAIB710-06	FJ583406
	HLC-13164	TZAIB541-06	FJ583407
	HLC-11796	TZAIC734-06	FJ583408
	HLC-11795	TZAIC733-06	FJ583409
	HLC-11565	TZAIC497-06	FJ583410
	HLC-11097	TZAIC389-05	FJ583411
	HLC-11098	TZAIC390-05	FJ583412
Hemitaurichthys polylepis	HLC-12382	TZAIB365-06	FJ583536
	HLC-13146	TZAIB523-06	FJ583537
	HLC-13258	TZAIB635-06	FJ583538
Heniochus acuminatus	HLC-12308	TZAIB291-06	FJ583539
	HLC-15120	TZAIB802-07	FJ583540
	HLC-15121	TZAIB803-07	FJ583541
Heniochus chrysostomus	HLC-11885	TZAIC823-06	FJ583542
	HLC-11791	TZAIC729-06	FJ583543

	Heniochus diphreutes	HLC-11838	TZAIC776-06	FJ583544
		HLC-12309	TZAIB292-06	FJ583545
		HLC-13247	TZAIB624-06	FJ583546
	Heniochus singularius	HLC-13093	TZAIB470-06	FJ583547
	Heniochus varius	HLC-11768	TZAIC706-06	FJ583548
		HLC-11767	TZAIC705-06	FJ583549
Family Cirrhitidae	*Amblycirrhitus pinos*	HLC-12147	TZAIB036-06	FJ582704
		HLC-12148	TZAIB037-06	FJ582705
	Cirrhitichthys falco	HLC-11897	TZAIC835-06	FJ583250
		HLC-15207	TZAIB877-07	FJ583251
		HLC-15097	TZAIB780-07	FJ583252
		HLC-15098	TZAIB781-07	FJ583253
		HLC-11899	TZAIC837-06	FJ583254
		HLC-11898	TZAIC836-06	FJ583255
		HLC-11896	TZAIC834-06	FJ583256
	Cirrhitops fasciatus	HLC-12324	TZAIB307-06	FJ583257
	Neocirrhites armatus	HLC-13126	TZAIB503-06	FJ583715
		HLC-13124	TZAIB501-06	FJ583716
		HLC-13125	TZAIB502-06	FJ583717
		HLC-13128	TZAIB505-06	FJ583718
		HLC-11757	TZAIC695-06	FJ583719
		HLC-11756	TZAIC694-06	FJ583720
		HLC-11755	TZAIC693-06	FJ583721
		HLC-10878	TZAIC178-05	FJ583722
		HLC-10877	TZAIC177-05	FJ583723
	Oxycirrhites typus	HLC-11574	TZAIC506-06	FJ583791
		HLC-10745	TZAIC045-05	FJ583792
		HLC-10744	TZAIC044-05	FJ583793
		HLC-10887	TZAIC187-05	FJ583794
		HLC-10888	TZAIC188-05	FJ583795
	Paracirrhites arcatus	HLC-12066	TZAIB143-06	FJ583821
		HLC-12067	TZAIB144-06	FJ583822
		HLC-12068	TZAIB145-06	FJ583823
		HLC-12069	TZAIB146-06	FJ583824
		HLC-12070	TZAIB147-06	FJ583825
		HLC-15197	TZAIB867-07	FJ583826
Family Echeneidae	*Echeneis naucrates*	HLC-13267	TZAIB644-06	FJ583378
Family Ephippidae	*Chaetodipterus faber*	HLC-13080	TZAIB457-06	FJ583016
	Platax orbicularis	HLC-12071	TZAIB148-06	FJ583852
		HLC-13157	TZAIB534-06	FJ583853
		HLC-13158	TZAIB535-06	FJ583854
		HLC-13159	TZAIB536-06	FJ583855
		HLC-13160	TZAIB537-06	FJ583856
	Platax pinnatus	HLC-15122	TZAIB804-07	FJ583857
		HLC-11005	TZAIC297-05	FJ583858
	Platax teira	HLC-13161	TZAIB538-06	FJ583859
Family Gobiidae	*Amblyeleotris guttata*	HLC-13260	TZAIB637-06	FJ582706
		HLC-13261	TZAIB638-06	FJ582707
		HLC-13262	TZAIB639-06	FJ582708
		HLC-13263	TZAIB640-06	FJ582709
		HLC-13264	TZAIB641-06	FJ582710
	Amblyeleotris steinitzi	HLC-13176	TZAIB553-06	FJ582711
		HLC-13178	TZAIB555-06	FJ582712
	Amblyeleotris sungami	HLC-12043	TZAIB120-06	FJ582713
		HLC-12044	TZAIB121-06	FJ582714
		HLC-12045	TZAIB122-06	FJ582715
		HLC-12046	TZAIB123-06	FJ582716
	Amblyeleotris wheeleri	HLC-12301	TZAIB284-06	FJ582717
		HLC-13204	TZAIB581-06	FJ582718
	Amblygobius decussatus	HLC-13096	TZAIB473-06	FJ582719
		HLC-13198	TZAIB575-06	FJ582720
		HLC-13199	TZAIB576-06	FJ582721
		HLC-11158	TZAIC450-05	FJ582722
		HLC-10978	TZAIC278-05	FJ582723
	Amblygobius phalaena	HLC-13170	TZAIB547-06	FJ582724
		HLC-13171	TZAIB548-06	FJ582725
	Cryptocentrus cinctus	HLC-10979	TZAIC279-05	FJ583280
		HLC-11543	TZAIC475-06	FJ583281
		HLC-11542	TZAIC474-06	FJ583282
		HLC-11540	TZAIC472-06	FJ583283
		HLC-10890	TZAIC190-05	FJ583284
		HLC-10892	TZAIC192-05	FJ583285
	Cryptocentrus cyanotaenia	HLC-12191	TZAIB080-06	FJ583286
	Cryptocentrus leptocephalus	HLC-11900	TZAIC838-06	FJ583287
		HLC-11903	TZAIC841-06	FJ583288
		HLC-11901	TZAIC839-06	FJ583289
		HLC-12262	TZAIC903-06	FJ583290
		HLC-12261	TZAIC902-06	FJ583291
		HLC-12260	TZAIC901-06	FJ583292

	HLC-12259	TZAIC900-06	FJ583293
	HLC-12258	TZAIC899-06	FJ583294
	HLC-11902	TZAIC840-06	FJ583295
	HLC-10980	TZAIC280-05	FJ583296
Cryptocentrus pavoninoides	HLC-12277	TZAIC918-06	FJ583297
	HLC-12276	TZAIC917-06	FJ583298
	HLC-12275	TZAIC916-06	FJ583299
	HLC-12274	TZAIC915-06	FJ583300
	HLC-12273	TZAIC914-06	FJ583301
Ctenogobiops tangaroai	HLC-15227	TZAIB897-07	FJ583310
Elacatinus evelynae	HLC-10781	TZAIC081-05	FJ583384
	HLC-15083	TZAIB713-06	FJ583385
	HLC-15084	TZAIB714-06	FJ583386
	HLC-15114	TZAIB797-07	FJ583387
	HLC-15115	TZAIB798-07	FJ583388
Elacatinus oceanops	HLC-13153	TZAIB530-06	FJ583389
	HLC-13154	TZAIB531-06	FJ583390
	HLC-13155	TZAIB532-06	FJ583391
	HLC-15006	TZAIB711-06	FJ583392
	HLC-15007	TZAIB712-06	FJ583393
Fusigobius inframaculatus	HLC-11774	TZAIC712-06	FJ583413
Fusigobius signipinnis	HLC-11556	TZAIC488-06	FJ583414
Gobiodon ceramensis	HLC-15078	TZAIB673-06	FJ583428
	HLC-15079	TZAIB674-06	FJ583429
	HLC-15080	TZAIB675-06	FJ583430
	HLC-15081	TZAIB676-06	FJ583431
	HLC-15082	TZAIB677-06	FJ583432
Gobiodon histrio	HLC-10784	TZAIC084-05	FJ583433
	HLC-10783	TZAIC083-05	FJ583434
	HLC-11830	TZAIC768-06	FJ583435
	HLC-15044	TZAIB715-06	FJ583436
	HLC-15045	TZAIB716-06	FJ583437
	HLC-15046	TZAIB717-06	FJ583438
	HLC-15047	TZAIB718-06	FJ583439
	HLC-15048	TZAIB719-06	FJ583440
	HLC-12157	TZAIB046-06	FJ583441
	HLC-12158	TZAIB047-06	FJ583442
	HLC-12159	TZAIB048-06	FJ583443
	HLC-12160	TZAIB049-06	FJ583444
	HLC-12161	TZAIB050-06	FJ583445
	HLC-12162	TZAIB051-06	FJ583446
	HLC-11829	TZAIC767-06	FJ583447
	HLC-10786	TZAIC086-05	FJ583448
	HLC-10785	TZAIC085-05	FJ583449
	HLC-10782	TZAIC082-05	FJ583450
Gobiodon okinawae	HLC-13274	TZAIB651-06	FJ583451
	HLC-13275	TZAIB652-06	FJ583452
	HLC-13276	TZAIB653-06	FJ583453
	HLC-13278	TZAIB655-06	FJ583454
	HLC-10891	TZAIC191-05	FJ583455
	HLC-10893	TZAIC193-05	FJ583456
Gobiodon quinquestrigatus	HLC-10896	TZAIC196-05	FJ583457
	HLC-10898	TZAIC198-05	FJ583458
	HLC-10897	TZAIC197-05	FJ583459
Paragobiodon lacunicolus	HLC-15194	TZAIB864-07	FJ583828
	HLC-15195	TZAIB865-07	FJ583829
Taenioides sp.	HLC-11740	TZAIC678-06	FJ584163
	HLC-11739	TZAIC677-06	FJ584164
	HLC-11738	TZAIC676-06	FJ584165
	HLC-11737	TZAIC675-06	FJ584166
	HLC-10881	TZAIC181-05	FJ584167
Valenciennea helsdingenii	HLC-11963	TZAIB228-06	FJ584196
	HLC-11852	TZAIC790-06	FJ584197
	HLC-11851	TZAIC789-06	FJ584198
	HLC-11850	TZAIC788-06	FJ584199
	HLC-11849	TZAIC787-06	FJ584200
	HLC-11848	TZAIC786-06	FJ584201
Valenciennea longipinnis	HLC-12266	TZAIC907-06	FJ584202
Valenciennea muralis	HLC-12267	TZAIC908-06	FJ584203
	HLC-12265	TZAIC906-06	FJ584204
	HLC-11019	TZAIC311-05	FJ584205
Valenciennea puellaris	HLC-10815	TZAIC115-05	FJ584206
	HLC-10814	TZAIC114-05	FJ584207
	HLC-12252	TZAIC893-06	FJ584208
	HLC-12251	TZAIC892-06	FJ584209
	HLC-12250	TZAIC891-06	FJ584210
	HLC-12249	TZAIC890-06	FJ584211
	HLC-12248	TZAIC889-06	FJ584212
	HLC-11578	TZAIC510-06	FJ584213

	Valenciennea sexguttata	HLC-12052	TZAIB129-06	FJ584214
		HLC-12051	TZAIB128-06	FJ584215
		HLC-12050	TZAIB127-06	FJ584216
		HLC-12253	TZAIC894-06	FJ584217
		HLC-12255	TZAIC896-06	FJ584218
		HLC-12103	TZAIB180-06	FJ584219
		HLC-12257	TZAIC898-06	FJ584220
		HLC-12256	TZAIC897-06	FJ584221
		HLC-12254	TZAIC895-06	FJ584222
		HLC-11038	TZAIC330-05	FJ584223
		HLC-11037	TZAIC329-05	FJ584224
	Valenciennea strigata	HLC-15003	TZAIB720-06	FJ584225
		HLC-15099	TZAIB782-07	FJ584226
		HLC-11011	TZAIC303-05	FJ584227
		HLC-11010	TZAIC302-05	FJ584228
		HLC-11009	TZAIC301-05	FJ584229
		HLC-11008	TZAIC300-05	FJ584230
		HLC-11012	TZAIC304-05	FJ584231
	Valenciennea wardii	HLC-12163	TZAIB052-06	FJ584232
		HLC-12056	TZAIB133-06	FJ584233
		HLC-12102	TZAIB179-06	FJ584234
		HLC-12104	TZAIB181-06	FJ584235
		HLC-12105	TZAIB182-06	FJ584236
		HLC-15111	TZAIB794-07	FJ584237
		HLC-10813	TZAIC113-05	FJ584238
		HLC-10811	TZAIC111-05	FJ584239
		HLC-10812	TZAIC112-05	FJ584240
Family Grammatidae	*Gramma loreto*	HLC-11984	TZAIB249-06	FJ583467
		HLC-11983	TZAIB248-06	FJ583468
		HLC-11986	TZAIB251-06	FJ583469
		HLC-11982	TZAIB247-06	FJ583470
		HLC-10998	TZAIC290-05	FJ583471
		HLC-11000	TZAIC292-05	FJ583472
		HLC-11001	TZAIC293-05	FJ583473
		HLC-11004	TZAIC296-05	FJ583474
		HLC-10999	TZAIC291-05	FJ583475
		HLC-11002	TZAIC294-05	FJ583476
		HLC-11003	TZAIC295-05	FJ583477
	Gramma melacara	HLC-12359	TZAIB342-06	FJ583478
		HLC-12362	TZAIB345-06	FJ583479
		HLC-12080	TZAIB157-06	FJ583480
		HLC-11985	TZAIB250-06	FJ583481
		HLC-11952	TZAIB217-06	FJ583482
		HLC-11953	TZAIB218-06	FJ583483
Family Haemulidae	*Anisotremus virginicus*	HLC-13035	TZAIB412-06	FJ582849
	Haemulon plumierii	HLC-12048	TZAIB125-06	FJ583484
	Plectorhinchus chaetodonoides	HLC-11165	TZAIC457-05	FJ583860
		HLC-11164	TZAIC456-05	FJ583861
		HLC-11166	TZAIC458-05	FJ583862
		HLC-11006	TZAIC298-05	FJ583863
		HLC-11007	TZAIC299-05	FJ583864
	Plectorhinchus picus	HLC-12113	TZAIB002-06	FJ583865
		HLC-12112	TZAIB001-06	FJ583866
		HLC-12114	TZAIB003-06	FJ583867
		HLC-12115	TZAIB004-06	FJ583868
Family Labridae	*Anampses lineatus*	HLC-10808	TZAIC108-05	FJ582831
	Anampses meleagrides	HLC-13239	TZAIB616-06	FJ582832
		HLC-13240	TZAIB617-06	FJ582833
		HLC-13245	TZAIB622-06	FJ582834
		HLC-13246	TZAIB623-06	FJ582835
		HLC-12322	TZAIB305-06	FJ582836
		HLC-15049	TZAIB724-06	FJ582837
		HLC-15050	TZAIB725-06	FJ582838
		HLC-15051	TZAIB726-06	FJ582839
		HLC-15052	TZAIB727-06	FJ582840
		HLC-15053	TZAIB728-06	FJ582841
		HLC-13244	TZAIB621-06	FJ582842
	Anampses neoguinaicus	HLC-13183	TZAIB560-06	FJ582843
		HLC-11062	TZAIC354-05	FJ582844
		HLC-11705	TZAIC637-06	FJ582845
		HLC-11800	TZAIC738-06	FJ582846
		HLC-11560	TZAIC492-06	FJ582847
	Anampses twistii	HLC-11028	TZAIC320-05	FJ582848
	Bodianus anthioides	HLC-13187	TZAIB564-06	FJ582899
	Bodianus loxozonus	HLC-11677	TZAIC609-06	FJ582900
		HLC-11577	TZAIC509-06	FJ582901
	Bodianus pulchellus	HLC-11025	TZAIC317-05	FJ582902
	Bodianus rufus	HLC-12307	TZAIB290-06	FJ582903
		HLC-15089	TZAIB772-07	FJ582904

	HLC-15090	TZAIB773-07	FJ582905
	HLC-15091	TZAIB774-07	FJ582906
	HLC-15092	TZAIB775-07	FJ582907
Cheilinus fasciatus	HLC-13075	TZAIB452-06	FJ583119
	HLC-11559	TZAIC491-06	FJ583120
Cheilinus oxycephalus	HLC-11085	TZAIC377-05	FJ583121
	HLC-12142	TZAIB031-06	FJ583122
	HLC-12143	TZAIB032-06	FJ583123
	HLC-12144	TZAIB033-06	FJ583124
	HLC-12145	TZAIB034-06	FJ583125
Cirrhilabrus cyanopleura	HLC-15146	TZAIB828-07	FJ583213
	HLC-15148	TZAIB830-07	FJ583214
	HLC-15149	TZAIB831-07	FJ583215
	HLC-11669	TZAIC601-06	FJ583216
	HLC-11668	TZAIC600-06	FJ583217
	HLC-11667	TZAIC599-06	FJ583218
	HLC-11666	TZAIC598-06	FJ583219
	HLC-11665	TZAIC597-06	FJ583220
Cirrhilabrus exquisitus	HLC-11551	TZAIC483-06	FJ583221
	HLC-11550	TZAIC482-06	FJ583222
Cirrhilabrus flavidorsalis	HLC-11964	TZAIB229-06	FJ583223
	HLC-11965	TZAIB230-06	FJ583224
	HLC-11966	TZAIB231-06	FJ583225
	HLC-15100	TZAIB783-07	FJ583226
	HLC-15101	TZAIB784-07	FJ583227
	HLC-11997	TZAIB262-06	FJ583228
	HLC-11998	TZAIB263-06	FJ583229
	HLC-11664	TZAIC596-06	FJ583230
	HLC-11663	TZAIC595-06	FJ583231
	HLC-11662	TZAIC594-06	FJ583232
Cirrhilabrus lubbocki	HLC-11836	TZAIC774-06	FJ583233
	HLC-11837	TZAIC775-06	FJ583234
	HLC-11592	TZAIC524-06	FJ583235
	HLC-11591	TZAIC523-06	FJ583236
	HLC-10807	TZAIC107-05	FJ583237
	HLC-10810	TZAIC110-05	FJ583238
Cirrhilabrus rubrimarginatus	HLC-13256	TZAIB633-06	FJ583239
	HLC-10964	TZAIC264-05	FJ583240
Cirrhilabrus rubriventralis	HLC-11972	TZAIB237-06	FJ583241
	HLC-10963	TZAIC263-05	FJ583242
Cirrhilabrus scottorum	HLC-11797	TZAIC735-06	FJ583243
	HLC-11701	TZAIC633-06	FJ583244
	HLC-11799	TZAIC737-06	FJ583245
	HLC-11798	TZAIC736-06	FJ583246
	HLC-11566	TZAIC498-06	FJ583247
	HLC-11136	TZAIC428-05	FJ583248
	HLC-10972	TZAIC272-05	FJ583249
Coris formosa	HLC-15208	TZAIB878-07	FJ583258
	HLC-11891	TZAIC829-06	FJ583259
	HLC-11776	TZAIC714-06	FJ583260
Coris gaimard	HLC-12072	TZAIB149-06	FJ583261
	HLC-12073	TZAIB150-06	FJ583262
	HLC-11175	TZAIC663-06	FJ583263
	HLC-11172	TZAIC660-06	FJ583264
	HLC-11087	TZAIC379-05	FJ583265
	HLC-11086	TZAIC378-05	FJ583266
	HLC-10933	TZAIC233-05	FJ583267
	HLC-10934	TZAIC234-05	FJ583268
	HLC-10935	TZAIC235-05	FJ583269
	HLC-11070	TZAIC362-05	FJ583270
Gomphosus varius	HLC-11781	TZAIC719-06	FJ583460
	HLC-11815	TZAIC753-06	FJ583461
	HLC-15205	TZAIB875-07	FJ583462
	HLC-15236	TZAIB906-07	FJ583463
	HLC-13079	TZAIB456-06	FJ583464
	HLC-11104	TZAIC396-05	FJ583465
	HLC-11103	TZAIC395-05	FJ583466
Halichoeres biocellatus	HLC-11714	TZAIC646-06	FJ583485
	HLC-11174	TZAIC662-06	FJ583486
	HLC-11713	TZAIC645-06	FJ583487
	HLC-11712	TZAIC644-06	FJ583488
	HLC-11167	TZAIC459-05	FJ583489
Halichoeres chloropterus	HLC-11023	TZAIC315-05	FJ583490
	HLC-11772	TZAIC710-06	FJ583491
	HLC-11771	TZAIC709-06	FJ583492
	HLC-11770	TZAIC708-06	FJ583493
	HLC-11022	TZAIC314-05	FJ583494
Halichoeres chrysus	HLC-11587	TZAIC519-06	FJ583495
	HLC-11588	TZAIC520-06	FJ583496

	HLC-11586	TZAIC518-06	FJ583497
	HLC-11589	TZAIC521-06	FJ583498
	HLC-10809	TZAIC109-05	FJ583499
Halichoeres garnoti	HLC-12352	TZAIB335-06	FJ583500
	HLC-12353	TZAIB336-06	FJ583501
Halichoeres hortulanus	HLC-12155	TZAIB044-06	FJ583502
	HLC-12156	TZAIB045-06	FJ583503
	HLC-10819	TZAIC119-05	FJ583504
	HLC-10818	TZAIC118-05	FJ583505
Halichoeres leucoxanthus	HLC-12064	TZAIB141-06	FJ583506
	HLC-12061	TZAIB138-06	FJ583507
	HLC-12062	TZAIB139-06	FJ583508
	HLC-12063	TZAIB140-06	FJ583509
	HLC-12065	TZAIB142-06	FJ583510
Halichoeres marginatus	HLC-11595	TZAIC527-06	FJ583511
	HLC-10849	TZAIC149-05	FJ583512
	HLC-10966	TZAIC266-05	FJ583513
Halichoeres melanurus	HLC-10848	TZAIC148-05	FJ583514
	HLC-11890	TZAIC828-06	FJ583515
	HLC-11889	TZAIC827-06	FJ583516
	HLC-11888	TZAIC826-06	FJ583517
	HLC-11887	TZAIC825-06	FJ583518
	HLC-11886	TZAIC824-06	FJ583519
	HLC-10847	TZAIC147-05	FJ583520
	HLC-10968	TZAIC268-05	FJ583521
	HLC-10965	TZAIC265-05	FJ583522
	HLC-10967	TZAIC267-05	FJ583523
	HLC-10969	TZAIC269-05	FJ583524
Halichoeres ornatissimus	HLC-13106	TZAIB483-06	FJ583525
	HLC-13108	TZAIB485-06	FJ583526
	HLC-11709	TZAIC641-06	FJ583527
	HLC-11708	TZAIC640-06	FJ583528
	HLC-11792	TZAIC730-06	FJ583529
	HLC-13107	TZAIB484-06	FJ583530
	HLC-10885	TZAIC185-05	FJ583531
Halichoeres sp.	HLC-11585	TZAIC517-06	FJ583532
Hemigymnus fasciatus	HLC-13076	TZAIB453-06	FJ583533
	HLC-13077	TZAIB454-06	FJ583534
	HLC-13078	TZAIB455-06	FJ583535
Labrichthys unilineatus	HLC-15228	TZAIB898-07	FJ583587
Labroides dimidiatus	HLC-10841	TZAIC141-05	FJ583588
	HLC-10840	TZAIC140-05	FJ583589
	HLC-12211	TZAIC852-06	FJ583590
	HLC-12210	TZAIC851-06	FJ583591
	HLC-11605	TZAIC537-06	FJ583592
	HLC-11604	TZAIC536-06	FJ583593
	HLC-11603	TZAIC535-06	FJ583594
	HLC-10952	TZAIC252-05	FJ583595
	HLC-10959	TZAIC259-05	FJ583596
	HLC-10956	TZAIC256-05	FJ583597
	HLC-10954	TZAIC254-05	FJ583598
	HLC-10958	TZAIC258-05	FJ583599
	HLC-10955	TZAIC255-05	FJ583600
	HLC-10953	TZAIC253-05	FJ583601
	HLC-10957	TZAIC257-05	FJ583602
	HLC-10961	TZAIC261-05	FJ583603
	HLC-10960	TZAIC260-05	FJ583604
Macropharyngodon meleagris	HLC-15192	TZAIB862-07	FJ583630
	HLC-15059	TZAIB729-06	FJ583631
	HLC-11700	TZAIC632-06	FJ583632
	HLC-11699	TZAIC631-06	FJ583633
	HLC-11698	TZAIC630-06	FJ583634
	HLC-11697	TZAIC629-06	FJ583635
	HLC-11696	TZAIC628-06	FJ583636
	HLC-11841	TZAIC779-06	FJ583637
	HLC-11840	TZAIC778-06	FJ583638
	HLC-11539	TZAIC471-06	FJ583639
	HLC-11099	TZAIC391-05	FJ583640
	HLC-11100	TZAIC392-05	FJ583641
	HLC-11101	TZAIC393-05	FJ583642
	HLC-11102	TZAIC394-05	FJ583643
	HLC-10971	TZAIC271-05	FJ583644
	HLC-10970	TZAIC270-05	FJ583645
Macropharyngodon ornatus	HLC-11063	TZAIC355-05	FJ583646
Novaculichthys taeniourus	HLC-12323	TZAIB306-06	FJ583739
	HLC-15157	TZAIB839-07	FJ583740
	HLC-12228	TZAIC869-06	FJ583741
	HLC-12227	TZAIC868-06	FJ583742
	HLC-11113	TZAIC405-05	FJ583743

	Paracheilinus carpenteri	HLC-12246	TZAIC887-06	FJ583810
		HLC-12243	TZAIC884-06	FJ583811
		HLC-12247	TZAIC888-06	FJ583812
		HLC-12245	TZAIC886-06	FJ583813
		HLC-12244	TZAIC885-06	FJ583814
		HLC-11024	TZAIC316-05	FJ583815
	Paracheilinus mccoskeri	HLC-15136	TZAIB818-07	FJ583816
		HLC-15137	TZAIB819-07	FJ583817
		HLC-15138	TZAIB820-07	FJ583818
		HLC-15139	TZAIB821-07	FJ583819
		HLC-15140	TZAIB822-07	FJ583820
	Pseudocheilinus evanidus	HLC-13123	TZAIB500-06	FJ583964
		HLC-15166	TZAIB848-07	FJ583965
		HLC-12341	TZAIB324-06	FJ583966
	Pseudocheilinus hexataenia	HLC-15020	TZAIB721-06	FJ583967
		HLC-15021	TZAIB722-06	FJ583968
		HLC-15022	TZAIB723-06	FJ583969
		HLC-11671	TZAIC603-06	FJ583970
		HLC-11670	TZAIC602-06	FJ583971
	Pseudocheilinus octotaenia	HLC-15118	TZAIB800-07	FJ583972
	Pseudocheilinus tetrataenia	HLC-15102	TZAIB785-07	FJ583973
		HLC-15103	TZAIB786-07	FJ583974
		HLC-15104	TZAIB787-07	FJ583975
		HLC-15105	TZAIB788-07	FJ583976
		HLC-15106	TZAIB789-07	FJ583977
	Pseudocoris yamashiroi	HLC-11558	TZAIC490-06	FJ583991
		HLC-11557	TZAIC489-06	FJ583992
	Pseudodax moluccanus	HLC-12054	TZAIB131-06	FJ583993
		HLC-12055	TZAIB132-06	FJ583994
	Stethojulis bandanensis	HLC-15196	TZAIB866-07	FJ584126
		HLC-15199	TZAIB869-07	FJ584127
	Thalassoma bifasciatum	HLC-13120	TZAIB497-06	FJ584172
		HLC-12336	TZAIB319-06	FJ584173
		HLC-12077	TZAIB154-06	FJ584174
		HLC-12074	TZAIB151-06	FJ584175
		HLC-12075	TZAIB152-06	FJ584176
		HLC-12076	TZAIB153-06	FJ584177
		HLC-12078	TZAIB155-06	FJ584178
		HLC-13151	TZAIB528-06	FJ584179
		HLC-13173	TZAIB550-06	FJ584180
	Thalassoma hardwicke	HLC-15201	TZAIB871-07	FJ584181
		HLC-15203	TZAIB873-07	FJ584182
	Thalassoma lunare	HLC-13072	TZAIB449-06	FJ584183
		HLC-13073	TZAIB450-06	FJ584184
		HLC-15198	TZAIB868-07	FJ584185
	Thalassoma lutescens	HLC-11879	TZAIC817-06	FJ584186
		HLC-11805	TZAIC743-06	FJ584187
		HLC-11804	TZAIC742-06	FJ584188
		HLC-11803	TZAIC741-06	FJ584189
		HLC-11802	TZAIC740-06	FJ584190
		HLC-11801	TZAIC739-06	FJ584191
	Thalassoma quinquevittatum	HLC-13097	TZAIB474-06	FJ584192
		HLC-13098	TZAIB475-06	FJ584193
		HLC-13099	TZAIB476-06	FJ584194
		HLC-13100	TZAIB477-06	FJ584195
Family Labrisomidae	*Paraclinus marmoratus*	HLC-11962	TZAIB227-06	FJ583827
Family Lutjanidae	*Macolor niger*	HLC-11905	TZAIC843-06	FJ583628
		HLC-11904	TZAIC842-06	FJ583629
	Symphorichthys spilurus	HLC-TSZ-013	TZAIC013-05	FJ584133
		HLC-15004	TZAIB730-06	FJ584134
		HLC-13166	TZAIB543-06	FJ584135
Family Malacanthidae	*Hoplolatilus chlupatyi*	HLC-13174	TZAIB551-06	FJ583569
		HLC-15167	TZAIB849-07	FJ583570
	Hoplolatilus marcosi	HLC-13140	TZAIB517-06	FJ583571
		HLC-13141	TZAIB518-06	FJ583572
		HLC-13142	TZAIB519-06	FJ583573
		HLC-15096	TZAIB779-07	FJ583574
	Hoplolatilus purpureus	HLC-11764	TZAIC702-06	FJ583575
		HLC-11119	TZAIC411-05	FJ583576
Family Microdesmidae	*Nemateleotris decora*	HLC-11629	TZAIC561-06	FJ583701
		HLC-11628	TZAIC560-06	FJ583702
		HLC-11627	TZAIC559-06	FJ583703
		HLC-11057	TZAIC349-05	FJ583704
	Nemateleotris magnifica	HLC-11091	TZAIC383-05	FJ583705
		HLC-13084	TZAIB461-06	FJ583706
		HLC-13085	TZAIB462-06	FJ583707
		HLC-13086	TZAIB463-06	FJ583708
		HLC-13087	TZAIB464-06	FJ583709
		HLC-13088	TZAIB465-06	FJ583710

		HLC-11090	TZAIC382-05	FJ583711
		HLC-11092	TZAIC384-05	FJ583712
		HLC-11089	TZAIC381-05	FJ583713
		HLC-11088	TZAIC380-05	FJ583714
	Ptereleotris evides	HLC-12128	TZAIB017-06	FJ584000
		HLC-12129	TZAIB018-06	FJ584001
	Ptereleotris monoptera	HLC-12218	TZAIC859-06	FJ584002
		HLC-12220	TZAIC861-06	FJ584003
		HLC-12217	TZAIC858-06	FJ584004
		HLC-11659	TZAIC591-06	FJ584005
		HLC-11658	TZAIC590-06	FJ584006
		HLC-11657	TZAIC589-06	FJ584007
		HLC-11656	TZAIC588-06	FJ584008
		HLC-11655	TZAIC587-06	FJ584009
	Ptereleotris sp.	HLC-12269	TZAIC910-06	FJ584010
	Ptereleotris zebra	HLC-11950	TZAIB215-06	FJ584011
		HLC-11948	TZAIB213-06	FJ584012
		HLC-11947	TZAIB212-06	FJ584013
		HLC-11946	TZAIB211-06	FJ584014
		HLC-11949	TZAIB214-06	FJ584015
Family Monodactylidae	*Monodactylus argenteus*	HLC-10993	TZAIC285-05	FJ583663
		HLC-10994	TZAIC286-05	FJ583664
		HLC-10995	TZAIC287-05	FJ583665
		HLC-10996	TZAIC288-05	FJ583666
		HLC-10997	TZAIC289-05	FJ583667
Family Mullidae	*Parupeneus barberinoides*	HLC-15029	TZAIB731-06	FJ583832
		HLC-15030	TZAIB732-06	FJ583833
		HLC-15031	TZAIB733-06	FJ583834
		HLC-15032	TZAIB734-06	FJ583835
		HLC-11031	TZAIC323-05	FJ583836
		HLC-11032	TZAIC324-05	FJ583837
		HLC-11030	TZAIC322-05	FJ583838
		HLC-11034	TZAIC326-05	FJ583839
		HLC-11033	TZAIC325-05	FJ583840
	Parupeneus cyclostomus	HLC-11083	TZAIC375-05	FJ583841
		HLC-10883	TZAIC183-05	FJ583842
Family Opistognathidae	*Opistognathus aurifrons*	HLC-13136	TZAIB513-06	FJ583756
		HLC-11742	TZAIC680-06	FJ583757
		HLC-13132	TZAIB509-06	FJ583758
		HLC-13133	TZAIB510-06	FJ583759
		HLC-13134	TZAIB511-06	FJ583760
		HLC-13135	TZAIB512-06	FJ583761
		HLC-11741	TZAIC679-06	FJ583762
	Opistognathus punctatus	HLC-12140	TZAIB029-06	FJ583763
		HLC-12141	TZAIB030-06	FJ583764
		HLC-13288	TZAIB665-06	FJ583765
		HLC-11789	TZAIC727-06	FJ583766
	Opistognathus rosenblatti	HLC-15123	TZAIB805-07	FJ583767
		HLC-15124	TZAIB806-07	FJ583768
Family Plesiopidae	*Calloplesiops altivelis*	HLC-11930	TZAIB195-06	FJ582909
		HLC-12137	TZAIB026-06	FJ582910
		HLC-11704	TZAIC636-06	FJ582911
		HLC-11703	TZAIC635-06	FJ582912
		HLC-11702	TZAIC634-06	FJ582913
Family Pomacanthidae	*Apolemichthys trimaculatus*	HLC-12340	TZAIB323-06	FJ582868
		HLC-12019	TZAIB096-06	FJ582869
		HLC-12020	TZAIB097-06	FJ582870
		HLC-12021	TZAIB098-06	FJ582871
		HLC-12022	TZAIB099-06	FJ582872
	Apolemichthys xanthurus	HLC-12385	TZAIB368-06	FJ582873
		HLC-13188	TZAIB565-06	FJ582874
	Centropyge sp.	HLC-11631	TZAIC563-06	FJ582927
	Centropyge argi	HLC-15151	TZAIB833-07	FJ582928
		HLC-15152	TZAIB834-07	FJ582929
		HLC-15153	TZAIB835-07	FJ582930
		HLC-15154	TZAIB836-07	FJ582931
		HLC-13279	TZAIB656-06	FJ582932
		HLC-11594	TZAIC526-06	FJ582933
		HLC-10948	TZAIC248-05	FJ582934
		HLC-10947	TZAIC247-05	FJ582935
		HLC-10946	TZAIC246-05	FJ582936
		HLC-10945	TZAIC245-05	FJ582937
		HLC-10944	TZAIC244-05	FJ582938
	Centropyge bicolor	HLC-12122	TZAIB011-06	FJ582939
		HLC-11763	TZAIC701-06	FJ582940
		HLC-11762	TZAIC700-06	FJ582941
		HLC-11761	TZAIC699-06	FJ582942
		HLC-11156	TZAIC448-05	FJ582943
		HLC-11155	TZAIC447-05	FJ582944

	HLC-10942	TZAIC242-05	FJ582945
	HLC-10880	TZAIC180-05	FJ582946
	HLC-10879	TZAIC179-05	FJ582947
Centropyge bispinosa	HLC-12029	TZAIB106-06	FJ582948
	HLC-11680	TZAIC612-06	FJ582949
	HLC-11109	TZAIC401-05	FJ582950
	HLC-11108	TZAIC400-05	FJ582951
	HLC-11107	TZAIC399-05	FJ582952
	HLC-11106	TZAIC398-05	FJ582953
	HLC-10932	TZAIC232-05	FJ582954
	HLC-10931	TZAIC231-05	FJ582955
	HLC-10930	TZAIC230-05	FJ582956
	HLC-10929	TZAIC229-05	FJ582957
	HLC-10928	TZAIC228-05	FJ582958
Centropyge eibli	HLC-15216	TZAIB886-07	FJ582959
	HLC-15215	TZAIB885-07	FJ582960
Centropyge ferrugata	HLC-12060	TZAIB137-06	FJ582961
	HLC-12059	TZAIB136-06	FJ582962
	HLC-12058	TZAIB135-06	FJ582963
Centropyge flavissima	HLC-10882	TZAIC182-05	FJ582964
Centropyge heraldi	HLC-15119	TZAIB801-07	FJ582965
	HLC-15168	TZAIB850-07	FJ582966
	HLC-12131	TZAIB020-06	FJ582967
	HLC-11144	TZAIC436-05	FJ582968
	HLC-11143	TZAIC435-05	FJ582969
	HLC-11027	TZAIC319-05	FJ582970
	HLC-11026	TZAIC318-05	FJ582971
	HLC-10794	TZAIC094-05	FJ582972
	HLC-10793	TZAIC093-05	FJ582973
Centropyge loricula	HLC-11732	TZAIC670-06	FJ582974
	HLC-11731	TZAIC669-06	FJ582975
	HLC-11707	TZAIC639-06	FJ582976
Centropyge multicolor	HLC-13130	TZAIB507-06	FJ582977
	HLC-13121	TZAIB498-06	FJ582978
Centropyge multifasciata	HLC-11112	TZAIC404-05	FJ582979
Centropyge nox	HLC-12004	TZAIB269-06	FJ582980
	HLC-12005	TZAIB270-06	FJ582981
	HLC-11907	TZAIC845-06	FJ582982
	HLC-10943	TZAIC243-05	FJ582983
Centropyge potteri	HLC-10884	TZAIC184-05	FJ582984
	HLC-12316	TZAIB299-06	FJ582985
Centropyge tibicen	HLC-15054	TZAIB741-06	FJ582986
	HLC-15055	TZAIB742-06	FJ582987
	HLC-15056	TZAIB743-06	FJ582988
	HLC-12286	TZAIC927-06	FJ582989
	HLC-12272	TZAIC913-06	FJ582990
	HLC-11139	TZAIC431-05	FJ582991
	HLC-11138	TZAIC430-05	FJ582992
	HLC-10802	TZAIC102-05	FJ582993
Centropyge venustus	HLC-11173	TZAIC661-06	FJ582994
	HLC-11744	TZAIC682-06	FJ582995
Centropyge vrolikii	HLC-11078	TZAIC370-05	FJ582996
	HLC-11639	TZAIC571-06	FJ582997
	HLC-11638	TZAIC570-06	FJ582998
	HLC-11079	TZAIC371-05	FJ582999
	HLC-11077	TZAIC369-05	FJ583000
	HLC-11065	TZAIC357-05	FJ583001
	HLC-11715	TZAIC647-06	FJ583002
Chaetodontoplus caeruleopunctatus	HLC-11935	TZAIB200-06	FJ583090
	HLC-12383	TZAIB366-06	FJ583091
	HLC-13210	TZAIB587-06	FJ583092
Chaetodontoplus melanosoma	HLC-13083	TZAIB460-06	FJ583093
Chaetodontoplus mesoleucus	HLC-12343	TZAIB326-06	FJ583094
Chaetodontoplus septentrionalis	HLC-15036	TZAIB735-06	FJ583095
	HLC-15037	TZAIB736-06	FJ583096
	HLC-15038	TZAIB737-06	FJ583097
	HLC-13248	TZAIB625-06	FJ583098
	HLC-13249	TZAIB626-06	FJ583099
	HLC-10889	TZAIC189-05	FJ583100
Genicanthus lamarck	HLC-12149	TZAIB038-06	FJ583415
	HLC-12150	TZAIB039-06	FJ583416
	HLC-12151	TZAIB040-06	FJ583417
	HLC-12152	TZAIB041-06	FJ583418
	HLC-12153	TZAIB042-06	FJ583419
	HLC-15229	TZAIB899-07	FJ583420
	HLC-15230	TZAIB900-07	FJ583421
	HLC-15231	TZAIB901-07	FJ583422
	HLC-15232	TZAIB902-07	FJ583423
Genicanthus melanospilos	HLC-11061	TZAIC353-05	FJ583424

		HLC-12135	TZAIB024-06	FJ583425
		HLC-12280	TZAIC921-06	FJ583426
	Genicanthus watanabei	HLC-12339	TZAIB322-06	FJ583427
	Holacanthus ciliaris	HLC-12425	TZAIB740-06	FJ583558
		HLC-12424	TZAIB739-06	FJ583559
		HLC-12423	TZAIB738-06	FJ583560
		HLC-13272	TZAIB649-06	FJ583561
		HLC-13055	TZAIB432-06	FJ583562
	Holacanthus tricolor	HLC-11992	TZAIB257-06	FJ583563
		HLC-11993	TZAIB258-06	FJ583564
		HLC-11994	TZAIB259-06	FJ583565
		HLC-11995	TZAIB260-06	FJ583566
		HLC-11996	TZAIB261-06	FJ583567
		HLC-15109	TZAIB792-07	FJ583568
	Pomacanthus annularis	HLC-12420	TZAIB744-06	FJ583875
		HLC-12123	TZAIB012-06	FJ583876
	Pomacanthus imperator	HLC-11794	TZAIC732-06	FJ583877
		HLC-11938	TZAIB203-06	FJ583878
		HLC-12281	TZAIC922-06	FJ583879
		HLC-11710	TZAIC642-06	FJ583880
		HLC-11141	TZAIC433-05	FJ583881
		HLC-10801	TZAIC101-05	FJ583882
	Pomacanthus navarchus	HLC-15226	TZAIB896-07	FJ583883
	Pomacanthus paru	HLC-12422	TZAIB745-06	FJ583884
		HLC-13063	TZAIB440-06	FJ583885
	Pomacanthus semicirculatus	HLC-11961	TZAIB226-06	FJ583886
		HLC-12297	TZAIC938-06	FJ583887
	Pygoplites diacanthus	HLC-12391	TZAIB374-06	FJ584045
		HLC-12030	TZAIB107-06	FJ584046
		HLC-12031	TZAIB108-06	FJ584047
		HLC-11066	TZAIC358-05	FJ584048
Family Pomacentridae	*Abudefduf vaigiensis*	HLC-10821	TZAIC121-05	FJ582646
		HLC-15040	TZAIB684-06	FJ582645
	Amphiprion akallopisos	HLC-13056	TZAIB433-06	FJ582726
		HLC-13143	TZAIB520-06	FJ582727
		HLC-12190	TZAIB079-06	FJ582728
		HLC-15033	TZAIB747-06	FJ582729
		HLC-15034	TZAIB748-06	FJ582730
		HLC-15075	TZAIB749-06	FJ582731
		HLC-13165	TZAIB542-06	FJ582732
		HLC-15130	TZAIB812-07	FJ582733
		HLC-11075	TZAIC367-05	FJ582734
		HLC-11073	TZAIC365-05	FJ582735
		HLC-11072	TZAIC364-05	FJ582736
		HLC-11071	TZAIC363-05	FJ582737
	Amphiprion chrysogaster	HLC-12402	TZAIB385-06	FJ582738
		HLC-11745	TZAIC683-06	FJ582739
		HLC-12302	TZAIB285-06	FJ582740
		HLC-12303	TZAIB286-06	FJ582741
		HLC-12304	TZAIB287-06	FJ582742
		HLC-12305	TZAIB288-06	FJ582743
		HLC-12306	TZAIB289-06	FJ582744
		HLC-11749	TZAIC687-06	FJ582745
		HLC-11748	TZAIC686-06	FJ582746
		HLC-11747	TZAIC685-06	FJ582747
		HLC-11746	TZAIC684-06	FJ582748
		HLC-10913	TZAIC213-05	FJ582749
		HLC-10914	TZAIC214-05	FJ582750
	Amphiprion chrysopterus	HLC-11684	TZAIC616-06	FJ582751
		HLC-11686	TZAIC618-06	FJ582752
		HLC-11685	TZAIC617-06	FJ582753
		HLC-11683	TZAIC615-06	FJ582754
		HLC-11682	TZAIC614-06	FJ582755
	Amphiprion clarkii	HLC-11895	TZAIC833-06	FJ582756
		HLC-10912	TZAIC212-05	FJ582757
		HLC-10910	TZAIC210-05	FJ582758
	Amphiprion frenatus	HLC-11555	TZAIC487-06	FJ582759
	Amphiprion latifasciatus	HLC-10911	TZAIC211-05	FJ582760
	Amphiprion melanopus	HLC-11554	TZAIC486-06	FJ582761
		HLC-10846	TZAIC146-05	FJ582762
		HLC-10845	TZAIC145-05	FJ582763
		HLC-10843	TZAIC143-05	FJ582764
		HLC-12164	TZAIB053-06	FJ582765
		HLC-12165	TZAIB054-06	FJ582766
		HLC-12166	TZAIB055-06	FJ582767
		HLC-12167	TZAIB056-06	FJ582768
		HLC-12168	TZAIB057-06	FJ582769
		HLC-11653	TZAIC585-06	FJ582770
		HLC-11137	TZAIC429-05	FJ582771

	HLC-10844	TZAIC144-05	FJ582772
	HLC-10842	TZAIC142-05	FJ582773
Amphiprion nigripes	HLC-11570	TZAIC502-06	FJ582774
	HLC-11123	TZAIC415-05	FJ582775
	HLC-11121	TZAIC413-05	FJ582776
	HLC-11122	TZAIC414-05	FJ582777
	HLC-10774	TZAIC074-05	FJ582778
	HLC-10775	TZAIC075-05	FJ582779
	HLC-10772	TZAIC072-05	FJ582780
	HLC-10773	TZAIC073-05	FJ582781
	HLC-10776	TZAIC076-05	FJ582782
	HLC-10921	TZAIC221-05	FJ582783
	HLC-10920	TZAIC220-05	FJ582784
Amphiprion ocellaris	HLC-10909	TZAIC209-05	FJ582785
	HLC-10770	TZAIC070-05	FJ582786
	HLC-10767	TZAIC067-05	FJ582787
	HLC-10768	TZAIC068-05	FJ582788
	HLC-10769	TZAIC069-05	FJ582789
	HLC-10771	TZAIC071-05	FJ582790
	HLC-10905	TZAIC205-05	FJ582791
	HLC-10908	TZAIC208-05	FJ582792
	HLC-10906	TZAIC206-05	FJ582793
	HLC-10907	TZAIC207-05	FJ582794
Amphiprion perideraion	HLC-12170	TZAIB059-06	FJ582795
	HLC-12169	TZAIB058-06	FJ582796
	HLC-12171	TZAIB060-06	FJ582797
	HLC-12172	TZAIB061-06	FJ582798
	HLC-12173	TZAIB062-06	FJ582799
	HLC-11564	TZAIC496-06	FJ582800
	HLC-11074	TZAIC366-05	FJ582801
	HLC-10916	TZAIC216-05	FJ582802
	HLC-10918	TZAIC218-05	FJ582803
	HLC-10917	TZAIC217-05	FJ582804
	HLC-10915	TZAIC215-05	FJ582805
	HLC-10919	TZAIC219-05	FJ582806
Amphiprion polymnus	HLC-11583	TZAIC515-06	FJ582807
	HLC-13296	TZAIB750-06	FJ582808
	HLC-13297	TZAIB751-06	FJ582809
	HLC-13298	TZAIB752-06	FJ582810
	HLC-13299	TZAIB753-06	FJ582811
	HLC-13300	TZAIB754-06	FJ582812
	HLC-10839	TZAIC139-05	FJ582813
Amphiprion sandaracinos	HLC-11563	TZAIC495-06	FJ582814
	HLC-11562	TZAIC494-06	FJ582815
	HLC-11561	TZAIC493-06	FJ582816
Amphiprion sebae	HLC-10836	TZAIC136-05	FJ582817
	HLC-10834	TZAIC134-05	FJ582818
	HLC-10835	TZAIC135-05	FJ582819
	HLC-10832	TZAIC132-05	FJ582820
	HLC-13044	TZAIB421-06	FJ582821
	HLC-13045	TZAIB422-06	FJ582822
	HLC-13046	TZAIB423-06	FJ582823
	HLC-13292	TZAIB669-06	FJ582824
	HLC-11894	TZAIC832-06	FJ582825
	HLC-12242	TZAIC883-06	FJ582826
	HLC-12241	TZAIC882-06	FJ582827
	HLC-12240	TZAIC881-06	FJ582828
	HLC-12239	TZAIC880-06	FJ582829
	HLC-10833	TZAIC133-05	FJ582830
Chromis atripectoralis	HLC-11602	TZAIC534-06	FJ583145
Chromis caerulea	HLC-10922	TZAIC222-05	FJ583146
	HLC-10924	TZAIC224-05	FJ583147
	HLC-10926	TZAIC226-05	FJ583148
Chromis cyanea	HLC-11979	TZAIB244-06	FJ583149
	HLC-11975	TZAIB240-06	FJ583150
	HLC-11976	TZAIB241-06	FJ583151
	HLC-11977	TZAIB242-06	FJ583152
	HLC-11978	TZAIB243-06	FJ583153
Chromis insolata	HLC-13050	TZAIB427-06	FJ583154
	HLC-13051	TZAIB428-06	FJ583155
	HLC-13052	TZAIB429-06	FJ583156
	HLC-13053	TZAIB430-06	FJ583157
Chromis margaritifer	HLC-12174	TZAIB063-06	FJ583158
	HLC-12175	TZAIB064-06	FJ583159
	HLC-12176	TZAIB065-06	FJ583160
	HLC-12177	TZAIB066-06	FJ583161
	HLC-12178	TZAIB067-06	FJ583162
Chromis viridis	HLC-11601	TZAIC533-06	FJ583163
	HLC-11600	TZAIC532-06	FJ583164

	HLC-11599	TZAIC531-06	FJ583165
	HLC-11598	TZAIC530-06	FJ583166
	HLC-10925	TZAIC225-05	FJ583167
Chrysiptera cyanea	HLC-10752	TZAIC052-05	FJ583168
	HLC-11944	TZAIB209-06	FJ583169
	HLC-11941	TZAIB206-06	FJ583170
	HLC-11942	TZAIB207-06	FJ583171
	HLC-11943	TZAIB208-06	FJ583172
	HLC-11945	TZAIB210-06	FJ583173
	HLC-10754	TZAIC054-05	FJ583174
	HLC-11637	TZAIC569-06	FJ583175
	HLC-11636	TZAIC568-06	FJ583176
	HLC-11635	TZAIC567-06	FJ583177
	HLC-11634	TZAIC566-06	FJ583178
	HLC-11633	TZAIC565-06	FJ583179
	HLC-10756	TZAIC056-05	FJ583180
	HLC-10755	TZAIC055-05	FJ583181
	HLC-10753	TZAIC053-05	FJ583182
Chrysiptera galba	HLC-12183	TZAIB072-06	FJ583183
	HLC-12179	TZAIB068-06	FJ583184
	HLC-12180	TZAIB069-06	FJ583185
	HLC-12181	TZAIB070-06	FJ583186
	HLC-12182	TZAIB071-06	FJ583187
Chrysiptera parasema	HLC-10748	TZAIC048-05	FJ583188
	HLC-10747	TZAIC047-05	FJ583189
	HLC-10751	TZAIC051-05	FJ583190
	HLC-10750	TZAIC050-05	FJ583191
	HLC-10749	TZAIC049-05	FJ583192
Chrysiptera rex	HLC-10837	TZAIC137-05	FJ583193
	HLC-11617	TZAIC549-06	FJ583194
	HLC-11616	TZAIC548-06	FJ583195
	HLC-10838	TZAIC138-05	FJ583196
Chrysiptera starcki	HLC-13253	TZAIB630-06	FJ583197
	HLC-12154	TZAIB043-06	FJ583198
	HLC-13255	TZAIB632-06	FJ583199
	HLC-13254	TZAIB631-06	FJ583200
	HLC-13252	TZAIB629-06	FJ583201
	HLC-11644	TZAIC576-06	FJ583202
	HLC-11643	TZAIC575-06	FJ583203
Chrysiptera talboti	HLC-11826	TZAIC764-06	FJ583204
	HLC-11827	TZAIC765-06	FJ583205
	HLC-11824	TZAIC762-06	FJ583206
	HLC-11823	TZAIC761-06	FJ583207
Chrysiptera taupou	HLC-11146	TZAIC438-05	FJ583208
	HLC-11147	TZAIC439-05	FJ583209
	HLC-11148	TZAIC440-05	FJ583210
	HLC-11149	TZAIC441-05	FJ583211
	HLC-10904	TZAIC204-05	FJ583212
Dascyllus aruanus	HLC-10829	TZAIC129-05	FJ583321
	HLC-10830	TZAIC130-05	FJ583322
	HLC-10828	TZAIC128-05	FJ583323
	HLC-10831	TZAIC131-05	FJ583324
Dascyllus flavicaudus	HLC-11875	TZAIC813-06	FJ583325
Dascyllus melanurus	HLC-11956	TZAIB221-06	FJ583326
	HLC-11957	TZAIB222-06	FJ583327
	HLC-11958	TZAIB223-06	FJ583328
	HLC-11959	TZAIB224-06	FJ583329
	HLC-11960	TZAIB225-06	FJ583330
Dascyllus reticulatus	HLC-11877	TZAIC815-06	FJ583331
	HLC-11874	TZAIC812-06	FJ583332
Dascyllus trimaculatus	HLC-11042	TZAIC334-05	FJ583333
	HLC-11044	TZAIC336-05	FJ583334
	HLC-11043	TZAIC335-05	FJ583335
	HLC-11041	TZAIC333-05	FJ583336
	HLC-11045	TZAIC337-05	FJ583337
	HLC-11064	TZAIC356-05	FJ583338
Dischistodus prosopotaenia	HLC-12214	TZAIC855-06	FJ583365
Dischistodus pseudochrysopoecilus	HLC-12215	TZAIC856-06	FJ583366
	HLC-12216	TZAIC857-06	FJ583367
	HLC-12213	TZAIC854-06	FJ583368
	HLC-12212	TZAIC853-06	FJ583369
Microspathodon chrysurus	HLC-12015	TZAIB280-06	FJ583659
	HLC-12013	TZAIB278-06	FJ583660
	HLC-12014	TZAIB279-06	FJ583661
	HLC-12016	TZAIB281-06	FJ583662
Neoglyphidodon melas	HLC-10899	TZAIC199-05	FJ583724
	HLC-10901	TZAIC201-05	FJ583725
	HLC-10902	TZAIC202-05	FJ583726
	HLC-10900	TZAIC200-05	FJ583727

		HLC-10903	TZAIC203-05	FJ583728
	Neoglyphidodon nigroris	HLC-12034	TZAIB111-06	FJ583729
		HLC-12035	TZAIB112-06	FJ583730
		HLC-12036	TZAIB113-06	FJ583731
		HLC-12037	TZAIB114-06	FJ583732
		HLC-12038	TZAIB115-06	FJ583733
	Neoglyphidodon oxyodon	HLC-10823	TZAIC123-05	FJ583734
		HLC-10825	TZAIC125-05	FJ583735
		HLC-10822	TZAIC122-05	FJ583736
		HLC-10826	TZAIC126-05	FJ583737
		HLC-10824	TZAIC124-05	FJ583738
	Pomacentrus alleni	HLC-12194	TZAIB083-06	FJ583888
		HLC-12196	TZAIB085-06	FJ583889
		HLC-12192	TZAIB081-06	FJ583890
		HLC-12193	TZAIB082-06	FJ583891
		HLC-12195	TZAIB084-06	FJ583892
	Pomacentrus caeruleus	HLC-11754	TZAIC692-06	FJ583893
		HLC-11753	TZAIC691-06	FJ583894
		HLC-11752	TZAIC690-06	FJ583895
		HLC-11751	TZAIC689-06	FJ583896
		HLC-11750	TZAIC688-06	FJ583897
	Pomacentrus coelestis	HLC-11819	TZAIC757-06	FJ583898
		HLC-11818	TZAIC756-06	FJ583899
		HLC-11817	TZAIC755-06	FJ583900
		HLC-11816	TZAIC754-06	FJ583901
	Pomacentrus moluccensis	HLC-11082	TZAIC374-05	FJ583902
		HLC-11822	TZAIC760-06	FJ583903
		HLC-11821	TZAIC759-06	FJ583904
		HLC-11157	TZAIC449-05	FJ583905
		HLC-11081	TZAIC373-05	FJ583906
		HLC-11080	TZAIC372-05	FJ583907
	Pomacentrus nagasakiensis	HLC-12225	TZAIC866-06	FJ583908
		HLC-12224	TZAIC865-06	FJ583909
		HLC-12223	TZAIC864-06	FJ583910
		HLC-12222	TZAIC863-06	FJ583911
	Premnas biaculeatus	HLC-11690	TZAIC622-06	FJ583912
		HLC-11766	TZAIC704-06	FJ583913
		HLC-11689	TZAIC621-06	FJ583914
		HLC-11688	TZAIC620-06	FJ583915
		HLC-11687	TZAIC619-06	FJ583916
		HLC-11127	TZAIC419-05	FJ583917
		HLC-11125	TZAIC417-05	FJ583918
		HLC-11128	TZAIC420-05	FJ583919
		HLC-11126	TZAIC418-05	FJ583920
		HLC-11069	TZAIC361-05	FJ583921
Family Pseudochromidae	*Labracinus cyclophthalmus*	HLC-12024	TZAIB101-06	FJ583584
		HLC-11729	TZAIC667-06	FJ583585
	Labracinus lineatus	HLC-10746	TZAIC046-05	FJ583586
	Pseudochromis diadema	HLC-12042	TZAIB119-06	FJ583978
		HLC-12041	TZAIB118-06	FJ583979
	Pseudochromis fuscus	HLC-11661	TZAIC593-06	FJ583980
		HLC-11660	TZAIC592-06	FJ583981
	Pseudochromis paccagnellae	HLC-12189	TZAIB078-06	FJ583982
		HLC-10985	TZAIC465-05	FJ583983
		HLC-10986	TZAIC466-05	FJ583984
		HLC-10987	TZAIC467-05	FJ583985
	Pseudochromis porphyreus	HLC-11053	TZAIC345-05	FJ583986
		HLC-11054	TZAIC346-05	FJ583987
		HLC-11051	TZAIC343-05	FJ583988
		HLC-11052	TZAIC344-05	FJ583989
		HLC-11055	TZAIC347-05	FJ583990
Family Scaridae	*Leptoscarus vaigiensis*	HLC-12386	TZAIB369-06	FJ583627
	Scarus iseri	HLC-12100	TZAIB177-06	FJ584081
		HLC-12101	TZAIB178-06	FJ584082
	Scarus vetula	HLC-15210	TZAIB880-07	FJ584083
		HLC-11939	TZAIB204-06	FJ584084
		HLC-11940	TZAIB205-06	FJ584085
Family Scatophagidae	*Scatophagus argus*	HLC-15211	TZAIB881-07	FJ584086
		HLC-15212	TZAIB882-07	FJ584087
		HLC-15213	TZAIB883-07	FJ584088
		HLC-15214	TZAIB884-07	FJ584089
		HLC-11039	TZAIC331-05	FJ584090
Family Sciaenidae	*Equetus lanceolatus*	HLC-11980	TZAIB245-06	FJ583400
		HLC-13119	TZAIB496-06	FJ583401
	Equetus punctatus	HLC-11981	TZAIB246-06	FJ583402
		HLC-15057	TZAIB755-06	FJ583403
		HLC-15058	TZAIB756-06	FJ583404
	Pareques acuminatus	HLC-12358	TZAIB341-06	FJ583830
		HLC-13294	TZAIB671-06	FJ583831

Family Serranidae	*Cephalopholis formosa*	HLC-12384	TZAIB367-06	FJ583003
		HLC-15117	TZAIB799-07	FJ583004
	Cephalopholis fulva	HLC-13111	TZAIB488-06	FJ583005
		HLC-13112	TZAIB489-06	FJ583006
		HLC-13113	TZAIB490-06	FJ583007
		HLC-13114	TZAIB491-06	FJ583008
		HLC-13115	TZAIB492-06	FJ583009
	Cephalopholis leopardus	HLC-13138	TZAIB515-06	FJ583010
	Cephalopholis miniata	HLC-15187	TZAIB857-07	FJ583011
	Cephalopholis urodeta	HLC-12146	TZAIB035-06	FJ583012
		HLC-12372	TZAIB355-06	FJ583013
		HLC-12373	TZAIB356-06	FJ583014
		HLC-13139	TZAIB516-06	FJ583015
	Cromileptes altivelis	HLC-11835	TZAIC773-06	FJ583275
		HLC-11834	TZAIC772-06	FJ583276
		HLC-11833	TZAIC771-06	FJ583277
		HLC-11832	TZAIC770-06	FJ583278
		HLC-11828	TZAIC766-06	FJ583279
	Epinephelus adscensionis	HLC-13067	TZAIB444-06	FJ583396
	Epinephelus ongus	HLC-12321	TZAIB304-06	FJ583397
		HLC-13117	TZAIB494-06	FJ583398
		HLC-13118	TZAIB495-06	FJ583399
	Hypoplectrus puella	HLC-13033	TZAIB410-06	FJ583577
		HLC-13036	TZAIB413-06	FJ583578
		HLC-13037	TZAIB414-06	FJ583579
		HLC-13038	TZAIB415-06	FJ583580
		HLC-13175	TZAIB552-06	FJ583581
		HLC-13207	TZAIB584-06	FJ583582
	Mycteroperca interstitialis	HLC-13116	TZAIB493-06	FJ583668
	Nemanthias carberryi	HLC-12342	TZAIB325-06	FJ583695
		HLC-10988	TZAIC468-05	FJ583696
		HLC-10989	TZAIC469-05	FJ583697
		HLC-10992	TZAIC284-05	FJ583698
		HLC-10991	TZAIC283-05	FJ583699
		HLC-10990	TZAIC470-05	FJ583700
	Plectropomus maculatus	HLC-13064	TZAIB441-06	FJ583869
	Pseudanthias huchtii	HLC-12136	TZAIB025-06	FJ583925
	Pseudanthias lori	HLC-13101	TZAIB478-06	FJ583926
		HLC-13102	TZAIB479-06	FJ583927
		HLC-13103	TZAIB480-06	FJ583928
		HLC-13104	TZAIB481-06	FJ583929
		HLC-13105	TZAIB482-06	FJ583930
	Pseudanthias pascalus	HLC-12312	TZAIB295-06	FJ583931
		HLC-12313	TZAIB296-06	FJ583932
		HLC-12314	TZAIB297-06	FJ583933
		HLC-10981	TZAIC281-05	FJ583934
	Pseudanthias pleurotaenia	HLC-11626	TZAIC558-06	FJ583935
		HLC-11624	TZAIC556-06	FJ583936
		HLC-10766	TZAIC066-05	FJ583937
		HLC-10765	TZAIC065-05	FJ583938
	Pseudanthias squamipinnis	HLC-11694	TZAIC626-06	FJ583939
		HLC-12093	TZAIB170-06	FJ583940
		HLC-12012	TZAIB277-06	FJ583941
		HLC-12094	TZAIB171-06	FJ583942
		HLC-12095	TZAIB172-06	FJ583943
		HLC-12096	TZAIB173-06	FJ583944
		HLC-15233	TZAIB903-07	FJ583945
		HLC-15234	TZAIB904-07	FJ583946
		HLC-15235	TZAIB905-07	FJ583947
		HLC-11723	TZAIC655-06	FJ583948
		HLC-11719	TZAIC651-06	FJ583949
		HLC-11718	TZAIC650-06	FJ583950
		HLC-11695	TZAIC627-06	FJ583951
		HLC-11692	TZAIC624-06	FJ583952
		HLC-11788	TZAIC726-06	FJ583953
		HLC-11678	TZAIC610-06	FJ583954
		HLC-11641	TZAIC573-06	FJ583955
		HLC-11640	TZAIC572-06	FJ583956
	Pseudanthias truncatus	HLC-11625	TZAIC557-06	FJ583957
		HLC-10764	TZAIC064-05	FJ583958
		HLC-10763	TZAIC063-05	FJ583959
		HLC-10762	TZAIC062-05	FJ583960
	Serranocirrhitus latus	HLC-11955	TZAIB220-06	FJ584094
		HLC-10820	TZAIC120-05	FJ584095
		HLC-11954	TZAIB219-06	FJ584096
		HLC-11133	TZAIC425-05	FJ584097
		HLC-11134	TZAIC426-05	FJ584098
	Serranus baldwini	HLC-12348	TZAIB331-06	FJ584099
		HLC-12349	TZAIB332-06	FJ584100

			HLC-12350	TZAIB333-06	FJ584101
			HLC-12351	TZAIB334-06	FJ584102
			HLC-13081	TZAIB458-06	FJ584103
		Serranus tabacarius	HLC-13202	TZAIB579-06	FJ584104
		Serranus tigrinus	HLC-12337	TZAIB320-06	FJ584105
			HLC-12338	TZAIB321-06	FJ584106
			HLC-13144	TZAIB521-06	FJ584107
		Variola albimarginata	HLC-12364	TZAIB347-06	FJ584241
		Variola louti	HLC-15129	TZAIB811-07	FJ584242
	Family Siganidae	*Siganus puellus*	HLC-13026	TZAIB403-06	FJ584108
		Siganus virgatus	HLC-13284	TZAIB661-06	FJ584109
			HLC-15107	TZAIB790-07	FJ584110
			HLC-15108	TZAIB791-07	FJ584111
			HLC-15185	TZAIB855-07	FJ584112
			HLC-10743	TZAIC043-05	FJ584113
		Siganus vulpinus	HLC-10741	TZAIC041-05	FJ584114
			HLC-10742	TZAIC042-05	FJ584115
			HLC-10740	TZAIC040-05	FJ584116
			HLC-10738	TZAIC038-05	FJ584117
			HLC-10739	TZAIC039-05	FJ584118
	Family Sphyraenidae	*Sphyraena barracuda*	HLC-15190	TZAIB860-07	FJ584124
			HLC-15191	TZAIB861-07	FJ584125
	Family Zanclidae	*Zanclus cornutus*	HLC-11758	TZAIC696-06	FJ584250
			HLC-15015	TZAIB757-06	FJ584251
			HLC-15016	TZAIB758-06	FJ584252
			HLC-15017	TZAIB759-06	FJ584253
			HLC-15018	TZAIB760-06	FJ584254
			HLC-15019	TZAIB761-06	FJ584255
			HLC-11760	TZAIC698-06	FJ584256
			HLC-11759	TZAIC697-06	FJ584257
			HLC-10798	TZAIC098-05	FJ584258
			HLC-10797	TZAIC097-05	FJ584259
			HLC-10795	TZAIC095-05	FJ584260
			HLC-10796	TZAIC096-05	FJ584261
			HLC-10799	TZAIC099-05	FJ584262
Order Pleuronectiformes					
	Family Achiridae	*Achirus lineatus*	HLC-12106	TZAIB183-06	FJ582703
Order Scorpaeniformes					
	Family Dactylopteridae	*Dactyloptena orientalis*	HLC-12232	TZAIC873-06	FJ583311
			HLC-12236	TZAIC877-06	FJ583312
			HLC-12235	TZAIC876-06	FJ583313
			HLC-12234	TZAIC875-06	FJ583314
			HLC-12233	TZAIC874-06	FJ583315
	Family Scorpaenidae	*Dendrochirus biocellatus*	HLC-11084	TZAIC376-05	FJ583342
		Dendrochirus brachypterus	HLC-12204	TZAIB093-06	FJ583343
			HLC-13219	TZAIB596-06	FJ583344
			HLC-13220	TZAIB597-06	FJ583345
			HLC-13221	TZAIB598-06	FJ583346
			HLC-13222	TZAIB599-06	FJ583347
		Dendrochirus zebra	HLC-11610	TZAIC542-06	FJ583348
			HLC-12203	TZAIB092-06	FJ583349
			HLC-13293	TZAIB670-06	FJ583350
			HLC-12291	TZAIC932-06	FJ583351
			HLC-12290	TZAIC931-06	FJ583352
			HLC-TSZ-011	TZAIC011-05	FJ583353
			HLC-TSZ-012	TZAIC012-05	FJ583354
		Pterois andover	HLC-11569	TZAIC501-06	FJ584016
		Pterois antennata	HLC-13212	TZAIB589-06	FJ584017
			HLC-13213	TZAIB590-06	FJ584018
			HLC-15224	TZAIB894-07	FJ584019
			HLC-15225	TZAIB895-07	FJ584020
			HLC-12296	TZAIC937-06	FJ584021
			HLC-12295	TZAIC936-06	FJ584022
			HLC-12294	TZAIC935-06	FJ584023
			HLC-11568	TZAIC500-06	FJ584024
			HLC-11151	TZAIC443-05	FJ584025
		Pterois miles	HLC-12395	TZAIB378-06	FJ584026
			HLC-12394	TZAIB377-06	FJ584027
			HLC-12138	TZAIB027-06	FJ584028
			HLC-11859	TZAIC797-06	FJ584029
			HLC-12293	TZAIC934-06	FJ584030
			HLC-12292	TZAIC933-06	FJ584031
			HLC-TSZ-014	TZAIC014-05	FJ584032
			HLC-TSZ-016	TZAIC016-05	FJ584033
			HLC-TSZ-015	TZAIC015-05	FJ584034
		Pterois mombasae	HLC-11933	TZAIB198-06	FJ584035
			HLC-11931	TZAIB196-06	FJ584036
			HLC-11932	TZAIB197-06	FJ584037
			HLC-TSZ-009	TZAIC009-05	FJ584038

			HLC-TSZ-010	TZAIC010-05	FJ584039
		Pterois volitans	HLC-13089	TZAIB466-06	FJ584040
			HLC-15005	TZAIB691-06	FJ584041
			HLC-15072	TZAIB692-06	FJ584042
			HLC-15128	TZAIB810-07	FJ584043
			HLC-12264	TZAIC905-06	FJ584044
		Scorpaena brasiliensis	HLC-12049	TZAIB126-06	FJ584091
			HLC-12285	TZAIC926-06	FJ584092
		Taenianotus triacanthus	HLC-12369	TZAIB352-06	FJ584154
			HLC-13280	TZAIB657-06	FJ584155
			HLC-13281	TZAIB658-06	FJ584156
			HLC-13027	TZAIB404-06	FJ584157
			HLC-13040	TZAIB417-06	FJ584158
			HLC-15087	TZAIB693-06	FJ584159
			HLC-13201	TZAIB578-06	FJ584160
			HLC-13215	TZAIB592-06	FJ584161
			HLC-13216	TZAIB593-06	FJ584162
	Family Synanceiidae	*Synanceia verrucosa*	HLC-13203	TZAIB580-06	FJ584136
	Family Triglidae	*Prionotus sp.*	HLC-12130	TZAIB019-06	FJ583922
		Prionotus ophryas	HLC-12090	TZAIB167-06	FJ583923
			HLC-12091	TZAIB168-06	FJ583924
Order Syngnathiformes					
	Family Aulostomidae	*Aulostomus chinensis*	HLC-13062	TZAIB439-06	FJ582892
	Family Syngnathidae	*Cosmocampus elucens*	HLC-12288	TZAIC929-06	FJ583271
			HLC-12287	TZAIC928-06	FJ583272
			HLC-12238	TZAIC879-06	FJ583273
			HLC-12237	TZAIC878-06	FJ583274
		Doryrhamphus dactyliophorus	HLC-11925	TZAIB190-06	FJ583370
			HLC-11926	TZAIB191-06	FJ583371
			HLC-11927	TZAIB192-06	FJ583372
			HLC-13034	TZAIB411-06	FJ583373
			HLC-11018	TZAIC310-05	FJ583374
		Doryrhamphus pessuliferus	HLC-12300	TZAIB283-06	FJ583375
			HLC-12318	TZAIB301-06	FJ583376
			HLC-12319	TZAIB302-06	FJ583377
		Hippocampus kuda	HLC-12089	TZAIB166-06	FJ583550
			HLC-11773	TZAIC711-06	FJ583551
			HLC-12284	TZAIC925-06	FJ583552
			HLC-12283	TZAIC924-06	FJ583553
Order Tetraodontiformes					
	Family Balistidae	*Balistapus undulatus*	HLC-12320	TZAIB303-06	FJ582893
		Balistes capriscus	HLC-15135	TZAIB817-07	FJ582894
		Balistoides conspicillum	HLC-11974	TZAIB239-06	FJ582895
			HLC-11571	TZAIC503-06	FJ582896
			HLC-11145	TZAIC437-05	FJ582897
			HLC-11040	TZAIC332-05	FJ582898
		Melichthys indicus	HLC-11717	TZAIC649-06	FJ583651
			HLC-11159	TZAIC451-05	FJ583652
			HLC-11135	TZAIC427-05	FJ583653
		Melichthys niger	HLC-11170	TZAIC462-05	FJ583654
			HLC-13295	TZAIB672-06	FJ583655
			HLC-15217	TZAIB887-07	FJ583656
			HLC-11549	TZAIC481-06	FJ583657
			HLC-10982	TZAIC282-05	FJ583658
		Odonus niger	HLC-11572	TZAIC504-06	FJ583744
		Pseudobalistes flavimarginatus	HLC-11114	TZAIC406-05	FJ583961
		Pseudobalistes fuscus	HLC-12315	TZAIB298-06	FJ583962
			HLC-12033	TZAIB110-06	FJ583963
		Rhinecanthus aculeatus	HLC-12206	TZAIC847-06	FJ584054
			HLC-TSZ-021	TZAIC021-05	FJ584055
			HLC-TSZ-020	TZAIC020-05	FJ584056
			HLC-TSZ-019	TZAIC019-05	FJ584057
			HLC-TSZ-017	TZAIC017-05	FJ584058
			HLC-TSZ-018	TZAIC018-05	FJ584059
		Rhinecanthus rectangulus	HLC-15170	TZAIB852-07	FJ584060
		Rhinecanthus verrucosus	HLC-10722	TZAIC022-05	FJ584061
			HLC-10723	TZAIC023-05	FJ584062
			HLC-10724	TZAIC024-05	FJ584063
			HLC-10726	TZAIC026-05	FJ584064
			HLC-10725	TZAIC025-05	FJ584065
		Sufflamen bursa	HLC-12387	TZAIB370-06	FJ584128
			HLC-12388	TZAIB371-06	FJ584129
			HLC-13047	TZAIB424-06	FJ584130
		Sufflamen chrysopterum	HLC-12325	TZAIB308-06	FJ584131
			HLC-15184	TZAIB854-07	FJ584132
		Xanthichthys auromarginatus	HLC-12268	TZAIC909-06	FJ584243
			HLC-11609	TZAIC541-06	FJ584244
			HLC-10727	TZAIC027-05	FJ584245
			HLC-11608	TZAIC540-06	FJ584246

		HLC-11607	TZAIC539-06	FJ584247
		HLC-11606	TZAIC538-06	FJ584248
	Xanthichthys lineopunctatus	HLC-13041	TZAIB418-06	FJ584249
Family Diodontidae	*Chilomycterus antillarum*	HLC-11864	TZAIC802-06	FJ583135
		HLC-11863	TZAIC801-06	FJ583136
		HLC-11862	TZAIC800-06	FJ583137
		HLC-11861	TZAIC799-06	FJ583138
		HLC-11860	TZAIC798-06	FJ583139
	Diodon holocanthus	HLC-12023	TZAIB100-06	FJ583355
		HLC-15076	TZAIB682-06	FJ583356
		HLC-15156	TZAIB838-07	FJ583357
		HLC-15164	TZAIB846-07	FJ583358
		HLC-15165	TZAIB847-07	FJ583359
		HLC-11928	TZAIB193-06	FJ583360
		HLC-11929	TZAIB194-06	FJ583361
		HLC-12007	TZAIB272-06	FJ583362
		HLC-12008	TZAIB273-06	FJ583363
		HLC-15000	TZAIB681-06	FJ583364
Family Monacanthidae	*Cantherhines pullus*	HLC-15023	TZAIB762-06	FJ582914
		HLC-15024	TZAIB763-06	FJ582915
		HLC-15025	TZAIB764-06	FJ582916
		HLC-15026	TZAIB765-06	FJ582917
		HLC-15027	TZAIB766-06	FJ582918
	Oxymonacanthus longirostris	HLC-13235	TZAIB612-06	FJ583796
		HLC-13236	TZAIB613-06	FJ583797
		HLC-13237	TZAIB614-06	FJ583798
		HLC-11036	TZAIC328-05	FJ583799
		HLC-11035	TZAIC327-05	FJ583800
	Pervagor aspricaudus	HLC-13189	TZAIB566-06	FJ583843
		HLC-15186	TZAIB856-07	FJ583844
		HLC-11676	TZAIC608-06	FJ583845
	Pervagor melanocephalus	HLC-15222	TZAIB892-07	FJ583846
		HLC-15223	TZAIB893-07	FJ583847
		HLC-11567	TZAIC499-06	FJ583848
		HLC-11060	TZAIC352-05	FJ583849
	Pervagor nigrolineatus	HLC-13048	TZAIB425-06	FJ583850
		HLC-13049	TZAIB426-06	FJ583851
Family Ostraciidae	*Acanthostracion quadricornis*	HLC-12116	TZAIB005-06	FJ582647
		HLC-12117	TZAIB006-06	FJ582648
		HLC-12118	TZAIB007-06	FJ582649
		HLC-12119	TZAIB008-06	FJ582650
		HLC-12120	TZAIB009-06	FJ582651
	Lactophrys bicaudalis	HLC-12368	TZAIB351-06	FJ583605
	Lactoria cornuta	HLC-TSZ-004	TZAIC004-05	FJ583606
		HLC-13230	TZAIB607-06	FJ583607
		HLC-13231	TZAIB608-06	FJ583608
		HLC-13232	TZAIB609-06	FJ583609
		HLC-13233	TZAIB610-06	FJ583610
		HLC-13234	TZAIB611-06	FJ583611
		HLC-11813	TZAIC751-06	FJ583612
		HLC-11812	TZAIC750-06	FJ583613
		HLC-11811	TZAIC749-06	FJ583614
		HLC-11793	TZAIC731-06	FJ583615
		HLC-11131	TZAIC423-05	FJ583616
		HLC-TSZ-005	TZAIC005-05	FJ583617
		HLC-10984	TZAIC464-05	FJ583618
		HLC-TSZ-008	TZAIC008-05	FJ583619
		HLC-TSZ-007	TZAIC007-05	FJ583620
		HLC-TSZ-006	TZAIC006-05	FJ583621
	Ostracion sp.	HLC-11576	TZAIC508-06	FJ583770
	Ostracion cubicus	HLC-15067	TZAIB767-06	FJ583771
		HLC-15068	TZAIB768-06	FJ583772
		HLC-15069	TZAIB769-06	FJ583773
		HLC-15070	TZAIB770-06	FJ583774
		HLC-15071	TZAIB771-06	FJ583775
		HLC-11575	TZAIC507-06	FJ583776
	Ostracion meleagris	HLC-13149	TZAIB526-06	FJ583777
		HLC-11839	TZAIC777-06	FJ583778
		HLC-12354	TZAIB337-06	FJ583779
		HLC-12355	TZAIB338-06	FJ583780
		HLC-12356	TZAIB339-06	FJ583781
		HLC-12357	TZAIB340-06	FJ583782
		HLC-15161	TZAIB843-07	FJ583783
		HLC-15162	TZAIB844-07	FJ583784
		HLC-15163	TZAIB845-07	FJ583785
		HLC-11730	TZAIC668-06	FJ583786
	Ostracion nasus	HLC-12205	TZAIB094-06	FJ583787
		HLC-11858	TZAIC796-06	FJ583788
		HLC-12207	TZAIC848-06	FJ583789

				Ostracion rhinorhynchos	HLC-11842	TZAIC780-06	FJ583790
				Tetrosomus gibbosus	HLC-15110	TZAIB793-07	FJ584171
			Family Tetraodontidae	*Arothron hispidus*	HLC-13172	TZAIB549-06	FJ582875
				Arothron meleagris	HLC-13069	TZAIB446-06	FJ582876
				Arothron nigropunctatus	HLC-12416	TZAIB399-06	FJ582877
					HLC-12415	TZAIB398-06	FJ582878
					HLC-11869	TZAIC807-06	FJ582879
					HLC-12417	TZAIB400-06	FJ582880
					HLC-11814	TZAIC752-06	FJ582881
					HLC-11868	TZAIC806-06	FJ582882
					HLC-11867	TZAIC805-06	FJ582883
					HLC-11866	TZAIC804-06	FJ582884
					HLC-11865	TZAIC803-06	FJ582885
					HLC-11765	TZAIC703-06	FJ582886
					HLC-11130	TZAIC422-05	FJ582887
					HLC-11129	TZAIC421-05	FJ582888
					HLC-11094	TZAIC386-05	FJ582889
					HLC-11095	TZAIC387-05	FJ582890
				Canthigaster amboinensis	HLC-15204	TZAIB874-07	FJ582919
				Canthigaster coronata	HLC-12132	TZAIB021-06	FJ582920
					HLC-12133	TZAIB022-06	FJ582921
				Canthigaster solandri	HLC-11787	TZAIC725-06	FJ582922
					HLC-11021	TZAIC313-05	FJ582923
					HLC-11020	TZAIC312-05	FJ582924
				Canthigaster valentini	HLC-12124	TZAIB013-06	FJ582925
					HLC-11642	TZAIC574-06	FJ582926
	Superorder Clupeomorpha						
		Order Anguilliformes					
			Family Muraenidae	*Echidna nebulosa*	HLC-13031	TZAIB408-06	FJ583379
					HLC-11769	TZAIC707-06	FJ583380
				Enchelycore pardalis	HLC-15183	TZAIB853-07	FJ583395
				Rhinomuraena quaesita	HLC-12186	TZAIB075-06	FJ584066
					HLC-13109	TZAIB486-06	FJ584067
					HLC-13110	TZAIB487-06	FJ584068
					HLC-15093	TZAIB776-07	FJ584069
					HLC-15094	TZAIB777-07	FJ584070
	Superorder Ostariophysi						
		Order Siluriformes					
			Family Plotosidae	*Plotosus lineatus*	HLC-11046	TZAIC338-05	FJ583870
					HLC-11047	TZAIC339-05	FJ583871
					HLC-11049	TZAIC341-05	FJ583872
					HLC-11048	TZAIC340-05	FJ583873
					HLC-11050	TZAIC342-05	FJ583874
	Superorder Paracanthopterygii						
		Order Batrachoidiformes					
			Family Batrachoididae	*Opsanus beta*	HLC-10983	TZAIC463-05	FJ583769
		Order Lophiiformes					
			Family Antennariidae	*Antennarius commerson*	HLC-11029	TZAIC321-05	FJ582854
				Antennarius hispidus	HLC-15088	TZAIB690-06	FJ582855
					HLC-15095	TZAIB778-07	FJ582856
				Antennarius nummifer	HLC-13028	TZAIB405-06	FJ582857
				Antennarius pictus	HLC-13238	TZAIB615-06	FJ582858
				Antennarius sp.	HLC-15028	TZAIB689-06	FJ582859
				Histrio histrio	HLC-15035	TZAIB678-06	FJ583554
					HLC-15077	TZAIB679-06	FJ583555
					HLC-13217	TZAIB594-06	FJ583556
					HLC-13218	TZAIB595-06	FJ583557
		Order Ophidiiformes					
			Family Bythitidae	*Brotulina fusca*	HLC-12092	TZAIB169-06	FJ582908
			Family Ophidiidae	*Lepophidium brevibarbe*	HLC-12197	TZAIB086-06	FJ583622
					HLC-12097	TZAIB174-06	FJ583623
					HLC-12200	TZAIB089-06	FJ583624
					HLC-12198	TZAIB087-06	FJ583625
					HLC-12199	TZAIB088-06	FJ583626
Class Elasmobranchii							
	Superorder Batoidea						
		Order Rajiformes					
			Family Dasyatidae	*Neotrygon kuhlii*	HLC-12299	TZAIC940-06	FJ583339
					HLC-12121	TZAIB010-06	FJ583340
					HLC-12298	TZAIC939-06	FJ583341
				Taeniura lymma	HLC-12392	TZAIB375-06	FJ584168
					HLC-12410	TZAIB393-06	FJ584169
					HLC-12409	TZAIB392-06	FJ584170
		Order Torpediniformes					
			Family Narcinidae	*Narcine brunnea*	HLC-12282	TZAIC923-06	FJ583678
	Superorder Selachimorpha						
		Order Orectolobiformes					
			Family Hemiscylliidae	*Chiloscyllium griseum*	HLC-12412	TZAIB395-06	FJ583140
					HLC-12411	TZAIB394-06	FJ583141

Chiloscyllium punctatum	HLC-13259	TZAIB636-06	FJ583142
	HLC-13181	TZAIB558-06	FJ583143
	HLC-15206	TZAIB876-07	FJ583144
