# Supplementary material for: Barcoding Nemo: DNA-Based Identifications for the Ornamental Fish Trade
Source: PLoS One. 2009 Jul 21;4(7):e6300. doi: 10.1371/journal.pone.0006300 (PMC2708913; doi:10.1371/journal.pone.0006300)
Supplement: Figure S1 — A neighbour-joining tree of COI sequence divergences (K2P) in all 1638 individuals of this study. Species names, BOLD process ID, Sample ID, sequence length, and numbers of ambiguous bases are given at branch tips. (1.59 MB PDF) [file pone.0006300.s002.pdf]

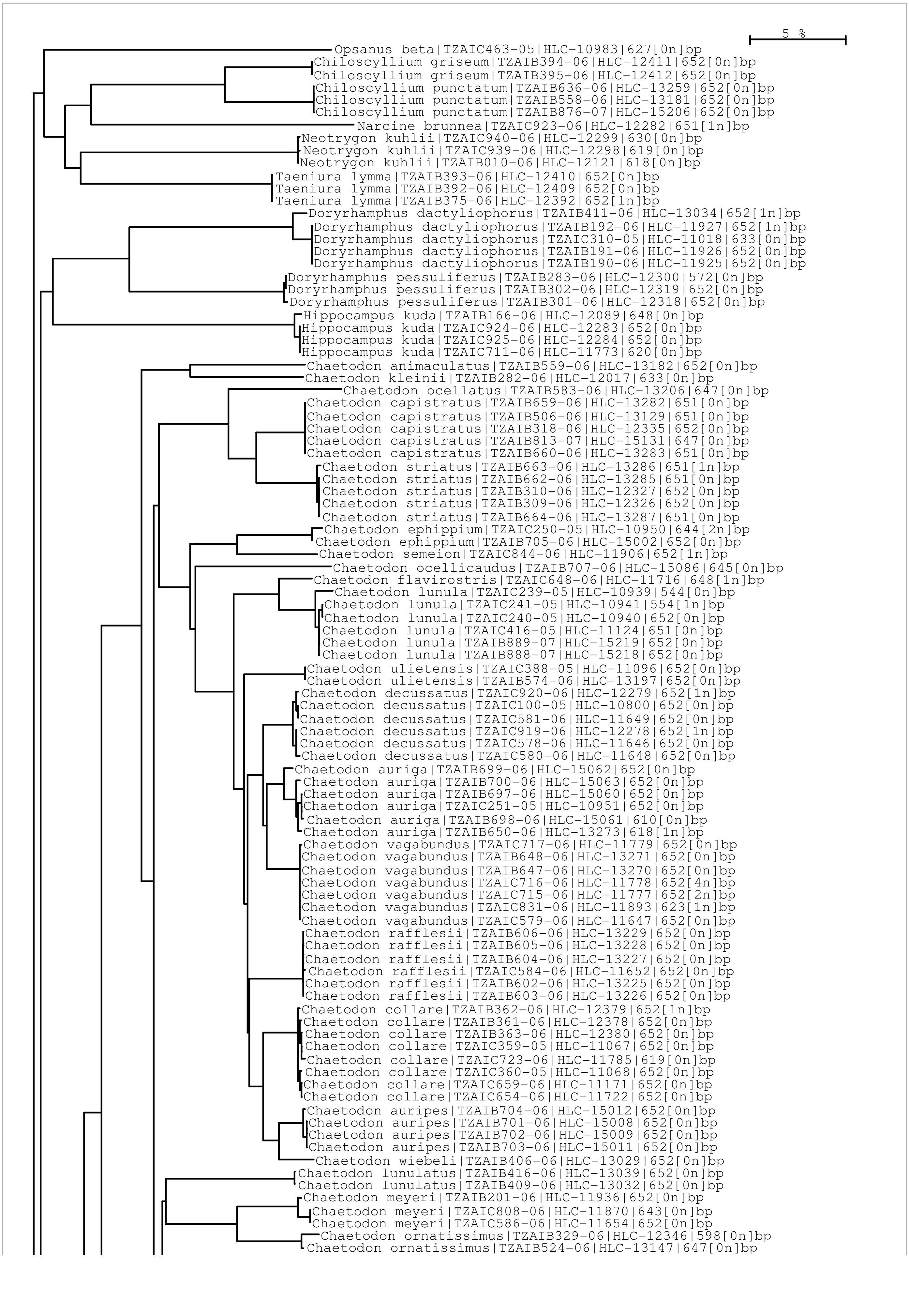
5 %
Opsanus beta|TZAIC463-05|HLC-10983|627[0n]bp
Chiloscyllium griseum|TZAIB394-06|HLC-12411|652[0n]bp
Chiloscyllium griseum|TZAIB395-06|HLC-12412|652[0n]bp
Chiloscyllium punctatum|TZAIB636-06|HLC-13259|652[0n]bp
Chiloscyllium punctatum|TZAIB558-06|HLC-13181|652[0n]bp
Chiloscyllium punctatum|TZAIB876-07|HLC-15206|652[0n]bp
Narcine brunnea|TZAIC923-06|HLC-12282|651[1n]bp
Neotrygon kuhlii|TZAIC940-06|HLC-12299|630[0n]bp
Neotrygon kuhlii|TZAIC939-06|HLC-12298|619[0n]bp
Neotrygon kuhlii|TZAIB010-06|HLC-12121|618[0n]bp
Taeniura lymma|TZAIB393-06|HLC-12410|652[0n]bp
Taeniura lymma|TZAIB392-06|HLC-12409|652[0n]bp
Taeniura lymma|TZAIB375-06|HLC-12392|652[1n]bp
Doryrhamphus dactyliophorus|TZAIB411-06|HLC-13034|652[1n]bp
Doryrhamphus dactyliophorus|TZAIB192-06|HLC-11927|652[1n]bp
Doryrhamphus dactyliophorus|TZAIC310-05|HLC-11018|633[0n]bp
Doryrhamphus dactyliophorus|TZAIB191-06|HLC-11926|652[0n]bp
Doryrhamphus dactyliophorus|TZAIB190-06|HLC-11925|652[0n]bp
Doryrhamphus pessuliferus|TZAIB283-06|HLC-12300|572[0n]bp
Doryrhamphus pessuliferus|TZAIB302-06|HLC-12319|652[0n]bp
Doryrhamphus pessuliferus|TZAIB301-06|HLC-12318|652[0n]bp
Hippocampus kuda|TZAIB166-06|HLC-12089|648[0n]bp
Hippocampus kuda|TZAIC924-06|HLC-12283|652[0n]bp
Hippocampus kuda|TZAIC925-06|HLC-12284|652[0n]bp
Hippocampus kuda|TZAIC711-06|HLC-11773|620[0n]bp
Chaetodon animaculatus|TZAIB559-06|HLC-13182|652[0n]bp
Chaetodon kleinii|TZAIB282-06|HLC-12017|633[0n]bp
Chaetodon ocellatus|TZAIB583-06|HLC-13206|647[0n]bp
Chaetodon capistratus|TZAIB659-06|HLC-13282|651[0n]bp
Chaetodon capistratus|TZAIB506-06|HLC-13129|651[0n]bp
Chaetodon capistratus|TZAIB318-06|HLC-12335|652[0n]bp
Chaetodon capistratus|TZAIB813-07|HLC-15131|647[0n]bp
Chaetodon capistratus|TZAIB660-06|HLC-13283|651[0n]bp
Chaetodon striatus|TZAIB663-06|HLC-13286|651[1n]bp
Chaetodon striatus|TZAIB662-06|HLC-13285|651[0n]bp
Chaetodon striatus|TZAIB310-06|HLC-12327|652[0n]bp
Chaetodon striatus|TZAIB309-06|HLC-12326|652[0n]bp
Chaetodon striatus|TZAIB664-06|HLC-13287|651[0n]bp
Chaetodon ephippium|TZAIC250-05|HLC-10950|644[2n]bp
Chaetodon ephippium|TZAIB705-06|HLC-15002|652[0n]bp
Chaetodon semeion|TZAIC844-06|HLC-11906|652[1n]bp
Chaetodon ocellicaudus|TZAIB707-06|HLC-15086|645[0n]bp
Chaetodon flavirostris|TZAIC648-06|HLC-11716|648[1n]bp
Chaetodon lunula|TZAIC239-05|HLC-10939|544[0n]bp
Chaetodon lunula|TZAIC241-05|HLC-10941|554[1n]bp
Chaetodon lunula|TZAIC240-05|HLC-10940|652[0n]bp
Chaetodon lunula|TZAIC416-05|HLC-11124|651[0n]bp
Chaetodon lunula|TZAIB889-07|HLC-15219|652[0n]bp
Chaetodon lunula|TZAIB888-07|HLC-15218|652[0n]bp
Chaetodon ulietensis|TZAIC388-05|HLC-11096|652[0n]bp
Chaetodon ulietensis|TZAIB574-06|HLC-13197|652[0n]bp
Chaetodon decussatus|TZAIC920-06|HLC-12279|652[1n]bp
Chaetodon decussatus|TZAIC100-05|HLC-10800|652[0n]bp
Chaetodon decussatus|TZAIC581-06|HLC-11649|652[0n]bp
Chaetodon decussatus|TZAIC919-06|HLC-12278|652[1n]bp
Chaetodon decussatus|TZAIC578-06|HLC-11646|652[0n]bp
Chaetodon decussatus|TZAIC580-06|HLC-11648|652[0n]bp
Chaetodon auriga|TZAIB699-06|HLC-15062|652[0n]bp
Chaetodon auriga|TZAIB700-06|HLC-15063|652[0n]bp
Chaetodon auriga|TZAIB697-06|HLC-15060|652[0n]bp
Chaetodon auriga|TZAIC251-05|HLC-10951|652[0n]bp
Chaetodon auriga|TZAIB698-06|HLC-15061|610[0n]bp
Chaetodon auriga|TZAIB650-06|HLC-13273|618[1n]bp
Chaetodon vagabundus|TZAIC717-06|HLC-11779|652[0n]bp
Chaetodon vagabundus|TZAIB648-06|HLC-13271|652[0n]bp
Chaetodon vagabundus|TZAIB647-06|HLC-13270|652[0n]bp
Chaetodon vagabundus|TZAIC716-06|HLC-11778|652[4n]bp
Chaetodon vagabundus|TZAIC715-06|HLC-11777|652[2n]bp
Chaetodon vagabundus|TZAIC831-06|HLC-11893|623[1n]bp
Chaetodon vagabundus|TZAIC579-06|HLC-11647|652[0n]bp
Chaetodon rafflesii|TZAIB606-06|HLC-13229|652[0n]bp
Chaetodon rafflesii|TZAIB605-06|HLC-13228|652[0n]bp
Chaetodon rafflesii|TZAIB604-06|HLC-13227|652[0n]bp
Chaetodon rafflesii|TZAIC584-06|HLC-11652|652[0n]bp
Chaetodon rafflesii|TZAIB602-06|HLC-13225|652[0n]bp
Chaetodon rafflesii|TZAIB603-06|HLC-13226|652[0n]bp
Chaetodon collare|TZAIB362-06|HLC-12379|652[1n]bp
Chaetodon collare|TZAIB361-06|HLC-12378|652[0n]bp
Chaetodon collare|TZAIB363-06|HLC-12380|652[0n]bp
Chaetodon collare|TZAIC359-05|HLC-11067|652[0n]bp
Chaetodon collare|TZAIC723-06|HLC-11785|619[0n]bp
Chaetodon collare|TZAIC360-05|HLC-11068|652[0n]bp
Chaetodon collare|TZAIC659-06|HLC-11171|652[0n]bp
Chaetodon collare|TZAIC654-06|HLC-11722|652[0n]bp
Chaetodon auripes|TZAIB704-06|HLC-15012|652[0n]bp
Chaetodon auripes|TZAIB701-06|HLC-15008|652[0n]bp
Chaetodon auripes|TZAIB702-06|HLC-15009|652[0n]bp
Chaetodon auripes|TZAIB703-06|HLC-15011|652[0n]bp
Chaetodon wiebeli|TZAIB406-06|HLC-13029|652[0n]bp
Chaetodon lunulatus|TZAIB416-06|HLC-13039|652[0n]bp
Chaetodon lunulatus|TZAIB409-06|HLC-13032|652[0n]bp
Chaetodon meyeri|TZAIB201-06|HLC-11936|652[0n]bp
Chaetodon meyeri|TZAIC808-06|HLC-11870|643[0n]bp
Chaetodon meyeri|TZAIC586-06|HLC-11654|652[0n]bp
Chaetodon ornatissimus|TZAIB329-06|HLC-12346|598[0n]bp
Chaetodon ornatissimus|TZAIB524-06|HLC-13147|647[0n]bp

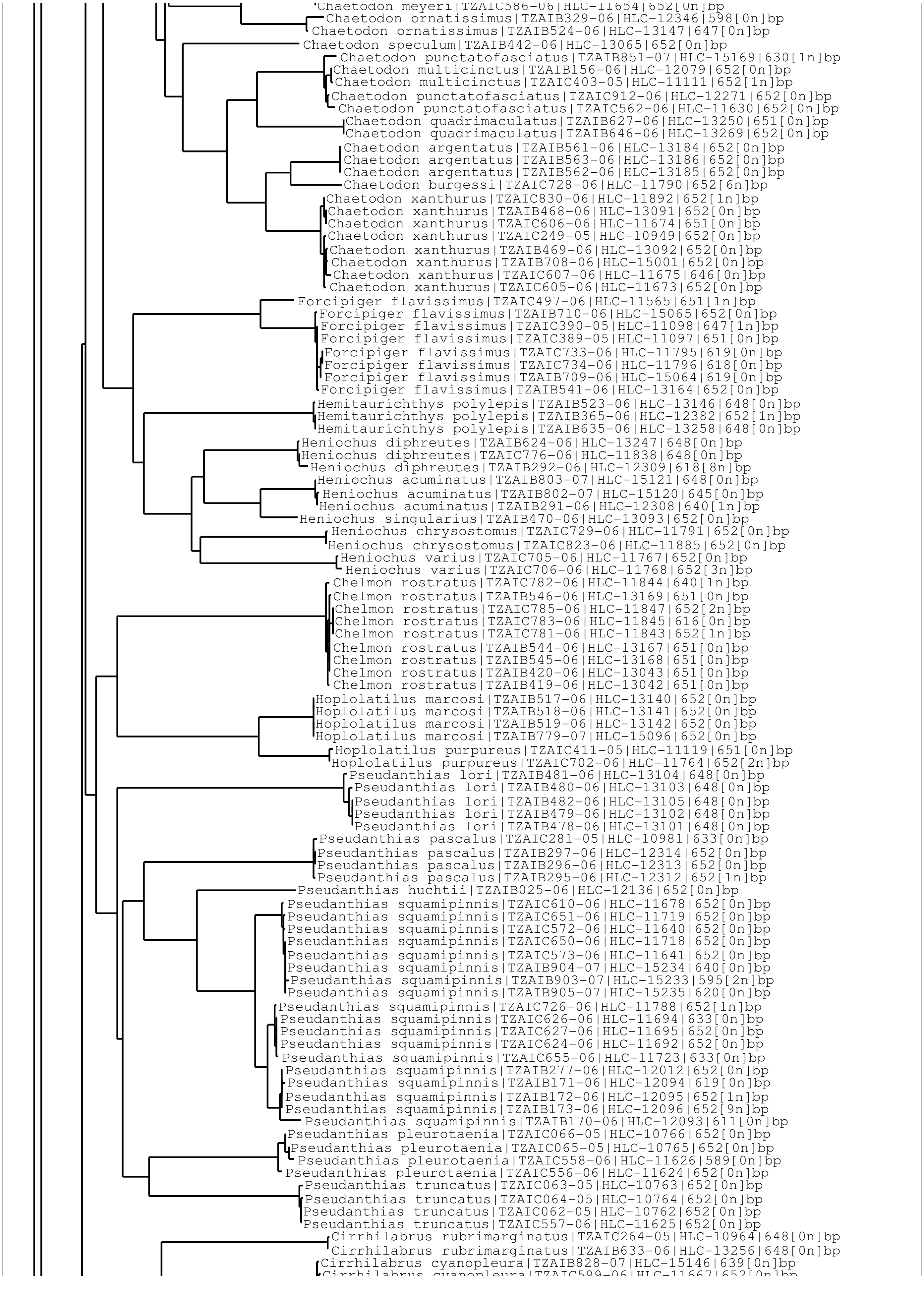
Chaetodon meyeri|TZAIC586-06|HLC-11654|652[0n]bp
Chaetodon ornatissimus|TZAIB329-06|HLC-12346|598[0n]bp
Chaetodon ornatissimus|TZAIB524-06|HLC-13147|647[0n]bp
Chaetodon speculum|TZAIB442-06|HLC-13065|652[0n]bp
Chaetodon punctatofasciatus|TZAIB851-07|HLC-15169|630[1n]bp
Chaetodon multicinctus|TZAIB156-06|HLC-12079|652[0n]bp
Chaetodon multicinctus|TZAIC403-05|HLC-11111|652[1n]bp
Chaetodon punctatofasciatus|TZAIC912-06|HLC-12271|652[0n]bp
Chaetodon punctatofasciatus|TZAIC562-06|HLC-11630|652[0n]bp
Chaetodon quadrimaculatus|TZAIB627-06|HLC-13250|651[0n]bp
Chaetodon quadrimaculatus|TZAIB646-06|HLC-13269|652[0n]bp
Chaetodon argentatus|TZAIB561-06|HLC-13184|652[0n]bp
Chaetodon argentatus|TZAIB563-06|HLC-13186|652[0n]bp
Chaetodon argentatus|TZAIB562-06|HLC-13185|652[0n]bp
Chaetodon burgessi|TZAIC728-06|HLC-11790|652[6n]bp
Chaetodon xanthurus|TZAIC830-06|HLC-11892|652[1n]bp
Chaetodon xanthurus|TZAIB468-06|HLC-13091|652[0n]bp
Chaetodon xanthurus|TZAIC606-06|HLC-11674|651[0n]bp
Chaetodon xanthurus|TZAIC249-05|HLC-10949|652[0n]bp
Chaetodon xanthurus|TZAIB469-06|HLC-13092|652[0n]bp
Chaetodon xanthurus|TZAIB708-06|HLC-15001|652[0n]bp
Chaetodon xanthurus|TZAIC607-06|HLC-11675|646[0n]bp
Chaetodon xanthurus|TZAIC605-06|HLC-11673|652[0n]bp
Forcipiger flavissimus|TZAIC497-06|HLC-11565|651[1n]bp
Forcipiger flavissimus|TZAIB710-06|HLC-15065|652[0n]bp
Forcipiger flavissimus|TZAIC390-05|HLC-11098|647[1n]bp
Forcipiger flavissimus|TZAIC389-05|HLC-11097|651[0n]bp
Forcipiger flavissimus|TZAIC733-06|HLC-11795|619[0n]bp
Forcipiger flavissimus|TZAIC734-06|HLC-11796|618[0n]bp
Forcipiger flavissimus|TZAIB709-06|HLC-15064|619[0n]bp
Forcipiger flavissimus|TZAIB541-06|HLC-13164|652[0n]bp
Hemitaurichthys polylepis|TZAIB523-06|HLC-13146|648[0n]bp
Hemitaurichthys polylepis|TZAIB365-06|HLC-12382|652[1n]bp
Hemitaurichthys polylepis|TZAIB635-06|HLC-13258|648[0n]bp
Heniochus diphreutes|TZAIB624-06|HLC-13247|648[0n]bp
Heniochus diphreutes|TZAIC776-06|HLC-11838|648[0n]bp
Heniochus diphreutes|TZAIB292-06|HLC-12309|618[8n]bp
Heniochus acuminatus|TZAIB803-07|HLC-15121|648[0n]bp
Heniochus acuminatus|TZAIB802-07|HLC-15120|645[0n]bp
Heniochus acuminatus|TZAIB291-06|HLC-12308|640[1n]bp
Heniochus singularius|TZAIB470-06|HLC-13093|652[0n]bp
Heniochus chrysostomus|TZAIC729-06|HLC-11791|652[0n]bp
Heniochus chrysostomus|TZAIC823-06|HLC-11885|652[0n]bp
Heniochus varius|TZAIC705-06|HLC-11767|652[0n]bp
Heniochus varius|TZAIC706-06|HLC-11768|652[3n]bp
Chelmon rostratus|TZAIC782-06|HLC-11844|640[1n]bp
Chelmon rostratus|TZAIB546-06|HLC-13169|651[0n]bp
Chelmon rostratus|TZAIC785-06|HLC-11847|652[2n]bp
Chelmon rostratus|TZAIC783-06|HLC-11845|616[0n]bp
Chelmon rostratus|TZAIC781-06|HLC-11843|652[1n]bp
Chelmon rostratus|TZAIB544-06|HLC-13167|651[0n]bp
Chelmon rostratus|TZAIB545-06|HLC-13168|651[0n]bp
Chelmon rostratus|TZAIB420-06|HLC-13043|651[0n]bp
Chelmon rostratus|TZAIB419-06|HLC-13042|651[0n]bp
Hoplolatilus marcosi|TZAIB517-06|HLC-13140|652[0n]bp
Hoplolatilus marcosi|TZAIB518-06|HLC-13141|652[0n]bp
Hoplolatilus marcosi|TZAIB519-06|HLC-13142|652[0n]bp
Hoplolatilus marcosi|TZAIB779-07|HLC-15096|652[0n]bp
Hoplolatilus purpureus|TZAIC411-05|HLC-11119|651[0n]bp
Hoplolatilus purpureus|TZAIC702-06|HLC-11764|652[2n]bp
Pseudanthias lori|TZAIB481-06|HLC-13104|648[0n]bp
Pseudanthias lori|TZAIB480-06|HLC-13103|648[0n]bp
Pseudanthias lori|TZAIB482-06|HLC-13105|648[0n]bp
Pseudanthias lori|TZAIB479-06|HLC-13102|648[0n]bp
Pseudanthias lori|TZAIB478-06|HLC-13101|648[0n]bp
Pseudanthias pascalus|TZAIC281-05|HLC-10981|633[0n]bp
Pseudanthias pascalus|TZAIB297-06|HLC-12314|652[0n]bp
Pseudanthias pascalus|TZAIB296-06|HLC-12313|652[0n]bp
Pseudanthias pascalus|TZAIB295-06|HLC-12312|652[1n]bp
Pseudanthias huchtii|TZAIB025-06|HLC-12136|652[0n]bp
Pseudanthias squamipinnis|TZAIC610-06|HLC-11678|652[0n]bp
Pseudanthias squamipinnis|TZAIC651-06|HLC-11719|652[0n]bp
Pseudanthias squamipinnis|TZAIC572-06|HLC-11640|652[0n]bp
Pseudanthias squamipinnis|TZAIC650-06|HLC-11718|652[0n]bp
Pseudanthias squamipinnis|TZAIC573-06|HLC-11641|652[0n]bp
Pseudanthias squamipinnis|TZAIB904-07|HLC-15234|640[0n]bp
Pseudanthias squamipinnis|TZAIB903-07|HLC-15233|595[2n]bp
Pseudanthias squamipinnis|TZAIB905-07|HLC-15235|620[0n]bp
Pseudanthias squamipinnis|TZAIC726-06|HLC-11788|652[1n]bp
Pseudanthias squamipinnis|TZAIC626-06|HLC-11694|633[0n]bp
Pseudanthias squamipinnis|TZAIC627-06|HLC-11695|652[0n]bp
Pseudanthias squamipinnis|TZAIC624-06|HLC-11692|652[0n]bp
Pseudanthias squamipinnis|TZAIC655-06|HLC-11723|633[0n]bp
Pseudanthias squamipinnis|TZAIB277-06|HLC-12012|652[0n]bp
Pseudanthias squamipinnis|TZAIB171-06|HLC-12094|619[0n]bp
Pseudanthias squamipinnis|TZAIB172-06|HLC-12095|652[1n]bp
Pseudanthias squamipinnis|TZAIB173-06|HLC-12096|652[9n]bp
Pseudanthias squamipinnis|TZAIB170-06|HLC-12093|611[0n]bp
Pseudanthias pleurotaenia|TZAIC066-05|HLC-10766|652[0n]bp
Pseudanthias pleurotaenia|TZAIC065-05|HLC-10765|652[0n]bp
Pseudanthias pleurotaenia|TZAIC558-06|HLC-11626|589[0n]bp
Pseudanthias pleurotaenia|TZAIC556-06|HLC-11624|652[0n]bp
Pseudanthias truncatus|TZAIC063-05|HLC-10763|652[0n]bp
Pseudanthias truncatus|TZAIC064-05|HLC-10764|652[0n]bp
Pseudanthias truncatus|TZAIC062-05|HLC-10762|652[0n]bp
Pseudanthias truncatus|TZAIC557-06|HLC-11625|652[0n]bp
Cirrhilabrus rubrimarginatus|TZAIC264-05|HLC-10964|648[0n]bp
Cirrhilabrus rubrimarginatus|TZAIB633-06|HLC-13256|648[0n]bp
Cirrhilabrus cyanopleura|TZAIB828-07|HLC-15146|639[0n]bp
Cirrhilabrus cyanopleura|TZAIC599-06|HLC-11667|652[0n]bp

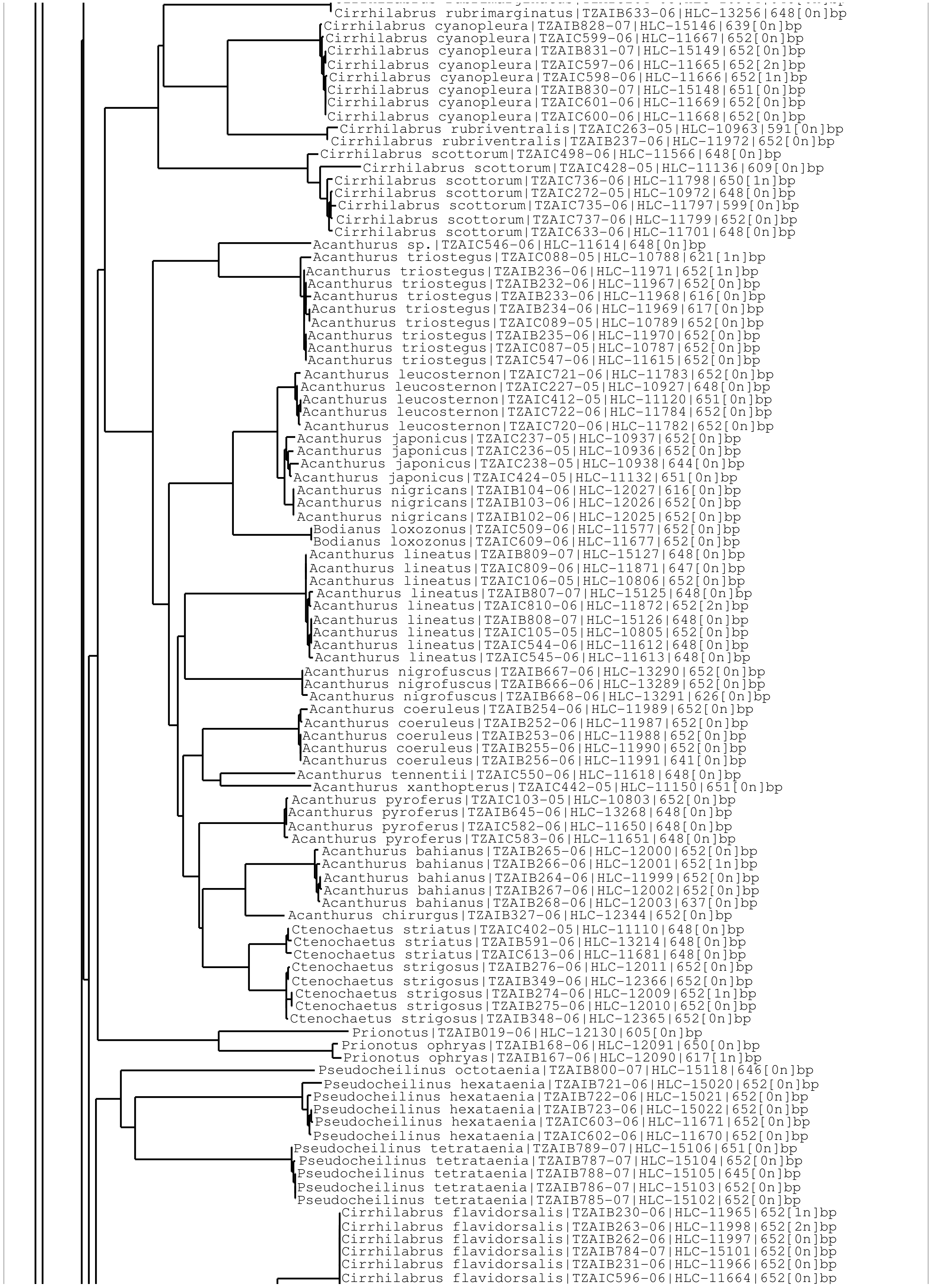

Cirrhilabrus rubrimarginatus|TZAIB633-06|HLC-13256|648[0n]bp
Cirrhilabrus cyanopleura|TZAIB828-07|HLC-15146|639[0n]bp
Cirrhilabrus cyanopleura|TZAIC599-06|HLC-11667|652[0n]bp
Cirrhilabrus cyanopleura|TZAIB831-07|HLC-15149|652[0n]bp
Cirrhilabrus cyanopleura|TZAIC597-06|HLC-11665|652[2n]bp
Cirrhilabrus cyanopleura|TZAIC598-06|HLC-11666|652[1n]bp
Cirrhilabrus cyanopleura|TZAIB830-07|HLC-15148|651[0n]bp
Cirrhilabrus cyanopleura|TZAIC601-06|HLC-11669|652[0n]bp
Cirrhilabrus cyanopleura|TZAIC600-06|HLC-11668|652[0n]bp
Cirrhilabrus rubriventralis|TZAIC263-05|HLC-10963|591[0n]bp
Cirrhilabrus rubriventralis|TZAIB237-06|HLC-11972|652[0n]bp
Cirrhilabrus scottorum|TZAIC498-06|HLC-11566|648[0n]bp
Cirrhilabrus scottorum|TZAIC428-05|HLC-11136|609[0n]bp
Cirrhilabrus scottorum|TZAIC736-06|HLC-11798|650[1n]bp
Cirrhilabrus scottorum|TZAIC272-05|HLC-10972|648[0n]bp
Cirrhilabrus scottorum|TZAIC735-06|HLC-11797|599[0n]bp
Cirrhilabrus scottorum|TZAIC737-06|HLC-11799|652[0n]bp
Cirrhilabrus scottorum|TZAIC633-06|HLC-11701|648[0n]bp
Acanthurus sp.|TZAIC546-06|HLC-11614|648[0n]bp
Acanthurus triostegus|TZAIC088-05|HLC-10788|621[1n]bp
Acanthurus triostegus|TZAIB236-06|HLC-11971|652[1n]bp
Acanthurus triostegus|TZAIB232-06|HLC-11967|652[0n]bp
Acanthurus triostegus|TZAIB233-06|HLC-11968|616[0n]bp
Acanthurus triostegus|TZAIB234-06|HLC-11969|617[0n]bp
Acanthurus triostegus|TZAIC089-05|HLC-10789|652[0n]bp
Acanthurus triostegus|TZAIB235-06|HLC-11970|652[0n]bp
Acanthurus triostegus|TZAIC087-05|HLC-10787|652[0n]bp
Acanthurus triostegus|TZAIC547-06|HLC-11615|652[0n]bp
Acanthurus leucosternon|TZAIC721-06|HLC-11783|652[0n]bp
Acanthurus leucosternon|TZAIC227-05|HLC-10927|648[0n]bp
Acanthurus leucosternon|TZAIC412-05|HLC-11120|651[0n]bp
Acanthurus leucosternon|TZAIC722-06|HLC-11784|652[0n]bp
Acanthurus leucosternon|TZAIC720-06|HLC-11782|652[0n]bp
Acanthurus japonicus|TZAIC237-05|HLC-10937|652[0n]bp
Acanthurus japonicus|TZAIC236-05|HLC-10936|652[0n]bp
Acanthurus japonicus|TZAIC238-05|HLC-10938|644[0n]bp
Acanthurus japonicus|TZAIC424-05|HLC-11132|651[0n]bp
Acanthurus nigricans|TZAIB104-06|HLC-12027|616[0n]bp
Acanthurus nigricans|TZAIB103-06|HLC-12026|652[0n]bp
Acanthurus nigricans|TZAIB102-06|HLC-12025|652[0n]bp
Bodianus loxozonus|TZAIC509-06|HLC-11577|652[0n]bp
Bodianus loxozonus|TZAIC609-06|HLC-11677|652[0n]bp
Acanthurus lineatus|TZAIB809-07|HLC-15127|648[0n]bp
Acanthurus lineatus|TZAIC809-06|HLC-11871|647[0n]bp
Acanthurus lineatus|TZAIC106-05|HLC-10806|652[0n]bp
Acanthurus lineatus|TZAIB807-07|HLC-15125|648[0n]bp
Acanthurus lineatus|TZAIC810-06|HLC-11872|652[2n]bp
Acanthurus lineatus|TZAIB808-07|HLC-15126|648[0n]bp
Acanthurus lineatus|TZAIC105-05|HLC-10805|652[0n]bp
Acanthurus lineatus|TZAIC544-06|HLC-11612|648[0n]bp
Acanthurus lineatus|TZAIC545-06|HLC-11613|648[0n]bp
Acanthurus nigrofuscus|TZAIB667-06|HLC-13290|652[0n]bp
Acanthurus nigrofuscus|TZAIB666-06|HLC-13289|652[0n]bp
Acanthurus nigrofuscus|TZAIB668-06|HLC-13291|626[0n]bp
Acanthurus coeruleus|TZAIB254-06|HLC-11989|652[0n]bp
Acanthurus coeruleus|TZAIB252-06|HLC-11987|652[0n]bp
Acanthurus coeruleus|TZAIB253-06|HLC-11988|652[0n]bp
Acanthurus coeruleus|TZAIB255-06|HLC-11990|652[0n]bp
Acanthurus coeruleus|TZAIB256-06|HLC-11991|641[0n]bp
Acanthurus tennentii|TZAIC550-06|HLC-11618|648[0n]bp
Acanthurus xanthopterus|TZAIC442-05|HLC-11150|651[0n]bp
Acanthurus pyroferus|TZAIC103-05|HLC-10803|652[0n]bp
Acanthurus pyroferus|TZAIB645-06|HLC-13268|648[0n]bp
Acanthurus pyroferus|TZAIC582-06|HLC-11650|648[0n]bp
Acanthurus pyroferus|TZAIC583-06|HLC-11651|648[0n]bp
Acanthurus bahianus|TZAIB265-06|HLC-12000|652[0n]bp
Acanthurus bahianus|TZAIB266-06|HLC-12001|652[1n]bp
Acanthurus bahianus|TZAIB264-06|HLC-11999|652[0n]bp
Acanthurus bahianus|TZAIB267-06|HLC-12002|652[0n]bp
Acanthurus bahianus|TZAIB268-06|HLC-12003|637[0n]bp
Acanthurus chirurgus|TZAIB327-06|HLC-12344|652[0n]bp
Ctenochaetus striatus|TZAIC402-05|HLC-11110|648[0n]bp
Ctenochaetus striatus|TZAIB591-06|HLC-13214|648[0n]bp
Ctenochaetus striatus|TZAIC613-06|HLC-11681|648[0n]bp
Ctenochaetus strigosus|TZAIB276-06|HLC-12011|652[0n]bp
Ctenochaetus strigosus|TZAIB349-06|HLC-12366|652[0n]bp
Ctenochaetus strigosus|TZAIB274-06|HLC-12009|652[1n]bp
Ctenochaetus strigosus|TZAIB275-06|HLC-12010|652[0n]bp
Ctenochaetus strigosus|TZAIB348-06|HLC-12365|652[0n]bp
Prionotus|TZAIB019-06|HLC-12130|605[0n]bp
Prionotus ophryas|TZAIB168-06|HLC-12091|650[0n]bp
Prionotus ophryas|TZAIB167-06|HLC-12090|617[1n]bp
Pseudocheilinus octotaenia|TZAIB800-07|HLC-15118|646[0n]bp
Pseudocheilinus hexataenia|TZAIB721-06|HLC-15020|652[0n]bp
Pseudocheilinus hexataenia|TZAIB722-06|HLC-15021|652[0n]bp
Pseudocheilinus hexataenia|TZAIB723-06|HLC-15022|652[0n]bp
Pseudocheilinus hexataenia|TZAIC603-06|HLC-11671|652[0n]bp
Pseudocheilinus hexataenia|TZAIC602-06|HLC-11670|652[0n]bp
Pseudocheilinus tetrataenia|TZAIB789-07|HLC-15106|651[0n]bp
Pseudocheilinus tetrataenia|TZAIB787-07|HLC-15104|652[0n]bp
Pseudocheilinus tetrataenia|TZAIB788-07|HLC-15105|645[0n]bp
Pseudocheilinus tetrataenia|TZAIB786-07|HLC-15103|652[0n]bp
Pseudocheilinus tetrataenia|TZAIB785-07|HLC-15102|652[0n]bp
Cirrhilabrus flavidorsalis|TZAIB230-06|HLC-11965|652[1n]bp
Cirrhilabrus flavidorsalis|TZAIB263-06|HLC-11998|652[2n]bp
Cirrhilabrus flavidorsalis|TZAIB262-06|HLC-11997|652[0n]bp
Cirrhilabrus flavidorsalis|TZAIB784-07|HLC-15101|652[0n]bp
Cirrhilabrus flavidorsalis|TZAIB231-06|HLC-11966|652[0n]bp
Cirrhilabrus flavidorsalis|TZAIC596-06|HLC-11664|652[0n]bp

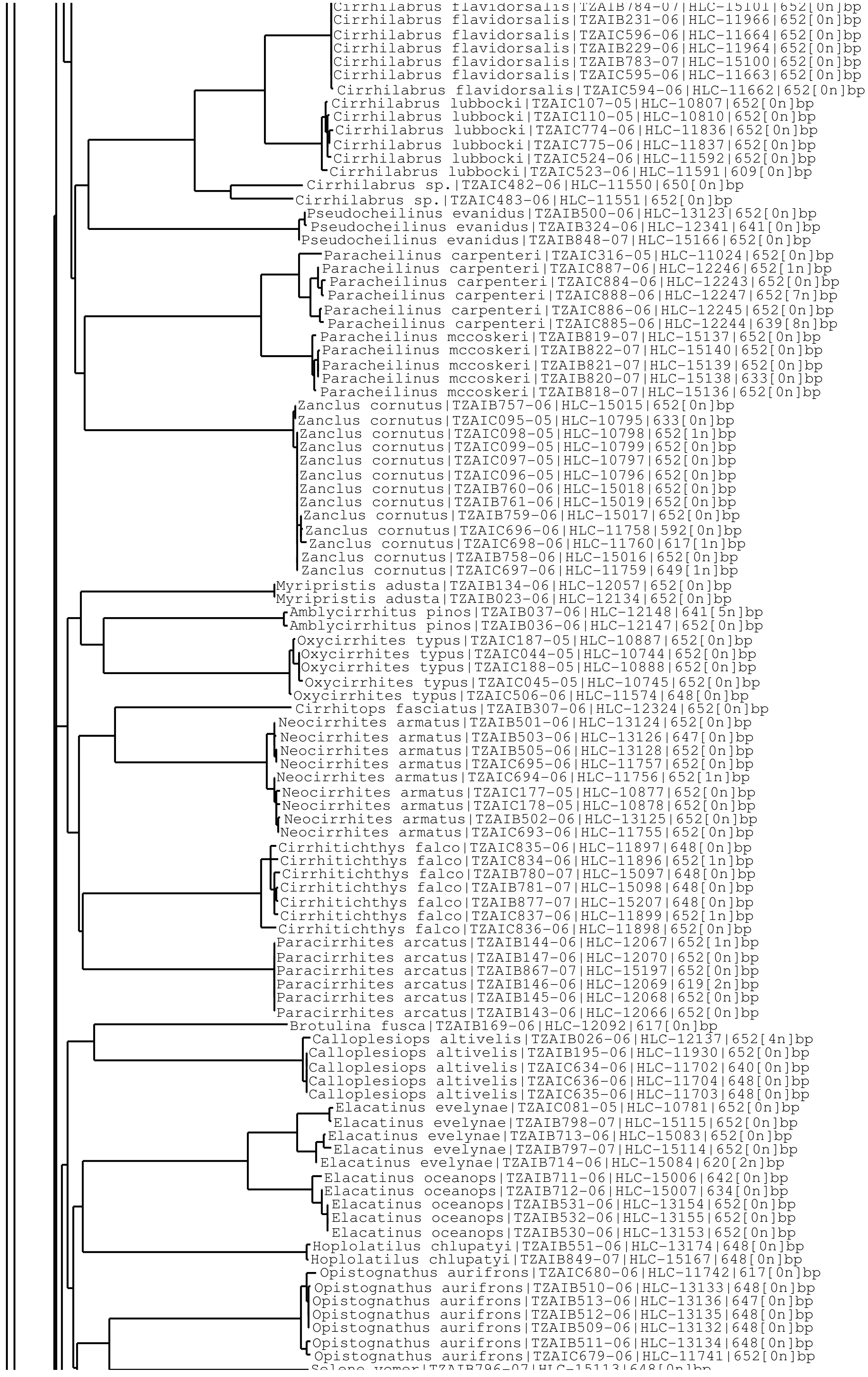
Cirrhilabrus flavidorsalis|TZAIB784-07|HLC-15101|652[0n]bp
Cirrhilabrus flavidorsalis|TZAIB231-06|HLC-11966|652[0n]bp
Cirrhilabrus flavidorsalis|TZAIC596-06|HLC-11664|652[0n]bp
Cirrhilabrus flavidorsalis|TZAIB229-06|HLC-11964|652[0n]bp
Cirrhilabrus flavidorsalis|TZAIB783-07|HLC-15100|652[0n]bp
Cirrhilabrus flavidorsalis|TZAIC595-06|HLC-11663|652[0n]bp
Cirrhilabrus flavidorsalis|TZAIC594-06|HLC-11662|652[0n]bp
Cirrhilabrus lubbocki|TZAIC107-05|HLC-10807|652[0n]bp
Cirrhilabrus lubbocki|TZAIC110-05|HLC-10810|652[0n]bp
Cirrhilabrus lubbocki|TZAIC774-06|HLC-11836|652[0n]bp
Cirrhilabrus lubbocki|TZAIC775-06|HLC-11837|652[0n]bp
Cirrhilabrus lubbocki|TZAIC524-06|HLC-11592|652[0n]bp
Cirrhilabrus lubbocki|TZAIC523-06|HLC-11591|609[0n]bp
Cirrhilabrus sp.|TZAIC482-06|HLC-11550|650[0n]bp
Cirrhilabrus sp.|TZAIC483-06|HLC-11551|652[0n]bp
Pseudocheilinus evanidus|TZAIB500-06|HLC-13123|652[0n]bp
Pseudocheilinus evanidus|TZAIB324-06|HLC-12341|641[0n]bp
Pseudocheilinus evanidus|TZAIB848-07|HLC-15166|652[0n]bp
Paracheilinus carpenteri|TZAIC316-05|HLC-11024|652[0n]bp
Paracheilinus carpenteri|TZAIC887-06|HLC-12246|652[1n]bp
Paracheilinus carpenteri|TZAIC884-06|HLC-12243|652[0n]bp
Paracheilinus carpenteri|TZAIC888-06|HLC-12247|652[7n]bp
Paracheilinus carpenteri|TZAIC886-06|HLC-12245|652[0n]bp
Paracheilinus carpenteri|TZAIC885-06|HLC-12244|639[8n]bp
Paracheilinus mccoskeri|TZAIB819-07|HLC-15137|652[0n]bp
Paracheilinus mccoskeri|TZAIB822-07|HLC-15140|652[0n]bp
Paracheilinus mccoskeri|TZAIB821-07|HLC-15139|652[0n]bp
Paracheilinus mccoskeri|TZAIB820-07|HLC-15138|633[0n]bp
Paracheilinus mccoskeri|TZAIB818-07|HLC-15136|652[0n]bp
Zanclus cornutus|TZAIB757-06|HLC-15015|652[0n]bp
Zanclus cornutus|TZAIC095-05|HLC-10795|633[0n]bp
Zanclus cornutus|TZAIC098-05|HLC-10798|652[1n]bp
Zanclus cornutus|TZAIC099-05|HLC-10799|652[0n]bp
Zanclus cornutus|TZAIC097-05|HLC-10797|652[0n]bp
Zanclus cornutus|TZAIC096-05|HLC-10796|652[0n]bp
Zanclus cornutus|TZAIB760-06|HLC-15018|652[0n]bp
Zanclus cornutus|TZAIB761-06|HLC-15019|652[0n]bp
Zanclus cornutus|TZAIB759-06|HLC-15017|652[0n]bp
Zanclus cornutus|TZAIC696-06|HLC-11758|592[0n]bp
Zanclus cornutus|TZAIC698-06|HLC-11760|617[1n]bp
Zanclus cornutus|TZAIB758-06|HLC-15016|652[0n]bp
Zanclus cornutus|TZAIC697-06|HLC-11759|649[1n]bp
Myripristis adusta|TZAIB134-06|HLC-12057|652[0n]bp
Myripristis adusta|TZAIB023-06|HLC-12134|652[0n]bp
Amblycirrhitus pinos|TZAIB037-06|HLC-12148|641[5n]bp
Amblycirrhitus pinos|TZAIB036-06|HLC-12147|652[0n]bp
Oxycirrhites typus|TZAIC187-05|HLC-10887|652[0n]bp
Oxycirrhites typus|TZAIC044-05|HLC-10744|652[0n]bp
Oxycirrhites typus|TZAIC188-05|HLC-10888|652[0n]bp
Oxycirrhites typus|TZAIC045-05|HLC-10745|652[0n]bp
Oxycirrhites typus|TZAIC506-06|HLC-11574|648[0n]bp
Cirrhitops fasciatus|TZAIB307-06|HLC-12324|652[0n]bp
Neocirrhites armatus|TZAIB501-06|HLC-13124|652[0n]bp
Neocirrhites armatus|TZAIB503-06|HLC-13126|647[0n]bp
Neocirrhites armatus|TZAIB505-06|HLC-13128|652[0n]bp
Neocirrhites armatus|TZAIC695-06|HLC-11757|652[0n]bp
Neocirrhites armatus|TZAIC694-06|HLC-11756|652[1n]bp
Neocirrhites armatus|TZAIC177-05|HLC-10877|652[0n]bp
Neocirrhites armatus|TZAIC178-05|HLC-10878|652[0n]bp
Neocirrhites armatus|TZAIB502-06|HLC-13125|652[0n]bp
Neocirrhites armatus|TZAIC693-06|HLC-11755|652[0n]bp
Cirrhitichthys falco|TZAIC835-06|HLC-11897|648[0n]bp
Cirrhitichthys falco|TZAIC834-06|HLC-11896|652[1n]bp
Cirrhitichthys falco|TZAIB780-07|HLC-15097|648[0n]bp
Cirrhitichthys falco|TZAIB781-07|HLC-15098|648[0n]bp
Cirrhitichthys falco|TZAIB877-07|HLC-15207|648[0n]bp
Cirrhitichthys falco|TZAIC837-06|HLC-11899|652[1n]bp
Cirrhitichthys falco|TZAIC836-06|HLC-11898|652[0n]bp
Paracirrhites arcatus|TZAIB144-06|HLC-12067|652[1n]bp
Paracirrhites arcatus|TZAIB147-06|HLC-12070|652[0n]bp
Paracirrhites arcatus|TZAIB867-07|HLC-15197|652[0n]bp
Paracirrhites arcatus|TZAIB146-06|HLC-12069|619[2n]bp
Paracirrhites arcatus|TZAIB145-06|HLC-12068|652[0n]bp
Paracirrhites arcatus|TZAIB143-06|HLC-12066|652[0n]bp
Brotulina fusca|TZAIB169-06|HLC-12092|617[0n]bp
Calloplesiops altivelis|TZAIB026-06|HLC-12137|652[4n]bp
Calloplesiops altivelis|TZAIB195-06|HLC-11930|652[0n]bp
Calloplesiops altivelis|TZAIC634-06|HLC-11702|640[0n]bp
Calloplesiops altivelis|TZAIC636-06|HLC-11704|648[0n]bp
Calloplesiops altivelis|TZAIC635-06|HLC-11703|648[0n]bp
Elacatinus evelynae|TZAIC081-05|HLC-10781|652[0n]bp
Elacatinus evelynae|TZAIB798-07|HLC-15115|652[0n]bp
Elacatinus evelynae|TZAIB713-06|HLC-15083|652[0n]bp
Elacatinus evelynae|TZAIB797-07|HLC-15114|652[0n]bp
Elacatinus evelynae|TZAIB714-06|HLC-15084|620[2n]bp
Elacatinus oceanops|TZAIB711-06|HLC-15006|642[0n]bp
Elacatinus oceanops|TZAIB712-06|HLC-15007|634[0n]bp
Elacatinus oceanops|TZAIB531-06|HLC-13154|652[0n]bp
Elacatinus oceanops|TZAIB532-06|HLC-13155|652[0n]bp
Elacatinus oceanops|TZAIB530-06|HLC-13153|652[0n]bp
Hoplolatilus chlupatyi|TZAIB551-06|HLC-13174|648[0n]bp
Hoplolatilus chlupatyi|TZAIB849-07|HLC-15167|648[0n]bp
Opistognathus aurifrons|TZAIC680-06|HLC-11742|617[0n]bp
Opistognathus aurifrons|TZAIB510-06|HLC-13133|648[0n]bp
Opistognathus aurifrons|TZAIB513-06|HLC-13136|647[0n]bp
Opistognathus aurifrons|TZAIB512-06|HLC-13135|648[0n]bp
Opistognathus aurifrons|TZAIB509-06|HLC-13132|648[0n]bp
Opistognathus aurifrons|TZAIB511-06|HLC-13134|648[0n]bp
Opistognathus aurifrons|TZAIC679-06|HLC-11741|652[0n]bp
Selene vomer|TZAIB796-07|HLC-15113|648[0n]bp

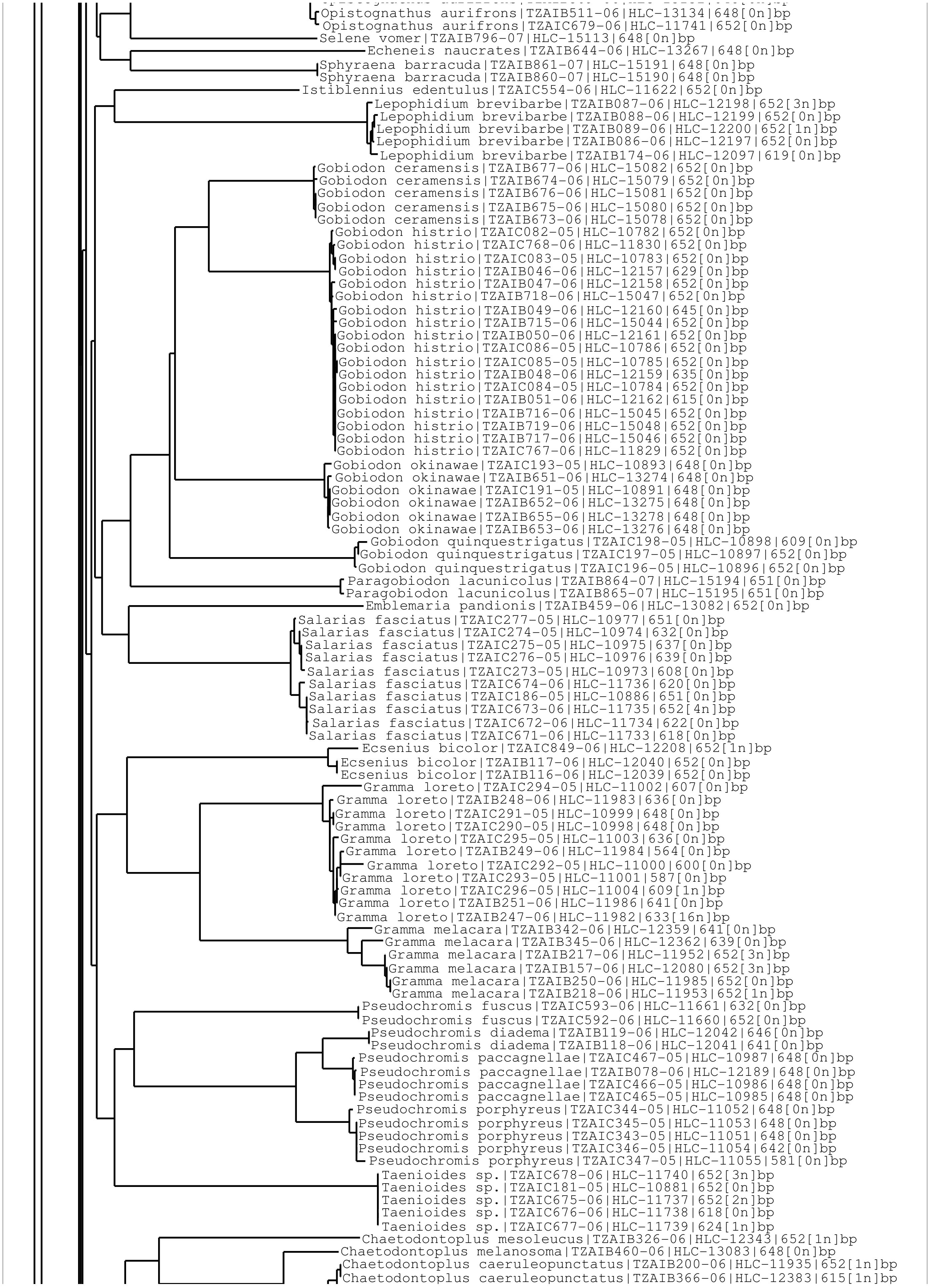
Opistognathus aurifrons|TZAIB511-06|HLC-13134|648[0n]bp
Opistognathus aurifrons|TZAIC679-06|HLC-11741|652[0n]bp
Selene vomer|TZAIB796-07|HLC-15113|648[0n]bp
Echeneis naucrates|TZAIB644-06|HLC-13267|648[0n]bp
Sphyraena barracuda|TZAIB861-07|HLC-15191|648[0n]bp
Sphyraena barracuda|TZAIB860-07|HLC-15190|648[0n]bp
Istiblennius edentulus|TZAIC554-06|HLC-11622|652[0n]bp
Lepophidium brevibarbe|TZAIB087-06|HLC-12198|652[3n]bp
Lepophidium brevibarbe|TZAIB088-06|HLC-12199|652[0n]bp
Lepophidium brevibarbe|TZAIB089-06|HLC-12200|652[1n]bp
Lepophidium brevibarbe|TZAIB086-06|HLC-12197|652[0n]bp
Lepophidium brevibarbe|TZAIB174-06|HLC-12097|619[0n]bp
Gobiodon ceramensis|TZAIB677-06|HLC-15082|652[0n]bp
Gobiodon ceramensis|TZAIB674-06|HLC-15079|652[0n]bp
Gobiodon ceramensis|TZAIB676-06|HLC-15081|652[0n]bp
Gobiodon ceramensis|TZAIB675-06|HLC-15080|652[0n]bp
Gobiodon ceramensis|TZAIB673-06|HLC-15078|652[0n]bp
Gobiodon histrio|TZAIC082-05|HLC-10782|652[0n]bp
Gobiodon histrio|TZAIC768-06|HLC-11830|652[0n]bp
Gobiodon histrio|TZAIC083-05|HLC-10783|652[0n]bp
Gobiodon histrio|TZAIB046-06|HLC-12157|629[0n]bp
Gobiodon histrio|TZAIB047-06|HLC-12158|652[0n]bp
Gobiodon histrio|TZAIB718-06|HLC-15047|652[0n]bp
Gobiodon histrio|TZAIB049-06|HLC-12160|645[0n]bp
Gobiodon histrio|TZAIB715-06|HLC-15044|652[0n]bp
Gobiodon histrio|TZAIB050-06|HLC-12161|652[0n]bp
Gobiodon histrio|TZAIC086-05|HLC-10786|652[0n]bp
Gobiodon histrio|TZAIC085-05|HLC-10785|652[0n]bp
Gobiodon histrio|TZAIB048-06|HLC-12159|635[0n]bp
Gobiodon histrio|TZAIC084-05|HLC-10784|652[0n]bp
Gobiodon histrio|TZAIB051-06|HLC-12162|615[0n]bp
Gobiodon histrio|TZAIB716-06|HLC-15045|652[0n]bp
Gobiodon histrio|TZAIB719-06|HLC-15048|652[0n]bp
Gobiodon histrio|TZAIB717-06|HLC-15046|652[0n]bp
Gobiodon histrio|TZAIC767-06|HLC-11829|652[0n]bp
Gobiodon okinawae|TZAIC193-05|HLC-10893|648[0n]bp
Gobiodon okinawae|TZAIB651-06|HLC-13274|648[0n]bp
Gobiodon okinawae|TZAIC191-05|HLC-10891|648[0n]bp
Gobiodon okinawae|TZAIB652-06|HLC-13275|648[0n]bp
Gobiodon okinawae|TZAIB655-06|HLC-13278|648[0n]bp
Gobiodon okinawae|TZAIB653-06|HLC-13276|648[0n]bp
Gobiodon quinquestrigatus|TZAIC198-05|HLC-10898|609[0n]bp
Gobiodon quinquestrigatus|TZAIC197-05|HLC-10897|652[0n]bp
Gobiodon quinquestrigatus|TZAIC196-05|HLC-10896|652[0n]bp
Paragobiodon lacunicolus|TZAIB864-07|HLC-15194|651[0n]bp
Paragobiodon lacunicolus|TZAIB865-07|HLC-15195|651[0n]bp
Emblemaria pandionis|TZAIB459-06|HLC-13082|652[0n]bp
Salarias fasciatus|TZAIC277-05|HLC-10977|651[0n]bp
Salarias fasciatus|TZAIC274-05|HLC-10974|632[0n]bp
Salarias fasciatus|TZAIC275-05|HLC-10975|637[0n]bp
Salarias fasciatus|TZAIC276-05|HLC-10976|639[0n]bp
Salarias fasciatus|TZAIC273-05|HLC-10973|608[0n]bp
Salarias fasciatus|TZAIC674-06|HLC-11736|620[0n]bp
Salarias fasciatus|TZAIC186-05|HLC-10886|651[0n]bp
Salarias fasciatus|TZAIC673-06|HLC-11735|652[4n]bp
Salarias fasciatus|TZAIC672-06|HLC-11734|622[0n]bp
Salarias fasciatus|TZAIC671-06|HLC-11733|618[0n]bp
Ecsenius bicolor|TZAIC849-06|HLC-12208|652[1n]bp
Ecsenius bicolor|TZAIB117-06|HLC-12040|652[0n]bp
Ecsenius bicolor|TZAIB116-06|HLC-12039|652[0n]bp
Gramma loreto|TZAIC294-05|HLC-11002|607[0n]bp
Gramma loreto|TZAIB248-06|HLC-11983|636[0n]bp
Gramma loreto|TZAIC291-05|HLC-10999|648[0n]bp
Gramma loreto|TZAIC290-05|HLC-10998|648[0n]bp
Gramma loreto|TZAIC295-05|HLC-11003|636[0n]bp
Gramma loreto|TZAIB249-06|HLC-11984|564[0n]bp
Gramma loreto|TZAIC292-05|HLC-11000|600[0n]bp
Gramma loreto|TZAIC293-05|HLC-11001|587[0n]bp
Gramma loreto|TZAIC296-05|HLC-11004|609[1n]bp
Gramma loreto|TZAIB251-06|HLC-11986|641[0n]bp
Gramma loreto|TZAIB247-06|HLC-11982|633[16n]bp
Gramma melacara|TZAIB342-06|HLC-12359|641[0n]bp
Gramma melacara|TZAIB345-06|HLC-12362|639[0n]bp
Gramma melacara|TZAIB217-06|HLC-11952|652[3n]bp
Gramma melacara|TZAIB157-06|HLC-12080|652[3n]bp
Gramma melacara|TZAIB250-06|HLC-11985|652[0n]bp
Gramma melacara|TZAIB218-06|HLC-11953|652[1n]bp
Pseudochromis fuscus|TZAIC593-06|HLC-11661|632[0n]bp
Pseudochromis fuscus|TZAIC592-06|HLC-11660|652[0n]bp
Pseudochromis diadema|TZAIB119-06|HLC-12042|646[0n]bp
Pseudochromis diadema|TZAIB118-06|HLC-12041|641[0n]bp
Pseudochromis paccagnellae|TZAIC467-05|HLC-10987|648[0n]bp
Pseudochromis paccagnellae|TZAIB078-06|HLC-12189|648[0n]bp
Pseudochromis paccagnellae|TZAIC466-05|HLC-10986|648[0n]bp
Pseudochromis paccagnellae|TZAIC465-05|HLC-10985|648[0n]bp
Pseudochromis porphyreus|TZAIC344-05|HLC-11052|648[0n]bp
Pseudochromis porphyreus|TZAIC345-05|HLC-11053|648[0n]bp
Pseudochromis porphyreus|TZAIC343-05|HLC-11051|648[0n]bp
Pseudochromis porphyreus|TZAIC346-05|HLC-11054|642[0n]bp
Pseudochromis porphyreus|TZAIC347-05|HLC-11055|581[0n]bp
Taenioides sp.|TZAIC678-06|HLC-11740|652[3n]bp
Taenioides sp.|TZAIC181-05|HLC-10881|652[0n]bp
Taenioides sp.|TZAIC675-06|HLC-11737|652[2n]bp
Taenioides sp.|TZAIC676-06|HLC-11738|618[0n]bp
Taenioides sp.|TZAIC677-06|HLC-11739|624[1n]bp
Chaetodontoplus mesoleucus|TZAIB326-06|HLC-12343|652[1n]bp
Chaetodontoplus melanosoma|TZAIB460-06|HLC-13083|648[0n]bp
Chaetodontoplus caeruleopunctatus|TZAIB200-06|HLC-11935|652[1n]bp
Chaetodontoplus caeruleopunctatus|TZAIB366-06|HLC-12383|615[1n]bp

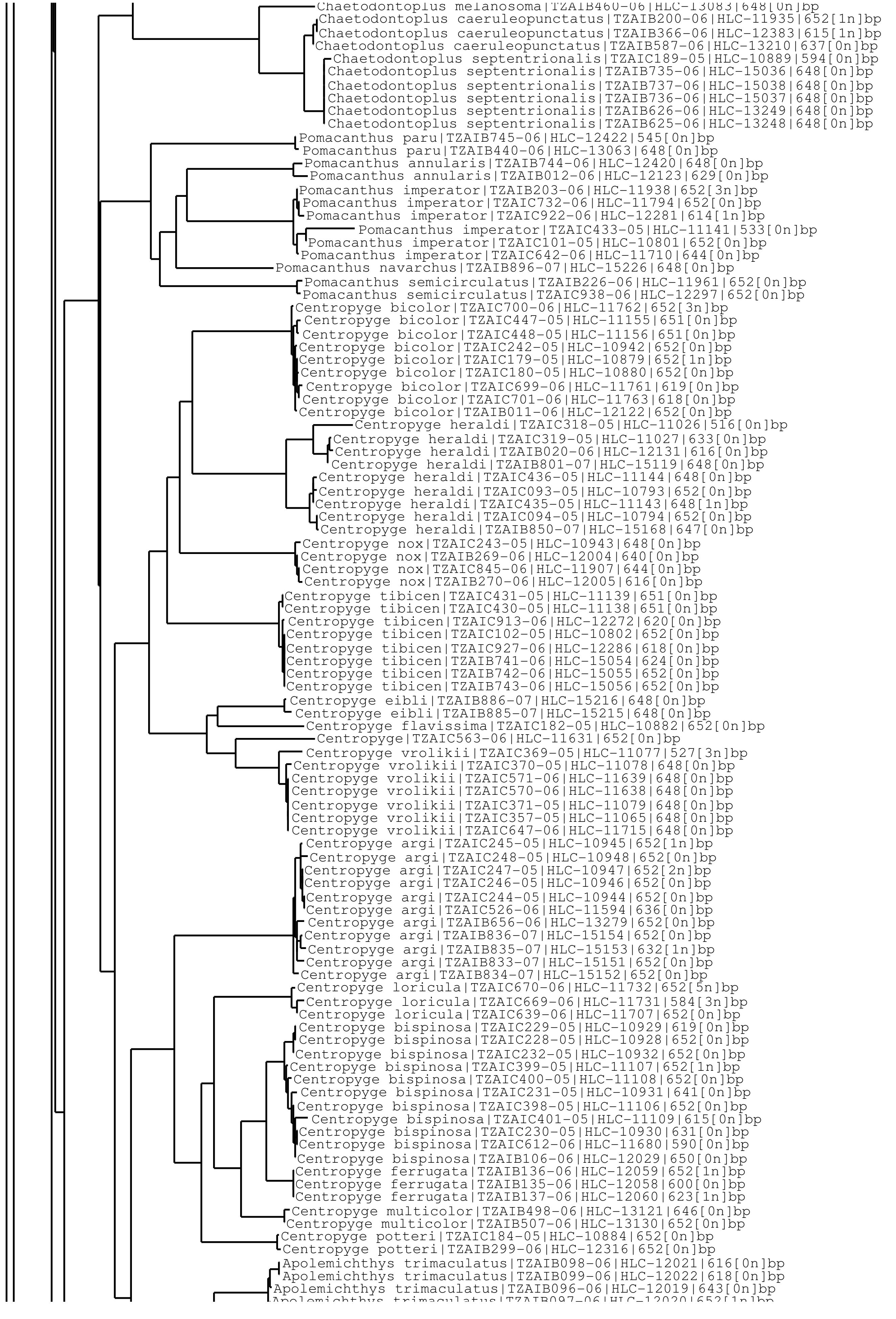

Chaetodontoplus melanosoma|TZAIB460-06|HLC-13083|648[0n]bp
Chaetodontoplus caeruleopunctatus|TZAIB200-06|HLC-11935|652[1n]bp
Chaetodontoplus caeruleopunctatus|TZAIB366-06|HLC-12383|615[1n]bp
Chaetodontoplus caeruleopunctatus|TZAIB587-06|HLC-13210|637[0n]bp
Chaetodontoplus septentrionalis|TZAIC189-05|HLC-10889|594[0n]bp
Chaetodontoplus septentrionalis|TZAIB735-06|HLC-15036|648[0n]bp
Chaetodontoplus septentrionalis|TZAIB737-06|HLC-15038|648[0n]bp
Chaetodontoplus septentrionalis|TZAIB736-06|HLC-15037|648[0n]bp
Chaetodontoplus septentrionalis|TZAIB626-06|HLC-13249|648[0n]bp
Chaetodontoplus septentrionalis|TZAIB625-06|HLC-13248|648[0n]bp
Pomacanthus paru|TZAIB745-06|HLC-12422|545[0n]bp
Pomacanthus paru|TZAIB440-06|HLC-13063|648[0n]bp
Pomacanthus annularis|TZAIB744-06|HLC-12420|648[0n]bp
Pomacanthus annularis|TZAIB012-06|HLC-12123|629[0n]bp
Pomacanthus imperator|TZAIB203-06|HLC-11938|652[3n]bp
Pomacanthus imperator|TZAIC732-06|HLC-11794|652[0n]bp
Pomacanthus imperator|TZAIC922-06|HLC-12281|614[1n]bp
Pomacanthus imperator|TZAIC433-05|HLC-11141|533[0n]bp
Pomacanthus imperator|TZAIC101-05|HLC-10801|652[0n]bp
Pomacanthus imperator|TZAIC642-06|HLC-11710|644[0n]bp
Pomacanthus navarchus|TZAIB896-07|HLC-15226|648[0n]bp
Pomacanthus semicirculatus|TZAIB226-06|HLC-11961|652[0n]bp
Pomacanthus semicirculatus|TZAIC938-06|HLC-12297|652[0n]bp
Centropyge bicolor|TZAIC700-06|HLC-11762|652[3n]bp
Centropyge bicolor|TZAIC447-05|HLC-11155|651[0n]bp
Centropyge bicolor|TZAIC448-05|HLC-11156|651[0n]bp
Centropyge bicolor|TZAIC242-05|HLC-10942|652[0n]bp
Centropyge bicolor|TZAIC179-05|HLC-10879|652[1n]bp
Centropyge bicolor|TZAIC180-05|HLC-10880|652[0n]bp
Centropyge bicolor|TZAIC699-06|HLC-11761|619[0n]bp
Centropyge bicolor|TZAIC701-06|HLC-11763|618[0n]bp
Centropyge bicolor|TZAIB011-06|HLC-12122|652[0n]bp
Centropyge heraldi|TZAIC318-05|HLC-11026|516[0n]bp
Centropyge heraldi|TZAIC319-05|HLC-11027|633[0n]bp
Centropyge heraldi|TZAIB020-06|HLC-12131|616[0n]bp
Centropyge heraldi|TZAIB801-07|HLC-15119|648[0n]bp
Centropyge heraldi|TZAIC436-05|HLC-11144|648[0n]bp
Centropyge heraldi|TZAIC093-05|HLC-10793|652[0n]bp
Centropyge heraldi|TZAIC435-05|HLC-11143|648[1n]bp
Centropyge heraldi|TZAIC094-05|HLC-10794|652[0n]bp
Centropyge heraldi|TZAIB850-07|HLC-15168|647[0n]bp
Centropyge nox|TZAIC243-05|HLC-10943|648[0n]bp
Centropyge nox|TZAIB269-06|HLC-12004|640[0n]bp
Centropyge nox|TZAIC845-06|HLC-11907|644[0n]bp
Centropyge nox|TZAIB270-06|HLC-12005|616[0n]bp
Centropyge tibicen|TZAIC431-05|HLC-11139|651[0n]bp
Centropyge tibicen|TZAIC430-05|HLC-11138|651[0n]bp
Centropyge tibicen|TZAIC913-06|HLC-12272|620[0n]bp
Centropyge tibicen|TZAIC102-05|HLC-10802|652[0n]bp
Centropyge tibicen|TZAIC927-06|HLC-12286|618[0n]bp
Centropyge tibicen|TZAIB741-06|HLC-15054|624[0n]bp
Centropyge tibicen|TZAIB742-06|HLC-15055|652[0n]bp
Centropyge tibicen|TZAIB743-06|HLC-15056|652[0n]bp
Centropyge eibli|TZAIB886-07|HLC-15216|648[0n]bp
Centropyge eibli|TZAIB885-07|HLC-15215|648[0n]bp
Centropyge flavissima|TZAIC182-05|HLC-10882|652[0n]bp
Centropyge|TZAIC563-06|HLC-11631|652[0n]bp
Centropyge vrolikii|TZAIC369-05|HLC-11077|527[3n]bp
Centropyge vrolikii|TZAIC370-05|HLC-11078|648[0n]bp
Centropyge vrolikii|TZAIC571-06|HLC-11639|648[0n]bp
Centropyge vrolikii|TZAIC570-06|HLC-11638|648[0n]bp
Centropyge vrolikii|TZAIC371-05|HLC-11079|648[0n]bp
Centropyge vrolikii|TZAIC357-05|HLC-11065|648[0n]bp
Centropyge vrolikii|TZAIC647-06|HLC-11715|648[0n]bp
Centropyge argi|TZAIC245-05|HLC-10945|652[1n]bp
Centropyge argi|TZAIC248-05|HLC-10948|652[0n]bp
Centropyge argi|TZAIC247-05|HLC-10947|652[2n]bp
Centropyge argi|TZAIC246-05|HLC-10946|652[0n]bp
Centropyge argi|TZAIC244-05|HLC-10944|652[0n]bp
Centropyge argi|TZAIC526-06|HLC-11594|636[0n]bp
Centropyge argi|TZAIB656-06|HLC-13279|652[0n]bp
Centropyge argi|TZAIB836-07|HLC-15154|652[0n]bp
Centropyge argi|TZAIB835-07|HLC-15153|632[1n]bp
Centropyge argi|TZAIB833-07|HLC-15151|652[0n]bp
Centropyge argi|TZAIB834-07|HLC-15152|652[0n]bp
Centropyge loricula|TZAIC670-06|HLC-11732|652[5n]bp
Centropyge loricula|TZAIC669-06|HLC-11731|584[3n]bp
Centropyge loricula|TZAIC639-06|HLC-11707|652[0n]bp
Centropyge bispinosa|TZAIC229-05|HLC-10929|619[0n]bp
Centropyge bispinosa|TZAIC228-05|HLC-10928|652[0n]bp
Centropyge bispinosa|TZAIC232-05|HLC-10932|652[0n]bp
Centropyge bispinosa|TZAIC399-05|HLC-11107|652[1n]bp
Centropyge bispinosa|TZAIC400-05|HLC-11108|652[0n]bp
Centropyge bispinosa|TZAIC231-05|HLC-10931|641[0n]bp
Centropyge bispinosa|TZAIC398-05|HLC-11106|652[0n]bp
Centropyge bispinosa|TZAIC401-05|HLC-11109|615[0n]bp
Centropyge bispinosa|TZAIC230-05|HLC-10930|631[0n]bp
Centropyge bispinosa|TZAIC612-06|HLC-11680|590[0n]bp
Centropyge bispinosa|TZAIB106-06|HLC-12029|650[0n]bp
Centropyge ferrugata|TZAIB136-06|HLC-12059|652[1n]bp
Centropyge ferrugata|TZAIB135-06|HLC-12058|600[0n]bp
Centropyge ferrugata|TZAIB137-06|HLC-12060|623[1n]bp
Centropyge multicolor|TZAIB498-06|HLC-13121|646[0n]bp
Centropyge multicolor|TZAIB507-06|HLC-13130|652[0n]bp
Centropyge potteri|TZAIC184-05|HLC-10884|652[0n]bp
Centropyge potteri|TZAIB299-06|HLC-12316|652[0n]bp
Apolemichthys trimaculatus|TZAIB098-06|HLC-12021|616[0n]bp
Apolemichthys trimaculatus|TZAIB099-06|HLC-12022|618[0n]bp
Apolemichthys trimaculatus|TZAIB096-06|HLC-12019|643[0n]bp
Apolemichthys trimaculatus|TZAIB097-06|HLC-12020|652[1n]bp

[illegible]
Apolemichthys trimaculatus|TZAIB099-06|HLC-12022|618[0n]bp
Apolemichthys trimaculatus|TZAIB096-06|HLC-12019|643[0n]bp
Apolemichthys trimaculatus|TZAIB097-06|HLC-12020|652[1n]bp
Apolemichthys trimaculatus|TZAIB323-06|HLC-12340|652[0n]bp
Apolemichthys xanthurus|TZAIB368-06|HLC-12385|652[0n]bp
Apolemichthys xanthurus|TZAIB565-06|HLC-13188|648[0n]bp
Centropyge multifasciata|TZAIC404-05|HLC-11112|652[0n]bp
Centropyge venustus|TZAIC661-06|HLC-11173|648[0n]bp
Centropyge venustus|TZAIC682-06|HLC-11744|637[0n]bp
Genicanthus lamarck|TZAIB899-07|HLC-15229|652[0n]bp
Genicanthus lamarck|TZAIB042-06|HLC-12153|641[7n]bp
Genicanthus lamarck|TZAIB902-07|HLC-15232|652[0n]bp
Genicanthus lamarck|TZAIB900-07|HLC-15230|652[0n]bp
Genicanthus lamarck|TZAIB901-07|HLC-15231|652[0n]bp
Genicanthus lamarck|TZAIB039-06|HLC-12150|652[3n]bp
Genicanthus lamarck|TZAIB040-06|HLC-12151|651[1n]bp
Genicanthus lamarck|TZAIB041-06|HLC-12152|652[2n]bp
Genicanthus lamarck|TZAIB038-06|HLC-12149|652[3n]bp
Genicanthus melanospilos|TZAIB024-06|HLC-12135|648[0n]bp
Genicanthus melanospilos|TZAIC353-05|HLC-11061|648[0n]bp
Genicanthus melanospilos|TZAIC921-06|HLC-12280|652[0n]bp
Genicanthus watanabei|TZAIB322-06|HLC-12339|652[0n]bp
Holacanthus ciliaris|TZAIB740-06|HLC-12425|648[0n]bp
Holacanthus ciliaris|TZAIB738-06|HLC-12423|648[0n]bp
Holacanthus ciliaris|TZAIB739-06|HLC-12424|648[0n]bp
Holacanthus ciliaris|TZAIB432-06|HLC-13055|648[0n]bp
Holacanthus ciliaris|TZAIB649-06|HLC-13272|648[0n]bp
Holacanthus tricolor|TZAIB792-07|HLC-15109|648[0n]bp
Holacanthus tricolor|TZAIB259-06|HLC-11994|652[1n]bp
Holacanthus tricolor|TZAIB257-06|HLC-11992|648[0n]bp
Holacanthus tricolor|TZAIB258-06|HLC-11993|618[0n]bp
Holacanthus tricolor|TZAIB261-06|HLC-11996|652[0n]bp
Holacanthus tricolor|TZAIB260-06|HLC-11995|652[0n]bp
Pygoplites diacanthus|TZAIC358-05|HLC-11066|652[0n]bp
Pygoplites diacanthus|TZAIB108-06|HLC-12031|640[1n]bp
Pygoplites diacanthus|TZAIB107-06|HLC-12030|629[0n]bp
Pygoplites diacanthus|TZAIB374-06|HLC-12391|619[0n]bp
Scatophagus argus|TZAIB882-07|HLC-15212|652[0n]bp
Scatophagus argus|TZAIB884-07|HLC-15214|652[0n]bp
Scatophagus argus|TZAIB883-07|HLC-15213|652[0n]bp
Scatophagus argus|TZAIB881-07|HLC-15211|652[0n]bp
Scatophagus argus|TZAIC331-05|HLC-11039|652[0n]bp
Acanthostracion quadricornis|TZAIB005-06|HLC-12116|611[0n]bp
Acanthostracion quadricornis|TZAIB006-06|HLC-12117|620[0n]bp
Acanthostracion quadricornis|TZAIB007-06|HLC-12118|652[1n]bp
Acanthostracion quadricornis|TZAIB008-06|HLC-12119|652[0n]bp
Acanthostracion quadricornis|TZAIB009-06|HLC-12120|652[4n]bp
Lactophrys bicaudalis|TZAIB351-06|HLC-12368|652[0n]bp
Lactoria cornuta|TZAIB607-06|HLC-13230|648[0n]bp
Lactoria cornuta|TZAIC751-06|HLC-11813|652[0n]bp
Lactoria cornuta|TZAIC750-06|HLC-11812|652[0n]bp
Lactoria cornuta|TZAIB608-06|HLC-13231|648[0n]bp
Lactoria cornuta|TZAIC464-05|HLC-10984|647[0n]bp
Lactoria cornuta|TZAIC004-05|HLC-TSZ-004|648[0n]bp
Lactoria cornuta|TZAIC008-05|HLC-TSZ-008|652[0n]bp
Lactoria cornuta|TZAIC423-05|HLC-11131|648[0n]bp
Lactoria cornuta|TZAIC007-05|HLC-TSZ-007|652[0n]bp
Lactoria cornuta|TZAIC006-05|HLC-TSZ-006|652[0n]bp
Lactoria cornuta|TZAIC005-05|HLC-TSZ-005|652[0n]bp
Lactoria cornuta|TZAIC731-06|HLC-11793|652[2n]bp
Lactoria cornuta|TZAIB609-06|HLC-13232|648[0n]bp
Lactoria cornuta|TZAIB610-06|HLC-13233|633[0n]bp
Lactoria cornuta|TZAIC749-06|HLC-11811|652[0n]bp
Lactoria cornuta|TZAIB611-06|HLC-13234|648[0n]bp
Ostracion|TZAIC508-06|HLC-11576|652[1n]bp
Ostracion meleagris|TZAIB338-06|HLC-12355|652[0n]bp
Ostracion meleagris|TZAIB843-07|HLC-15161|647[2n]bp
Ostracion meleagris|TZAIB526-06|HLC-13149|630[0n]bp
Ostracion meleagris|TZAIB845-07|HLC-15163|647[0n]bp
Ostracion meleagris|TZAIC777-06|HLC-11839|648[0n]bp
Ostracion meleagris|TZAIB340-06|HLC-12357|652[1n]bp
Ostracion meleagris|TZAIB339-06|HLC-12356|652[0n]bp
Ostracion meleagris|TZAIB337-06|HLC-12354|652[1n]bp
Ostracion meleagris|TZAIB844-07|HLC-15162|648[0n]bp
Ostracion meleagris|TZAIC668-06|HLC-11730|652[0n]bp
Ostracion nasus|TZAIB094-06|HLC-12205|627[0n]bp
Ostracion nasus|TZAIC848-06|HLC-12207|652[1n]bp
Ostracion nasus|TZAIC796-06|HLC-11858|652[1n]bp
Ostracion cubicus|TZAIB769-06|HLC-15069|647[1n]bp
Ostracion cubicus|TZAIB770-06|HLC-15070|648[0n]bp
Ostracion cubicus|TZAIB768-06|HLC-15068|648[0n]bp
Ostracion cubicus|TZAIB767-06|HLC-15067|648[0n]bp
Ostracion cubicus|TZAIB771-06|HLC-15071|648[0n]bp
Ostracion cubicus|TZAIC507-06|HLC-11575|642[0n]bp
Ostracion rhinorhynchos|TZAIC780-06|HLC-11842|652[1n]bp
Tetrosomus gibbosus|TZAIB793-07|HLC-15110|648[0n]bp
Halichoeres marginatus|TZAIC527-06|HLC-11595|652[0n]bp
Halichoeres marginatus|TZAIC266-05|HLC-10966|652[0n]bp
Halichoeres marginatus|TZAIC149-05|HLC-10849|652[0n]bp
Halichoeres melanurus|TZAIC267-05|HLC-10967|652[0n]bp
Halichoeres melanurus|TZAIC826-06|HLC-11888|630[0n]bp
Halichoeres melanurus|TZAIC265-05|HLC-10965|652[0n]bp
Halichoeres melanurus|TZAIC269-05|HLC-10969|652[0n]bp
Halichoeres melanurus|TZAIC268-05|HLC-10968|652[0n]bp
Halichoeres melanurus|TZAIC147-05|HLC-10847|652[0n]bp
Halichoeres melanurus|TZAIC824-06|HLC-11886|652[0n]bp
Halichoeres melanurus|TZAIC148-05|HLC-10848|652[0n]bp
Halichoeres melanurus|TZAIC827-06|HLC-11889|652[0n]bp
Halichoeres melanurus|TZAIC825-06|HLC-11887|652[0n]bp

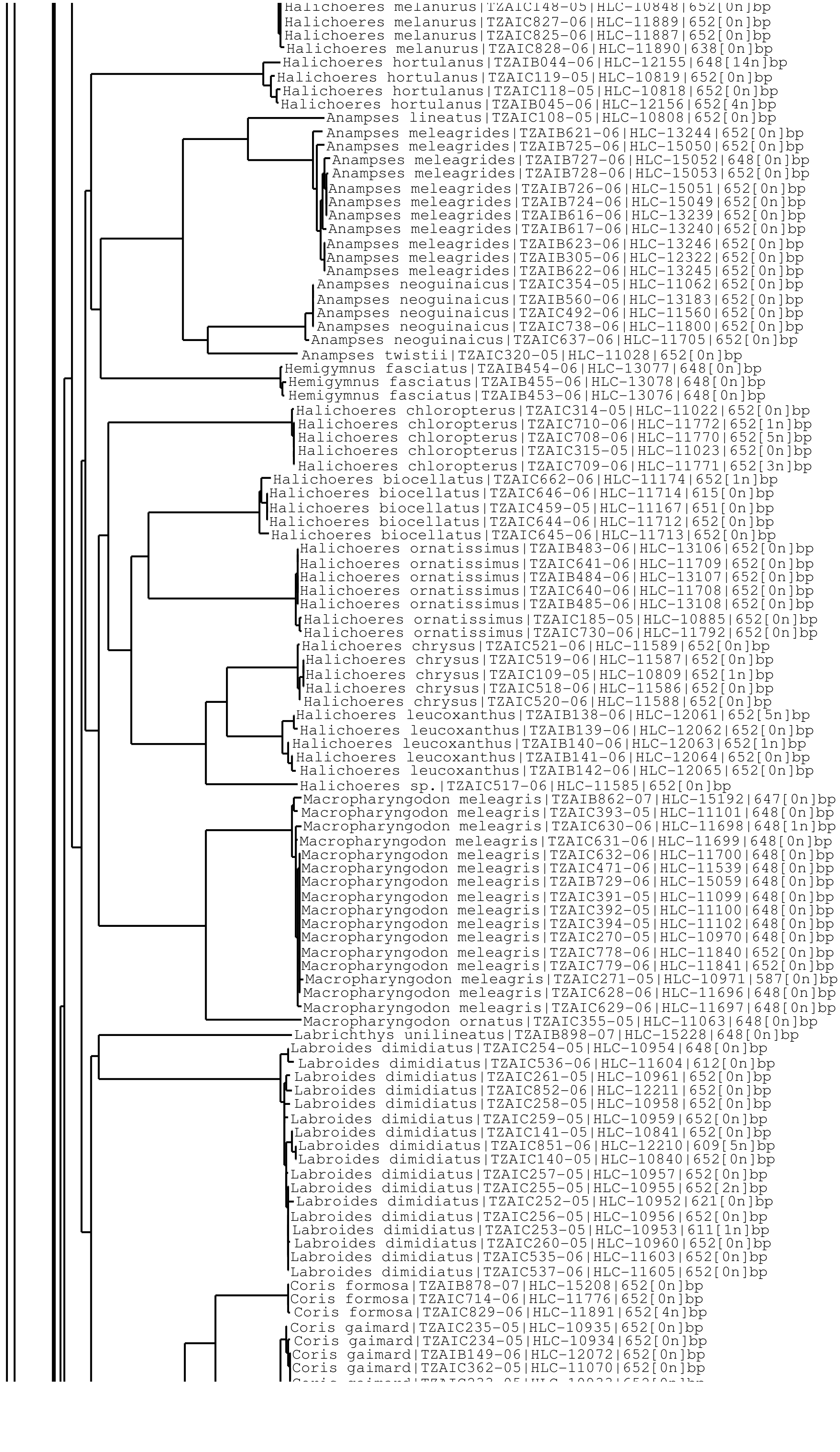
Halichoeres melanurus|TZAIC148-05|HLC-10848|652[0n]bp
Halichoeres melanurus|TZAIC827-06|HLC-11889|652[0n]bp
Halichoeres melanurus|TZAIC825-06|HLC-11887|652[0n]bp
Halichoeres melanurus|TZAIC828-06|HLC-11890|638[0n]bp
Halichoeres hortulanus|TZAIB044-06|HLC-12155|648[14n]bp
Halichoeres hortulanus|TZAIC119-05|HLC-10819|652[0n]bp
Halichoeres hortulanus|TZAIC118-05|HLC-10818|652[0n]bp
Halichoeres hortulanus|TZAIB045-06|HLC-12156|652[4n]bp
Anampses lineatus|TZAIC108-05|HLC-10808|652[0n]bp
Anampses meleagrides|TZAIB621-06|HLC-13244|652[0n]bp
Anampses meleagrides|TZAIB725-06|HLC-15050|652[0n]bp
Anampses meleagrides|TZAIB727-06|HLC-15052|648[0n]bp
Anampses meleagrides|TZAIB728-06|HLC-15053|652[0n]bp
Anampses meleagrides|TZAIB726-06|HLC-15051|652[0n]bp
Anampses meleagrides|TZAIB724-06|HLC-15049|652[0n]bp
Anampses meleagrides|TZAIB616-06|HLC-13239|652[0n]bp
Anampses meleagrides|TZAIB617-06|HLC-13240|652[0n]bp
Anampses meleagrides|TZAIB623-06|HLC-13246|652[0n]bp
Anampses meleagrides|TZAIB305-06|HLC-12322|652[0n]bp
Anampses meleagrides|TZAIB622-06|HLC-13245|652[0n]bp
Anampses neoguinaicus|TZAIC354-05|HLC-11062|652[0n]bp
Anampses neoguinaicus|TZAIB560-06|HLC-13183|652[0n]bp
Anampses neoguinaicus|TZAIC492-06|HLC-11560|652[0n]bp
Anampses neoguinaicus|TZAIC738-06|HLC-11800|652[0n]bp
Anampses neoguinaicus|TZAIC637-06|HLC-11705|652[0n]bp
Anampses twistii|TZAIC320-05|HLC-11028|652[0n]bp
Hemigymnus fasciatus|TZAIB454-06|HLC-13077|648[0n]bp
Hemigymnus fasciatus|TZAIB455-06|HLC-13078|648[0n]bp
Hemigymnus fasciatus|TZAIB453-06|HLC-13076|648[0n]bp
Halichoeres chloropterus|TZAIC314-05|HLC-11022|652[0n]bp
Halichoeres chloropterus|TZAIC710-06|HLC-11772|652[1n]bp
Halichoeres chloropterus|TZAIC708-06|HLC-11770|652[5n]bp
Halichoeres chloropterus|TZAIC315-05|HLC-11023|652[0n]bp
Halichoeres chloropterus|TZAIC709-06|HLC-11771|652[3n]bp
Halichoeres biocellatus|TZAIC662-06|HLC-11174|652[1n]bp
Halichoeres biocellatus|TZAIC646-06|HLC-11714|615[0n]bp
Halichoeres biocellatus|TZAIC459-05|HLC-11167|651[0n]bp
Halichoeres biocellatus|TZAIC644-06|HLC-11712|652[0n]bp
Halichoeres biocellatus|TZAIC645-06|HLC-11713|652[0n]bp
Halichoeres ornatissimus|TZAIB483-06|HLC-13106|652[0n]bp
Halichoeres ornatissimus|TZAIC641-06|HLC-11709|652[0n]bp
Halichoeres ornatissimus|TZAIB484-06|HLC-13107|652[0n]bp
Halichoeres ornatissimus|TZAIC640-06|HLC-11708|652[0n]bp
Halichoeres ornatissimus|TZAIB485-06|HLC-13108|652[0n]bp
Halichoeres ornatissimus|TZAIC185-05|HLC-10885|652[0n]bp
Halichoeres ornatissimus|TZAIC730-06|HLC-11792|652[0n]bp
Halichoeres chrysus|TZAIC521-06|HLC-11589|652[0n]bp
Halichoeres chrysus|TZAIC519-06|HLC-11587|652[0n]bp
Halichoeres chrysus|TZAIC109-05|HLC-10809|652[1n]bp
Halichoeres chrysus|TZAIC518-06|HLC-11586|652[0n]bp
Halichoeres chrysus|TZAIC520-06|HLC-11588|652[0n]bp
Halichoeres leucoxanthus|TZAIB138-06|HLC-12061|652[5n]bp
Halichoeres leucoxanthus|TZAIB139-06|HLC-12062|652[0n]bp
Halichoeres leucoxanthus|TZAIB140-06|HLC-12063|652[1n]bp
Halichoeres leucoxanthus|TZAIB141-06|HLC-12064|652[0n]bp
Halichoeres leucoxanthus|TZAIB142-06|HLC-12065|652[0n]bp
Halichoeres sp.|TZAIC517-06|HLC-11585|652[0n]bp
Macropharyngodon meleagris|TZAIB862-07|HLC-15192|647[0n]bp
Macropharyngodon meleagris|TZAIC393-05|HLC-11101|648[0n]bp
Macropharyngodon meleagris|TZAIC630-06|HLC-11698|648[1n]bp
Macropharyngodon meleagris|TZAIC631-06|HLC-11699|648[0n]bp
Macropharyngodon meleagris|TZAIC632-06|HLC-11700|648[0n]bp
Macropharyngodon meleagris|TZAIC471-06|HLC-11539|648[0n]bp
Macropharyngodon meleagris|TZAIB729-06|HLC-15059|648[0n]bp
Macropharyngodon meleagris|TZAIC391-05|HLC-11099|648[0n]bp
Macropharyngodon meleagris|TZAIC392-05|HLC-11100|648[0n]bp
Macropharyngodon meleagris|TZAIC394-05|HLC-11102|648[0n]bp
Macropharyngodon meleagris|TZAIC270-05|HLC-10970|648[0n]bp
Macropharyngodon meleagris|TZAIC778-06|HLC-11840|652[0n]bp
Macropharyngodon meleagris|TZAIC779-06|HLC-11841|652[0n]bp
Macropharyngodon meleagris|TZAIC271-05|HLC-10971|587[0n]bp
Macropharyngodon meleagris|TZAIC628-06|HLC-11696|648[0n]bp
Macropharyngodon meleagris|TZAIC629-06|HLC-11697|648[0n]bp
Macropharyngodon ornatus|TZAIC355-05|HLC-11063|648[0n]bp
Labrichthys unilineatus|TZAIB898-07|HLC-15228|648[0n]bp
Labroides dimidiatus|TZAIC254-05|HLC-10954|648[0n]bp
Labroides dimidiatus|TZAIC536-06|HLC-11604|612[0n]bp
Labroides dimidiatus|TZAIC261-05|HLC-10961|652[0n]bp
Labroides dimidiatus|TZAIC852-06|HLC-12211|652[0n]bp
Labroides dimidiatus|TZAIC258-05|HLC-10958|652[0n]bp
Labroides dimidiatus|TZAIC259-05|HLC-10959|652[0n]bp
Labroides dimidiatus|TZAIC141-05|HLC-10841|652[0n]bp
Labroides dimidiatus|TZAIC851-06|HLC-12210|609[5n]bp
Labroides dimidiatus|TZAIC140-05|HLC-10840|652[0n]bp
Labroides dimidiatus|TZAIC257-05|HLC-10957|652[0n]bp
Labroides dimidiatus|TZAIC255-05|HLC-10955|652[2n]bp
Labroides dimidiatus|TZAIC252-05|HLC-10952|621[0n]bp
Labroides dimidiatus|TZAIC256-05|HLC-10956|652[0n]bp
Labroides dimidiatus|TZAIC253-05|HLC-10953|611[1n]bp
Labroides dimidiatus|TZAIC260-05|HLC-10960|652[0n]bp
Labroides dimidiatus|TZAIC535-06|HLC-11603|652[0n]bp
Labroides dimidiatus|TZAIC537-06|HLC-11605|652[0n]bp
Coris formosa|TZAIB878-07|HLC-15208|652[0n]bp
Coris formosa|TZAIC714-06|HLC-11776|652[0n]bp
Coris formosa|TZAIC829-06|HLC-11891|652[4n]bp
Coris gaimard|TZAIC235-05|HLC-10935|652[0n]bp
Coris gaimard|TZAIC234-05|HLC-10934|652[0n]bp
Coris gaimard|TZAIB149-06|HLC-12072|652[0n]bp
Coris gaimard|TZAIC362-05|HLC-11070|652[0n]bp

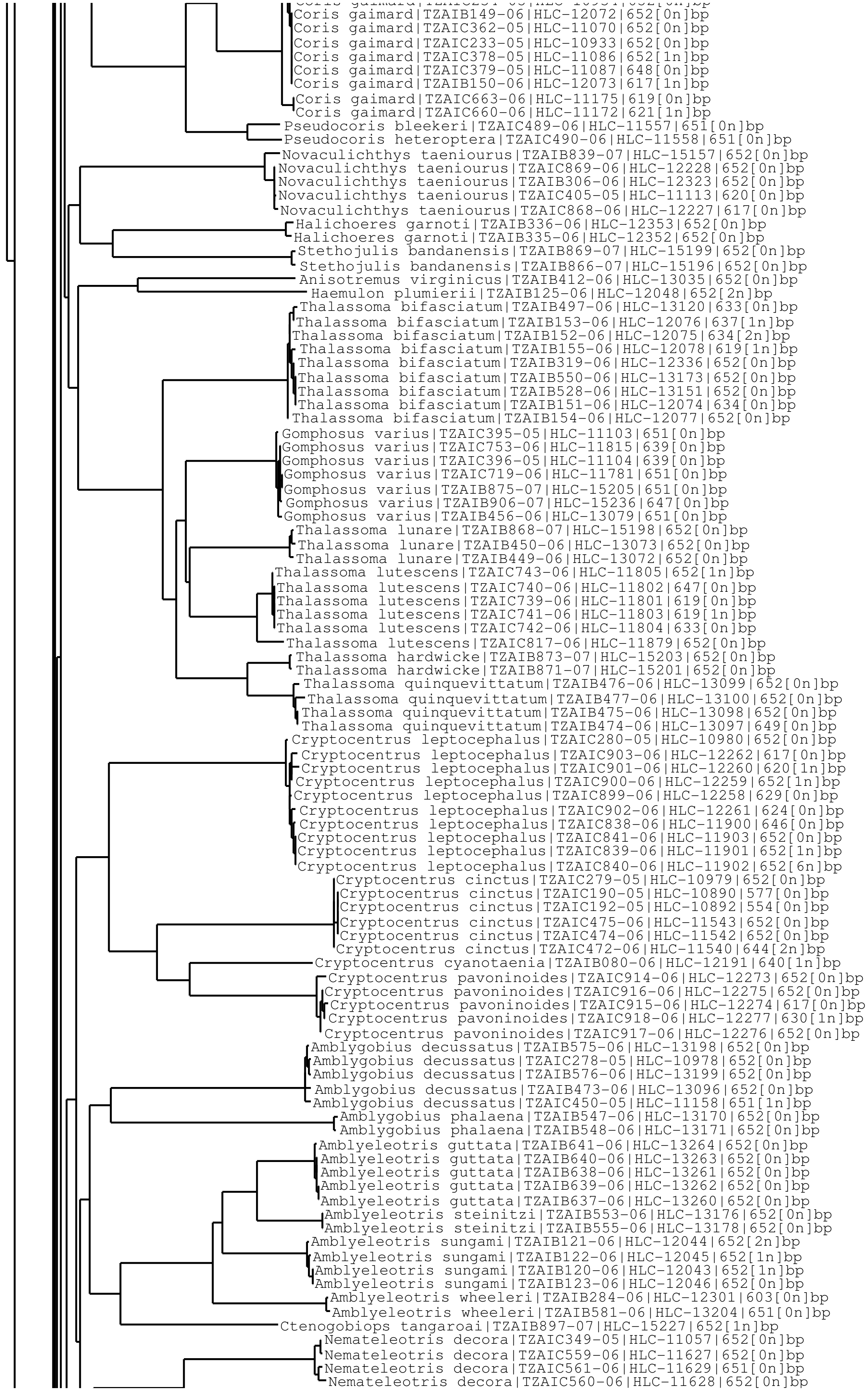
Coris gaimard|TZAIB149-06|HLC-12072|652[0n]bp
Coris gaimard|TZAIC362-05|HLC-11070|652[0n]bp
Coris gaimard|TZAIC233-05|HLC-10933|652[0n]bp
Coris gaimard|TZAIC378-05|HLC-11086|652[1n]bp
Coris gaimard|TZAIC379-05|HLC-11087|648[0n]bp
Coris gaimard|TZAIB150-06|HLC-12073|617[1n]bp
Coris gaimard|TZAIC663-06|HLC-11175|619[0n]bp
Coris gaimard|TZAIC660-06|HLC-11172|621[1n]bp
Pseudocoris bleekeri|TZAIC489-06|HLC-11557|651[0n]bp
Pseudocoris heteroptera|TZAIC490-06|HLC-11558|651[0n]bp
Novaculichthys taeniourus|TZAIB839-07|HLC-15157|652[0n]bp
Novaculichthys taeniourus|TZAIC869-06|HLC-12228|652[0n]bp
Novaculichthys taeniourus|TZAIB306-06|HLC-12323|652[0n]bp
Novaculichthys taeniourus|TZAIC405-05|HLC-11113|620[0n]bp
Novaculichthys taeniourus|TZAIC868-06|HLC-12227|617[0n]bp
Halichoeres garnoti|TZAIB336-06|HLC-12353|652[0n]bp
Halichoeres garnoti|TZAIB335-06|HLC-12352|652[0n]bp
Stethojulis bandanensis|TZAIB869-07|HLC-15199|652[0n]bp
Stethojulis bandanensis|TZAIB866-07|HLC-15196|652[0n]bp
Anisotremus virginicus|TZAIB412-06|HLC-13035|652[0n]bp
Haemulon plumierii|TZAIB125-06|HLC-12048|652[2n]bp
Thalassoma bifasciatum|TZAIB497-06|HLC-13120|633[0n]bp
Thalassoma bifasciatum|TZAIB153-06|HLC-12076|637[1n]bp
Thalassoma bifasciatum|TZAIB152-06|HLC-12075|634[2n]bp
Thalassoma bifasciatum|TZAIB155-06|HLC-12078|619[1n]bp
Thalassoma bifasciatum|TZAIB319-06|HLC-12336|652[0n]bp
Thalassoma bifasciatum|TZAIB550-06|HLC-13173|652[0n]bp
Thalassoma bifasciatum|TZAIB528-06|HLC-13151|652[0n]bp
Thalassoma bifasciatum|TZAIB151-06|HLC-12074|634[0n]bp
Thalassoma bifasciatum|TZAIB154-06|HLC-12077|652[0n]bp
Gomphosus varius|TZAIC395-05|HLC-11103|651[0n]bp
Gomphosus varius|TZAIC753-06|HLC-11815|639[0n]bp
Gomphosus varius|TZAIC396-05|HLC-11104|639[0n]bp
Gomphosus varius|TZAIC719-06|HLC-11781|651[0n]bp
Gomphosus varius|TZAIB875-07|HLC-15205|651[0n]bp
Gomphosus varius|TZAIB906-07|HLC-15236|647[0n]bp
Gomphosus varius|TZAIB456-06|HLC-13079|651[0n]bp
Thalassoma lunare|TZAIB868-07|HLC-15198|652[0n]bp
Thalassoma lunare|TZAIB450-06|HLC-13073|652[0n]bp
Thalassoma lunare|TZAIB449-06|HLC-13072|652[0n]bp
Thalassoma lutescens|TZAIC743-06|HLC-11805|652[1n]bp
Thalassoma lutescens|TZAIC740-06|HLC-11802|647[0n]bp
Thalassoma lutescens|TZAIC739-06|HLC-11801|619[0n]bp
Thalassoma lutescens|TZAIC741-06|HLC-11803|619[1n]bp
Thalassoma lutescens|TZAIC742-06|HLC-11804|633[0n]bp
Thalassoma lutescens|TZAIC817-06|HLC-11879|652[0n]bp
Thalassoma hardwicke|TZAIB873-07|HLC-15203|652[0n]bp
Thalassoma hardwicke|TZAIB871-07|HLC-15201|652[0n]bp
Thalassoma quinquevittatum|TZAIB476-06|HLC-13099|652[0n]bp
Thalassoma quinquevittatum|TZAIB477-06|HLC-13100|652[0n]bp
Thalassoma quinquevittatum|TZAIB475-06|HLC-13098|652[0n]bp
Thalassoma quinquevittatum|TZAIB474-06|HLC-13097|649[0n]bp
Cryptocentrus leptocephalus|TZAIC280-05|HLC-10980|652[0n]bp
Cryptocentrus leptocephalus|TZAIC903-06|HLC-12262|617[0n]bp
Cryptocentrus leptocephalus|TZAIC901-06|HLC-12260|620[1n]bp
Cryptocentrus leptocephalus|TZAIC900-06|HLC-12259|652[1n]bp
Cryptocentrus leptocephalus|TZAIC899-06|HLC-12258|629[0n]bp
Cryptocentrus leptocephalus|TZAIC902-06|HLC-12261|624[0n]bp
Cryptocentrus leptocephalus|TZAIC838-06|HLC-11900|646[0n]bp
Cryptocentrus leptocephalus|TZAIC841-06|HLC-11903|652[0n]bp
Cryptocentrus leptocephalus|TZAIC839-06|HLC-11901|652[1n]bp
Cryptocentrus leptocephalus|TZAIC840-06|HLC-11902|652[6n]bp
Cryptocentrus cinctus|TZAIC279-05|HLC-10979|652[0n]bp
Cryptocentrus cinctus|TZAIC190-05|HLC-10890|577[0n]bp
Cryptocentrus cinctus|TZAIC192-05|HLC-10892|554[0n]bp
Cryptocentrus cinctus|TZAIC475-06|HLC-11543|652[0n]bp
Cryptocentrus cinctus|TZAIC474-06|HLC-11542|652[0n]bp
Cryptocentrus cinctus|TZAIC472-06|HLC-11540|644[2n]bp
Cryptocentrus cyanotaenia|TZAIB080-06|HLC-12191|640[1n]bp
Cryptocentrus pavoninoides|TZAIC914-06|HLC-12273|652[0n]bp
Cryptocentrus pavoninoides|TZAIC916-06|HLC-12275|652[0n]bp
Cryptocentrus pavoninoides|TZAIC915-06|HLC-12274|617[0n]bp
Cryptocentrus pavoninoides|TZAIC918-06|HLC-12277|630[1n]bp
Cryptocentrus pavoninoides|TZAIC917-06|HLC-12276|652[0n]bp
Amblygobius decussatus|TZAIB575-06|HLC-13198|652[0n]bp
Amblygobius decussatus|TZAIC278-05|HLC-10978|652[0n]bp
Amblygobius decussatus|TZAIB576-06|HLC-13199|652[0n]bp
Amblygobius decussatus|TZAIB473-06|HLC-13096|652[0n]bp
Amblygobius decussatus|TZAIC450-05|HLC-11158|651[1n]bp
Amblygobius phalaena|TZAIB547-06|HLC-13170|652[0n]bp
Amblygobius phalaena|TZAIB548-06|HLC-13171|652[0n]bp
Amblyeleotris guttata|TZAIB641-06|HLC-13264|652[0n]bp
Amblyeleotris guttata|TZAIB640-06|HLC-13263|652[0n]bp
Amblyeleotris guttata|TZAIB638-06|HLC-13261|652[0n]bp
Amblyeleotris guttata|TZAIB639-06|HLC-13262|652[0n]bp
Amblyeleotris guttata|TZAIB637-06|HLC-13260|652[0n]bp
Amblyeleotris steinitzi|TZAIB553-06|HLC-13176|652[0n]bp
Amblyeleotris steinitzi|TZAIB555-06|HLC-13178|652[0n]bp
Amblyeleotris sungami|TZAIB121-06|HLC-12044|652[2n]bp
Amblyeleotris sungami|TZAIB122-06|HLC-12045|652[1n]bp
Amblyeleotris sungami|TZAIB120-06|HLC-12043|652[1n]bp
Amblyeleotris sungami|TZAIB123-06|HLC-12046|652[0n]bp
Amblyeleotris wheeleri|TZAIB284-06|HLC-12301|603[0n]bp
Amblyeleotris wheeleri|TZAIB581-06|HLC-13204|651[0n]bp
Ctenogobiops tangaroai|TZAIB897-07|HLC-15227|652[1n]bp
Nemateleotris decora|TZAIC349-05|HLC-11057|652[0n]bp
Nemateleotris decora|TZAIC559-06|HLC-11627|652[0n]bp
Nemateleotris decora|TZAIC561-06|HLC-11629|651[0n]bp
Nemateleotris decora|TZAIC560-06|HLC-11628|652[0n]bp

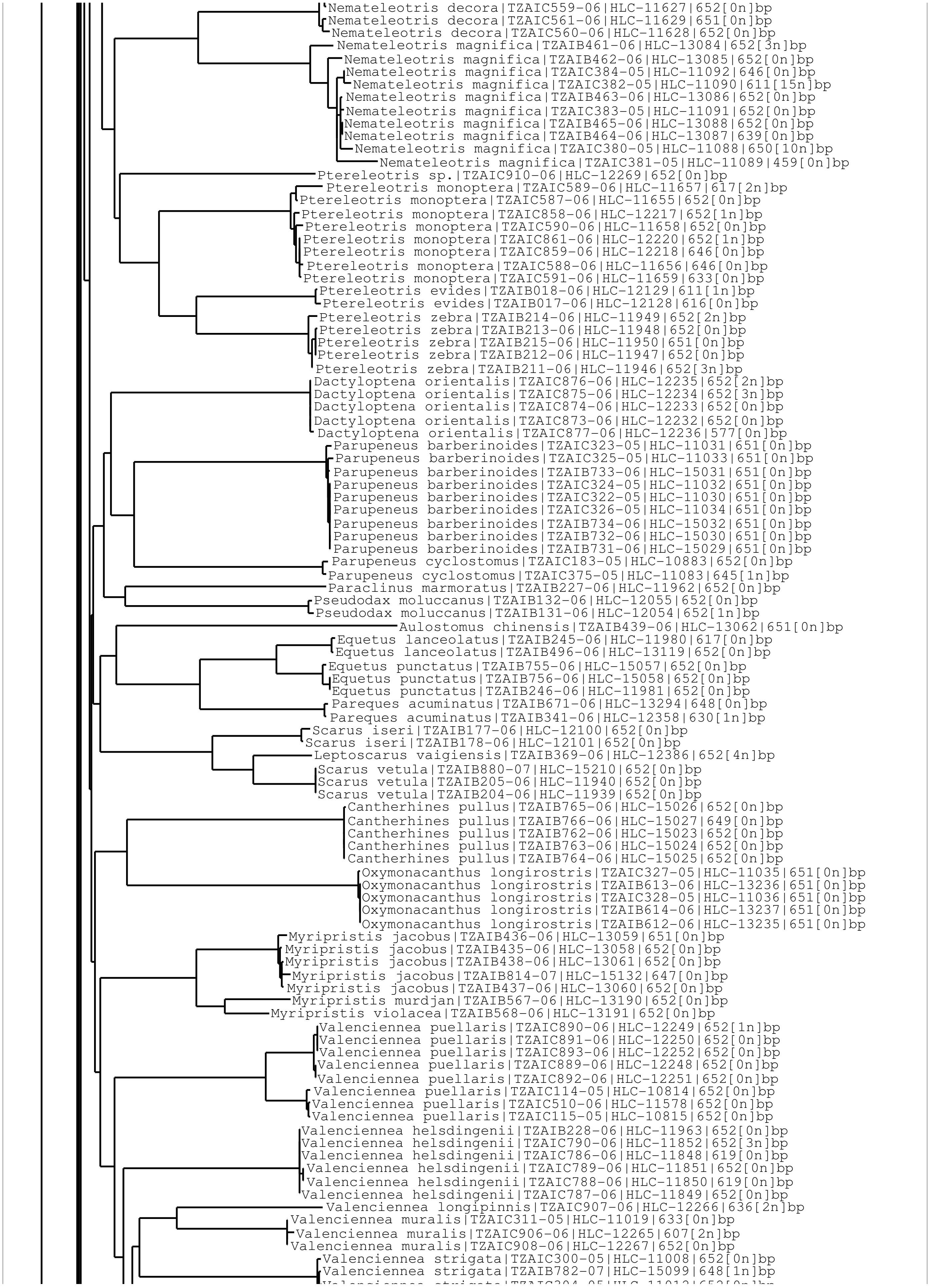
Nemateleotris decora|TZAIC559-06|HLC-11627|652[0n]bp
Nemateleotris decora|TZAIC561-06|HLC-11629|651[0n]bp
Nemateleotris decora|TZAIC560-06|HLC-11628|652[0n]bp
Nemateleotris magnifica|TZAIB461-06|HLC-13084|652[3n]bp
Nemateleotris magnifica|TZAIB462-06|HLC-13085|652[0n]bp
Nemateleotris magnifica|TZAIC384-05|HLC-11092|646[0n]bp
Nemateleotris magnifica|TZAIC382-05|HLC-11090|611[15n]bp
Nemateleotris magnifica|TZAIB463-06|HLC-13086|652[0n]bp
Nemateleotris magnifica|TZAIC383-05|HLC-11091|652[0n]bp
Nemateleotris magnifica|TZAIB465-06|HLC-13088|652[0n]bp
Nemateleotris magnifica|TZAIB464-06|HLC-13087|639[0n]bp
Nemateleotris magnifica|TZAIC380-05|HLC-11088|650[10n]bp
Nemateleotris magnifica|TZAIC381-05|HLC-11089|459[0n]bp
Ptereleotris sp.|TZAIC910-06|HLC-12269|652[0n]bp
Ptereleotris monoptera|TZAIC589-06|HLC-11657|617[2n]bp
Ptereleotris monoptera|TZAIC587-06|HLC-11655|652[0n]bp
Ptereleotris monoptera|TZAIC858-06|HLC-12217|652[1n]bp
Ptereleotris monoptera|TZAIC590-06|HLC-11658|652[0n]bp
Ptereleotris monoptera|TZAIC861-06|HLC-12220|652[1n]bp
Ptereleotris monoptera|TZAIC859-06|HLC-12218|646[0n]bp
Ptereleotris monoptera|TZAIC588-06|HLC-11656|646[0n]bp
Ptereleotris monoptera|TZAIC591-06|HLC-11659|633[0n]bp
Ptereleotris evides|TZAIB018-06|HLC-12129|611[1n]bp
Ptereleotris evides|TZAIB017-06|HLC-12128|616[0n]bp
Ptereleotris zebra|TZAIB214-06|HLC-11949|652[2n]bp
Ptereleotris zebra|TZAIB213-06|HLC-11948|652[0n]bp
Ptereleotris zebra|TZAIB215-06|HLC-11950|651[0n]bp
Ptereleotris zebra|TZAIB212-06|HLC-11947|652[0n]bp
Ptereleotris zebra|TZAIB211-06|HLC-11946|652[3n]bp
Dactyloptena orientalis|TZAIC876-06|HLC-12235|652[2n]bp
Dactyloptena orientalis|TZAIC875-06|HLC-12234|652[3n]bp
Dactyloptena orientalis|TZAIC874-06|HLC-12233|652[0n]bp
Dactyloptena orientalis|TZAIC873-06|HLC-12232|652[0n]bp
Dactyloptena orientalis|TZAIC877-06|HLC-12236|577[0n]bp
Parupeneus barberinoides|TZAIC323-05|HLC-11031|651[0n]bp
Parupeneus barberinoides|TZAIC325-05|HLC-11033|651[0n]bp
Parupeneus barberinoides|TZAIB733-06|HLC-15031|651[0n]bp
Parupeneus barberinoides|TZAIC324-05|HLC-11032|651[0n]bp
Parupeneus barberinoides|TZAIC322-05|HLC-11030|651[0n]bp
Parupeneus barberinoides|TZAIC326-05|HLC-11034|651[0n]bp
Parupeneus barberinoides|TZAIB734-06|HLC-15032|651[0n]bp
Parupeneus barberinoides|TZAIB732-06|HLC-15030|651[0n]bp
Parupeneus barberinoides|TZAIB731-06|HLC-15029|651[0n]bp
Parupeneus cyclostomus|TZAIC183-05|HLC-10883|652[0n]bp
Parupeneus cyclostomus|TZAIC375-05|HLC-11083|645[1n]bp
Paraclinus marmoratus|TZAIB227-06|HLC-11962|652[0n]bp
Pseudodax moluccanus|TZAIB132-06|HLC-12055|652[0n]bp
Pseudodax moluccanus|TZAIB131-06|HLC-12054|652[1n]bp
Aulostomus chinensis|TZAIB439-06|HLC-13062|651[0n]bp
Equetus lanceolatus|TZAIB245-06|HLC-11980|617[0n]bp
Equetus lanceolatus|TZAIB496-06|HLC-13119|652[0n]bp
Equetus punctatus|TZAIB755-06|HLC-15057|652[0n]bp
Equetus punctatus|TZAIB756-06|HLC-15058|652[0n]bp
Equetus punctatus|TZAIB246-06|HLC-11981|652[0n]bp
Pareques acuminatus|TZAIB671-06|HLC-13294|648[0n]bp
Pareques acuminatus|TZAIB341-06|HLC-12358|630[1n]bp
Scarus iseri|TZAIB177-06|HLC-12100|652[0n]bp
Scarus iseri|TZAIB178-06|HLC-12101|652[0n]bp
Leptoscarus vaigiensis|TZAIB369-06|HLC-12386|652[4n]bp
Scarus vetula|TZAIB880-07|HLC-15210|652[0n]bp
Scarus vetula|TZAIB205-06|HLC-11940|652[0n]bp
Scarus vetula|TZAIB204-06|HLC-11939|652[0n]bp
Cantherhines pullus|TZAIB765-06|HLC-15026|652[0n]bp
Cantherhines pullus|TZAIB766-06|HLC-15027|649[0n]bp
Cantherhines pullus|TZAIB762-06|HLC-15023|652[0n]bp
Cantherhines pullus|TZAIB763-06|HLC-15024|652[0n]bp
Cantherhines pullus|TZAIB764-06|HLC-15025|652[0n]bp
Oxymonacanthus longirostris|TZAIC327-05|HLC-11035|651[0n]bp
Oxymonacanthus longirostris|TZAIB613-06|HLC-13236|651[0n]bp
Oxymonacanthus longirostris|TZAIC328-05|HLC-11036|651[0n]bp
Oxymonacanthus longirostris|TZAIB614-06|HLC-13237|651[0n]bp
Oxymonacanthus longirostris|TZAIB612-06|HLC-13235|651[0n]bp
Myripristis jacobus|TZAIB436-06|HLC-13059|651[0n]bp
Myripristis jacobus|TZAIB435-06|HLC-13058|652[0n]bp
Myripristis jacobus|TZAIB438-06|HLC-13061|652[0n]bp
Myripristis jacobus|TZAIB814-07|HLC-15132|647[0n]bp
Myripristis jacobus|TZAIB437-06|HLC-13060|652[0n]bp
Myripristis murdjan|TZAIB567-06|HLC-13190|652[0n]bp
Myripristis violacea|TZAIB568-06|HLC-13191|652[0n]bp
Valenciennea puellaris|TZAIC890-06|HLC-12249|652[1n]bp
Valenciennea puellaris|TZAIC891-06|HLC-12250|652[0n]bp
Valenciennea puellaris|TZAIC893-06|HLC-12252|652[0n]bp
Valenciennea puellaris|TZAIC889-06|HLC-12248|652[0n]bp
Valenciennea puellaris|TZAIC892-06|HLC-12251|652[0n]bp
Valenciennea puellaris|TZAIC114-05|HLC-10814|652[0n]bp
Valenciennea puellaris|TZAIC510-06|HLC-11578|652[0n]bp
Valenciennea puellaris|TZAIC115-05|HLC-10815|652[0n]bp
Valenciennea helsdingenii|TZAIB228-06|HLC-11963|652[0n]bp
Valenciennea helsdingenii|TZAIC790-06|HLC-11852|652[3n]bp
Valenciennea helsdingenii|TZAIC786-06|HLC-11848|619[0n]bp
Valenciennea helsdingenii|TZAIC789-06|HLC-11851|652[0n]bp
Valenciennea helsdingenii|TZAIC788-06|HLC-11850|619[0n]bp
Valenciennea helsdingenii|TZAIC787-06|HLC-11849|652[0n]bp
Valenciennea longipinnis|TZAIC907-06|HLC-12266|636[2n]bp
Valenciennea muralis|TZAIC311-05|HLC-11019|633[0n]bp
Valenciennea muralis|TZAIC906-06|HLC-12265|607[2n]bp
Valenciennea muralis|TZAIC908-06|HLC-12267|652[0n]bp
Valenciennea strigata|TZAIC300-05|HLC-11008|652[0n]bp
Valenciennea strigata|TZAIB782-07|HLC-15099|648[1n]bp

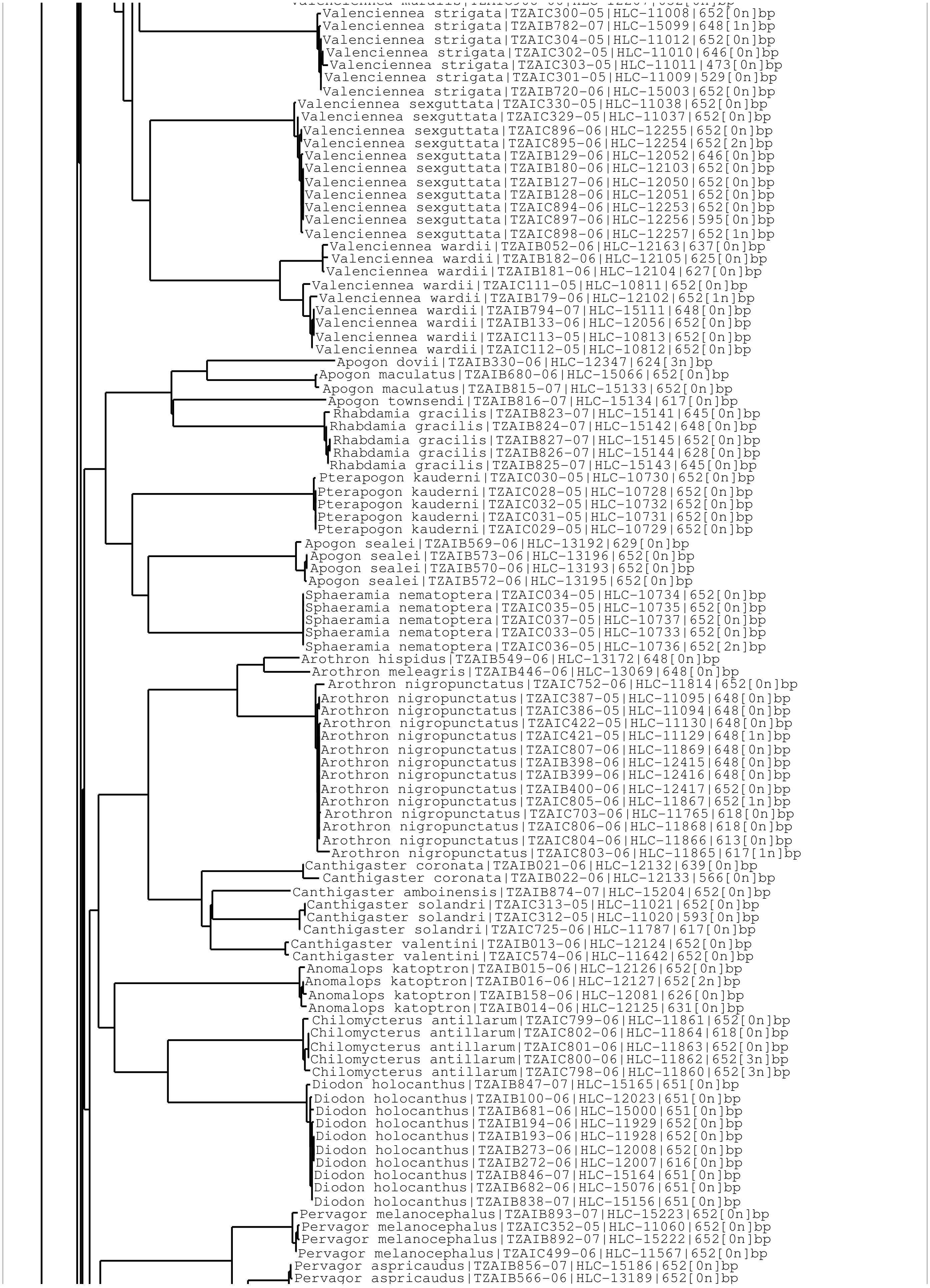
Valenciennea strigata|TZAIC300-05|HLC-11008|652[0n]bp
Valenciennea strigata|TZAIB782-07|HLC-15099|648[1n]bp
Valenciennea strigata|TZAIC304-05|HLC-11012|652[0n]bp
Valenciennea strigata|TZAIC302-05|HLC-11010|646[0n]bp
Valenciennea strigata|TZAIC303-05|HLC-11011|473[0n]bp
Valenciennea strigata|TZAIC301-05|HLC-11009|529[0n]bp
Valenciennea strigata|TZAIB720-06|HLC-15003|652[0n]bp
Valenciennea sexguttata|TZAIC330-05|HLC-11038|652[0n]bp
Valenciennea sexguttata|TZAIC329-05|HLC-11037|652[0n]bp
Valenciennea sexguttata|TZAIC896-06|HLC-12255|652[0n]bp
Valenciennea sexguttata|TZAIC895-06|HLC-12254|652[2n]bp
Valenciennea sexguttata|TZAIB129-06|HLC-12052|646[0n]bp
Valenciennea sexguttata|TZAIB180-06|HLC-12103|652[0n]bp
Valenciennea sexguttata|TZAIB127-06|HLC-12050|652[0n]bp
Valenciennea sexguttata|TZAIB128-06|HLC-12051|652[0n]bp
Valenciennea sexguttata|TZAIC894-06|HLC-12253|652[0n]bp
Valenciennea sexguttata|TZAIC897-06|HLC-12256|595[0n]bp
Valenciennea sexguttata|TZAIC898-06|HLC-12257|652[1n]bp
Valenciennea wardii|TZAIB052-06|HLC-12163|637[0n]bp
Valenciennea wardii|TZAIB182-06|HLC-12105|625[0n]bp
Valenciennea wardii|TZAIB181-06|HLC-12104|627[0n]bp
Valenciennea wardii|TZAIC111-05|HLC-10811|652[0n]bp
Valenciennea wardii|TZAIB179-06|HLC-12102|652[1n]bp
Valenciennea wardii|TZAIB794-07|HLC-15111|648[0n]bp
Valenciennea wardii|TZAIB133-06|HLC-12056|652[0n]bp
Valenciennea wardii|TZAIC113-05|HLC-10813|652[0n]bp
Valenciennea wardii|TZAIC112-05|HLC-10812|652[0n]bp
Apogon dovii|TZAIB330-06|HLC-12347|624[3n]bp
Apogon maculatus|TZAIB680-06|HLC-15066|652[0n]bp
Apogon maculatus|TZAIB815-07|HLC-15133|652[0n]bp
Apogon townsendi|TZAIB816-07|HLC-15134|617[0n]bp
Rhabdamia gracilis|TZAIB823-07|HLC-15141|645[0n]bp
Rhabdamia gracilis|TZAIB824-07|HLC-15142|648[0n]bp
Rhabdamia gracilis|TZAIB827-07|HLC-15145|652[0n]bp
Rhabdamia gracilis|TZAIB826-07|HLC-15144|628[0n]bp
Rhabdamia gracilis|TZAIB825-07|HLC-15143|645[0n]bp
Pterapogon kauderni|TZAIC030-05|HLC-10730|652[0n]bp
Pterapogon kauderni|TZAIC028-05|HLC-10728|652[0n]bp
Pterapogon kauderni|TZAIC032-05|HLC-10732|652[0n]bp
Pterapogon kauderni|TZAIC031-05|HLC-10731|652[0n]bp
Pterapogon kauderni|TZAIC029-05|HLC-10729|652[0n]bp
Apogon sealei|TZAIB569-06|HLC-13192|629[0n]bp
Apogon sealei|TZAIB573-06|HLC-13196|652[0n]bp
Apogon sealei|TZAIB570-06|HLC-13193|652[0n]bp
Apogon sealei|TZAIB572-06|HLC-13195|652[0n]bp
Sphaeramia nematoptera|TZAIC034-05|HLC-10734|652[0n]bp
Sphaeramia nematoptera|TZAIC035-05|HLC-10735|652[0n]bp
Sphaeramia nematoptera|TZAIC037-05|HLC-10737|652[0n]bp
Sphaeramia nematoptera|TZAIC033-05|HLC-10733|652[0n]bp
Sphaeramia nematoptera|TZAIC036-05|HLC-10736|652[2n]bp
Arothron hispidus|TZAIB549-06|HLC-13172|648[0n]bp
Arothron meleagris|TZAIB446-06|HLC-13069|648[0n]bp
Arothron nigropunctatus|TZAIC752-06|HLC-11814|652[0n]bp
Arothron nigropunctatus|TZAIC387-05|HLC-11095|648[0n]bp
Arothron nigropunctatus|TZAIC386-05|HLC-11094|648[0n]bp
Arothron nigropunctatus|TZAIC422-05|HLC-11130|648[0n]bp
Arothron nigropunctatus|TZAIC421-05|HLC-11129|648[1n]bp
Arothron nigropunctatus|TZAIC807-06|HLC-11869|648[0n]bp
Arothron nigropunctatus|TZAIB398-06|HLC-12415|648[0n]bp
Arothron nigropunctatus|TZAIB399-06|HLC-12416|648[0n]bp
Arothron nigropunctatus|TZAIB400-06|HLC-12417|652[0n]bp
Arothron nigropunctatus|TZAIC805-06|HLC-11867|652[1n]bp
Arothron nigropunctatus|TZAIC703-06|HLC-11765|618[0n]bp
Arothron nigropunctatus|TZAIC806-06|HLC-11868|618[0n]bp
Arothron nigropunctatus|TZAIC804-06|HLC-11866|613[0n]bp
Arothron nigropunctatus|TZAIC803-06|HLC-11865|617[1n]bp
Canthigaster coronata|TZAIB021-06|HLC-12132|639[0n]bp
Canthigaster coronata|TZAIB022-06|HLC-12133|566[0n]bp
Canthigaster amboinensis|TZAIB874-07|HLC-15204|652[0n]bp
Canthigaster solandri|TZAIC313-05|HLC-11021|652[0n]bp
Canthigaster solandri|TZAIC312-05|HLC-11020|593[0n]bp
Canthigaster solandri|TZAIC725-06|HLC-11787|617[0n]bp
Canthigaster valentini|TZAIB013-06|HLC-12124|652[0n]bp
Canthigaster valentini|TZAIC574-06|HLC-11642|652[0n]bp
Anomalops katoptron|TZAIB015-06|HLC-12126|652[0n]bp
Anomalops katoptron|TZAIB016-06|HLC-12127|652[2n]bp
Anomalops katoptron|TZAIB158-06|HLC-12081|626[0n]bp
Anomalops katoptron|TZAIB014-06|HLC-12125|631[0n]bp
Chilomycterus antillarum|TZAIC799-06|HLC-11861|652[0n]bp
Chilomycterus antillarum|TZAIC802-06|HLC-11864|618[0n]bp
Chilomycterus antillarum|TZAIC801-06|HLC-11863|652[0n]bp
Chilomycterus antillarum|TZAIC800-06|HLC-11862|652[3n]bp
Chilomycterus antillarum|TZAIC798-06|HLC-11860|652[3n]bp
Diodon holocanthus|TZAIB847-07|HLC-15165|651[0n]bp
Diodon holocanthus|TZAIB100-06|HLC-12023|651[0n]bp
Diodon holocanthus|TZAIB681-06|HLC-15000|651[0n]bp
Diodon holocanthus|TZAIB194-06|HLC-11929|652[0n]bp
Diodon holocanthus|TZAIB193-06|HLC-11928|652[0n]bp
Diodon holocanthus|TZAIB273-06|HLC-12008|652[0n]bp
Diodon holocanthus|TZAIB272-06|HLC-12007|616[0n]bp
Diodon holocanthus|TZAIB846-07|HLC-15164|651[0n]bp
Diodon holocanthus|TZAIB682-06|HLC-15076|651[0n]bp
Diodon holocanthus|TZAIB838-07|HLC-15156|651[0n]bp
Pervagor melanocephalus|TZAIB893-07|HLC-15223|652[0n]bp
Pervagor melanocephalus|TZAIC352-05|HLC-11060|652[0n]bp
Pervagor melanocephalus|TZAIB892-07|HLC-15222|652[0n]bp
Pervagor melanocephalus|TZAIC499-06|HLC-11567|652[0n]bp
Pervagor aspricaudus|TZAIB856-07|HLC-15186|652[0n]bp
Pervagor aspricaudus|TZAIB566-06|HLC-13189|652[0n]bp

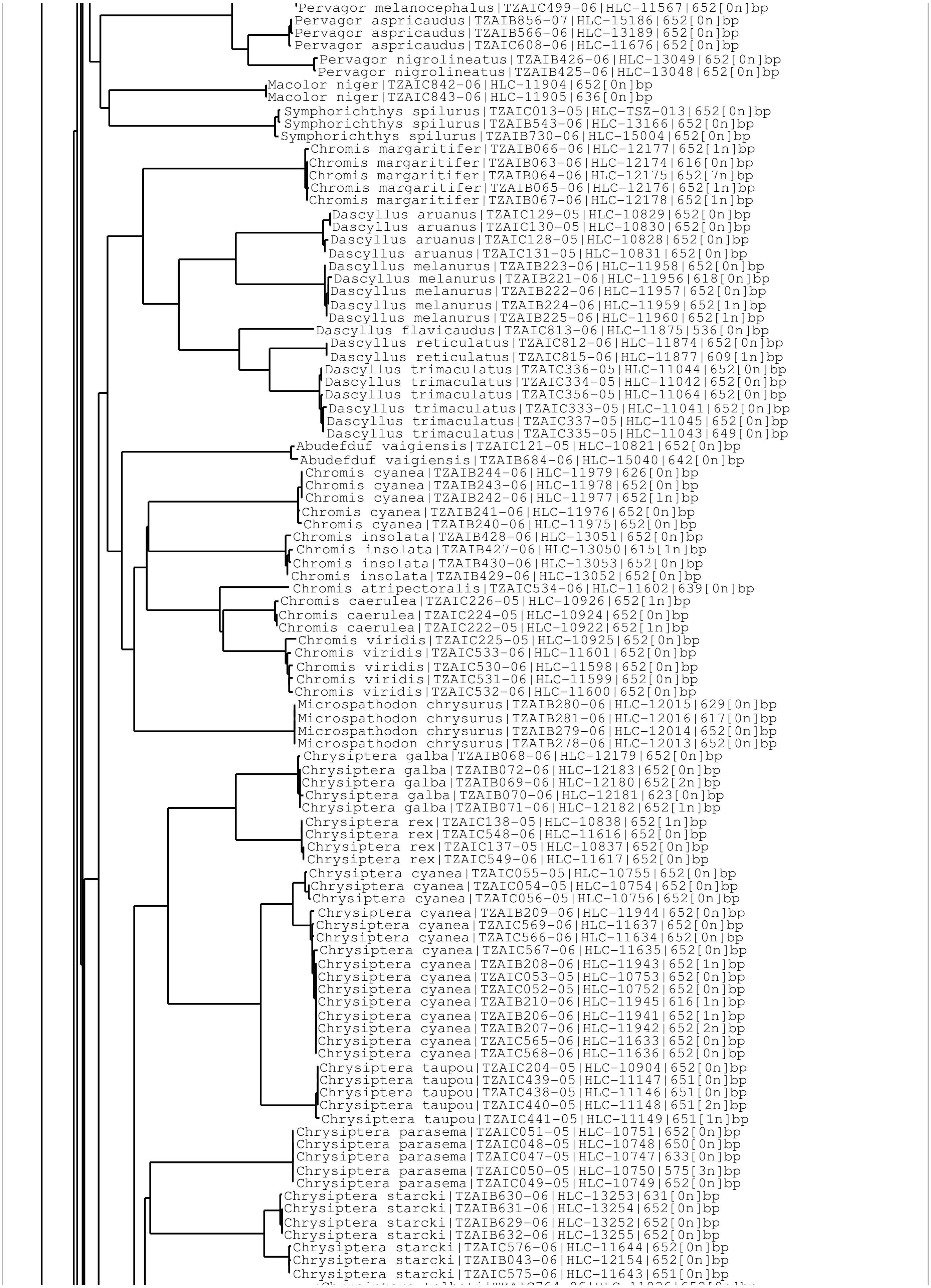
Pervagor melanocephalus|TZAIC499-06|HLC-11567|652[0n]bp
Pervagor aspricaudus|TZAIB856-07|HLC-15186|652[0n]bp
Pervagor aspricaudus|TZAIB566-06|HLC-13189|652[0n]bp
Pervagor aspricaudus|TZAIC608-06|HLC-11676|652[0n]bp
Pervagor nigrolineatus|TZAIB426-06|HLC-13049|652[0n]bp
Pervagor nigrolineatus|TZAIB425-06|HLC-13048|652[0n]bp
Macolor niger|TZAIC842-06|HLC-11904|652[0n]bp
Macolor niger|TZAIC843-06|HLC-11905|636[0n]bp
Symphorichthys spilurus|TZAIC013-05|HLC-TSZ-013|652[0n]bp
Symphorichthys spilurus|TZAIB543-06|HLC-13166|652[0n]bp
Symphorichthys spilurus|TZAIB730-06|HLC-15004|652[0n]bp
Chromis margaritifer|TZAIB066-06|HLC-12177|652[1n]bp
Chromis margaritifer|TZAIB063-06|HLC-12174|616[0n]bp
Chromis margaritifer|TZAIB064-06|HLC-12175|652[7n]bp
Chromis margaritifer|TZAIB065-06|HLC-12176|652[1n]bp
Chromis margaritifer|TZAIB067-06|HLC-12178|652[1n]bp
Dascyllus aruanus|TZAIC129-05|HLC-10829|652[0n]bp
Dascyllus aruanus|TZAIC130-05|HLC-10830|652[0n]bp
Dascyllus aruanus|TZAIC128-05|HLC-10828|652[0n]bp
Dascyllus aruanus|TZAIC131-05|HLC-10831|652[0n]bp
Dascyllus melanurus|TZAIB223-06|HLC-11958|652[0n]bp
Dascyllus melanurus|TZAIB221-06|HLC-11956|618[0n]bp
Dascyllus melanurus|TZAIB222-06|HLC-11957|652[0n]bp
Dascyllus melanurus|TZAIB224-06|HLC-11959|652[1n]bp
Dascyllus melanurus|TZAIB225-06|HLC-11960|652[1n]bp
Dascyllus flavicaudus|TZAIC813-06|HLC-11875|536[0n]bp
Dascyllus reticulatus|TZAIC812-06|HLC-11874|652[0n]bp
Dascyllus reticulatus|TZAIC815-06|HLC-11877|609[1n]bp
Dascyllus trimaculatus|TZAIC336-05|HLC-11044|652[0n]bp
Dascyllus trimaculatus|TZAIC334-05|HLC-11042|652[0n]bp
Dascyllus trimaculatus|TZAIC356-05|HLC-11064|652[0n]bp
Dascyllus trimaculatus|TZAIC333-05|HLC-11041|652[0n]bp
Dascyllus trimaculatus|TZAIC337-05|HLC-11045|652[0n]bp
Dascyllus trimaculatus|TZAIC335-05|HLC-11043|649[0n]bp
Abudefduf vaigiensis|TZAIC121-05|HLC-10821|652[0n]bp
Abudefduf vaigiensis|TZAIB684-06|HLC-15040|642[0n]bp
Chromis cyanea|TZAIB244-06|HLC-11979|626[0n]bp
Chromis cyanea|TZAIB243-06|HLC-11978|652[0n]bp
Chromis cyanea|TZAIB242-06|HLC-11977|652[1n]bp
Chromis cyanea|TZAIB241-06|HLC-11976|652[0n]bp
Chromis cyanea|TZAIB240-06|HLC-11975|652[0n]bp
Chromis insolata|TZAIB428-06|HLC-13051|652[0n]bp
Chromis insolata|TZAIB427-06|HLC-13050|615[1n]bp
Chromis insolata|TZAIB430-06|HLC-13053|652[0n]bp
Chromis insolata|TZAIB429-06|HLC-13052|652[0n]bp
Chromis atripectoralis|TZAIC534-06|HLC-11602|639[0n]bp
Chromis caerulea|TZAIC226-05|HLC-10926|652[1n]bp
Chromis caerulea|TZAIC224-05|HLC-10924|652[0n]bp
Chromis caerulea|TZAIC222-05|HLC-10922|652[1n]bp
Chromis viridis|TZAIC225-05|HLC-10925|652[0n]bp
Chromis viridis|TZAIC533-06|HLC-11601|652[0n]bp
Chromis viridis|TZAIC530-06|HLC-11598|652[0n]bp
Chromis viridis|TZAIC531-06|HLC-11599|652[0n]bp
Chromis viridis|TZAIC532-06|HLC-11600|652[0n]bp
Microspathodon chrysurus|TZAIB280-06|HLC-12015|629[0n]bp
Microspathodon chrysurus|TZAIB281-06|HLC-12016|617[0n]bp
Microspathodon chrysurus|TZAIB279-06|HLC-12014|652[0n]bp
Microspathodon chrysurus|TZAIB278-06|HLC-12013|652[0n]bp
Chrysiptera galba|TZAIB068-06|HLC-12179|652[0n]bp
Chrysiptera galba|TZAIB072-06|HLC-12183|652[0n]bp
Chrysiptera galba|TZAIB069-06|HLC-12180|652[2n]bp
Chrysiptera galba|TZAIB070-06|HLC-12181|623[0n]bp
Chrysiptera galba|TZAIB071-06|HLC-12182|652[1n]bp
Chrysiptera rex|TZAIC138-05|HLC-10838|652[1n]bp
Chrysiptera rex|TZAIC548-06|HLC-11616|652[0n]bp
Chrysiptera rex|TZAIC137-05|HLC-10837|652[0n]bp
Chrysiptera rex|TZAIC549-06|HLC-11617|652[0n]bp
Chrysiptera cyanea|TZAIC055-05|HLC-10755|652[0n]bp
Chrysiptera cyanea|TZAIC054-05|HLC-10754|652[0n]bp
Chrysiptera cyanea|TZAIC056-05|HLC-10756|652[0n]bp
Chrysiptera cyanea|TZAIB209-06|HLC-11944|652[0n]bp
Chrysiptera cyanea|TZAIC569-06|HLC-11637|652[0n]bp
Chrysiptera cyanea|TZAIC566-06|HLC-11634|652[0n]bp
Chrysiptera cyanea|TZAIC567-06|HLC-11635|652[0n]bp
Chrysiptera cyanea|TZAIB208-06|HLC-11943|652[1n]bp
Chrysiptera cyanea|TZAIC053-05|HLC-10753|652[0n]bp
Chrysiptera cyanea|TZAIC052-05|HLC-10752|652[0n]bp
Chrysiptera cyanea|TZAIB210-06|HLC-11945|616[1n]bp
Chrysiptera cyanea|TZAIB206-06|HLC-11941|652[1n]bp
Chrysiptera cyanea|TZAIB207-06|HLC-11942|652[2n]bp
Chrysiptera cyanea|TZAIC565-06|HLC-11633|652[0n]bp
Chrysiptera cyanea|TZAIC568-06|HLC-11636|652[0n]bp
Chrysiptera taupou|TZAIC204-05|HLC-10904|652[0n]bp
Chrysiptera taupou|TZAIC439-05|HLC-11147|651[0n]bp
Chrysiptera taupou|TZAIC438-05|HLC-11146|651[0n]bp
Chrysiptera taupou|TZAIC440-05|HLC-11148|651[2n]bp
Chrysiptera taupou|TZAIC441-05|HLC-11149|651[1n]bp
Chrysiptera parasema|TZAIC051-05|HLC-10751|652[0n]bp
Chrysiptera parasema|TZAIC048-05|HLC-10748|650[0n]bp
Chrysiptera parasema|TZAIC047-05|HLC-10747|633[0n]bp
Chrysiptera parasema|TZAIC050-05|HLC-10750|575[3n]bp
Chrysiptera parasema|TZAIC049-05|HLC-10749|652[0n]bp
Chrysiptera starcki|TZAIB630-06|HLC-13253|631[0n]bp
Chrysiptera starcki|TZAIB631-06|HLC-13254|652[0n]bp
Chrysiptera starcki|TZAIB629-06|HLC-13252|652[0n]bp
Chrysiptera starcki|TZAIB632-06|HLC-13255|652[0n]bp
Chrysiptera starcki|TZAIC576-06|HLC-11644|652[0n]bp
Chrysiptera starcki|TZAIB043-06|HLC-12154|652[0n]bp
Chrysiptera starcki|TZAIC575-06|HLC-11643|651[0n]bp

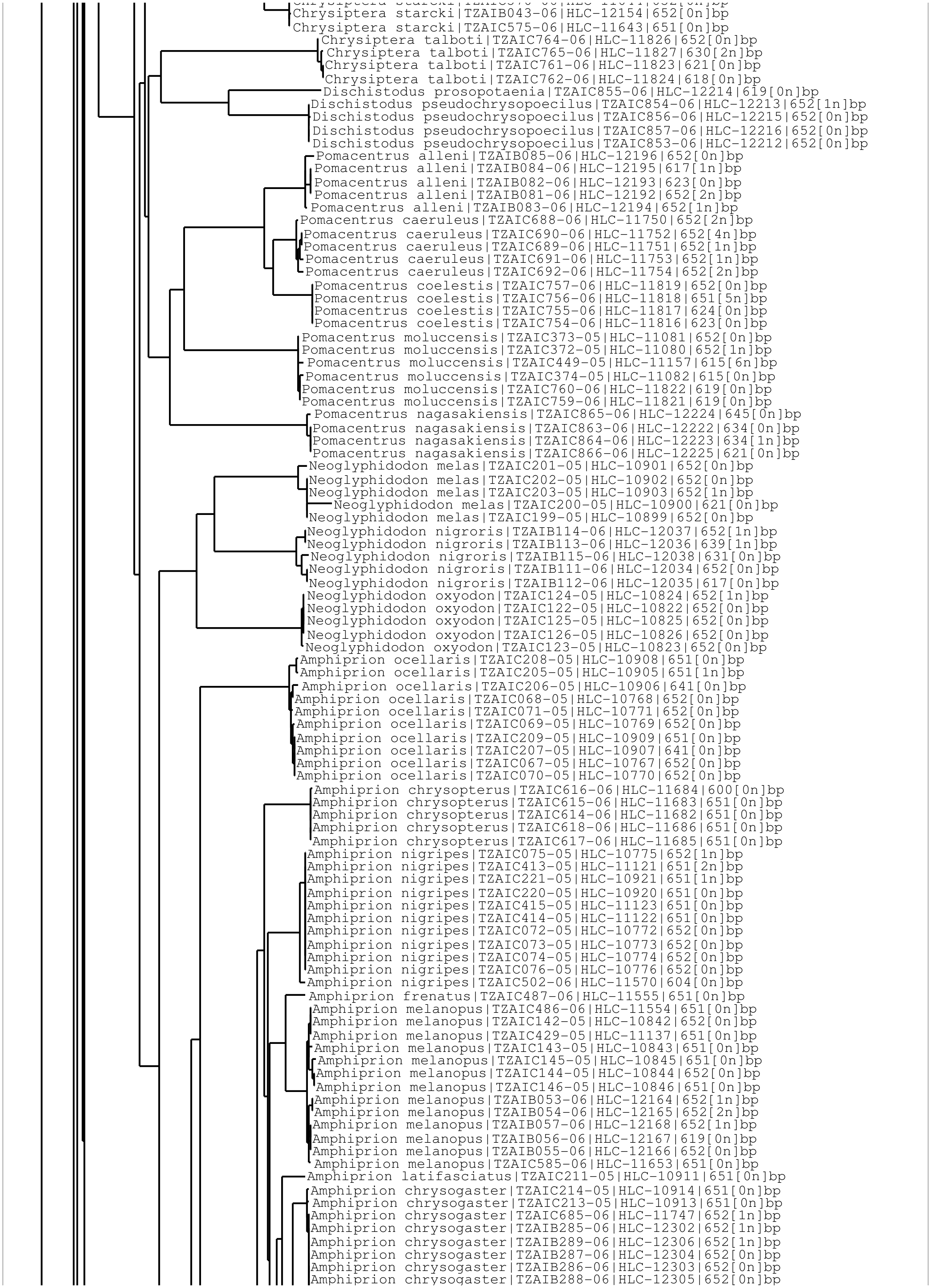
Chrysiptera starcki|TZAIB043-06|HLC-12154|652[0n]bp
Chrysiptera starcki|TZAIC575-06|HLC-11643|651[0n]bp
Chrysiptera talboti|TZAIC764-06|HLC-11826|652[0n]bp
Chrysiptera talboti|TZAIC765-06|HLC-11827|630[2n]bp
Chrysiptera talboti|TZAIC761-06|HLC-11823|621[0n]bp
Chrysiptera talboti|TZAIC762-06|HLC-11824|618[0n]bp
Dischistodus prosopotaenia|TZAIC855-06|HLC-12214|619[0n]bp
Dischistodus pseudochrysopoecilus|TZAIC854-06|HLC-12213|652[1n]bp
Dischistodus pseudochrysopoecilus|TZAIC856-06|HLC-12215|652[0n]bp
Dischistodus pseudochrysopoecilus|TZAIC857-06|HLC-12216|652[0n]bp
Dischistodus pseudochrysopoecilus|TZAIC853-06|HLC-12212|652[0n]bp
Pomacentrus alleni|TZAIB085-06|HLC-12196|652[0n]bp
Pomacentrus alleni|TZAIB084-06|HLC-12195|617[1n]bp
Pomacentrus alleni|TZAIB082-06|HLC-12193|623[0n]bp
Pomacentrus alleni|TZAIB081-06|HLC-12192|652[2n]bp
Pomacentrus alleni|TZAIB083-06|HLC-12194|652[1n]bp
Pomacentrus caeruleus|TZAIC688-06|HLC-11750|652[2n]bp
Pomacentrus caeruleus|TZAIC690-06|HLC-11752|652[4n]bp
Pomacentrus caeruleus|TZAIC689-06|HLC-11751|652[1n]bp
Pomacentrus caeruleus|TZAIC691-06|HLC-11753|652[1n]bp
Pomacentrus caeruleus|TZAIC692-06|HLC-11754|652[2n]bp
Pomacentrus coelestis|TZAIC757-06|HLC-11819|652[0n]bp
Pomacentrus coelestis|TZAIC756-06|HLC-11818|651[5n]bp
Pomacentrus coelestis|TZAIC755-06|HLC-11817|624[0n]bp
Pomacentrus coelestis|TZAIC754-06|HLC-11816|623[0n]bp
Pomacentrus moluccensis|TZAIC373-05|HLC-11081|652[0n]bp
Pomacentrus moluccensis|TZAIC372-05|HLC-11080|652[1n]bp
Pomacentrus moluccensis|TZAIC449-05|HLC-11157|615[6n]bp
Pomacentrus moluccensis|TZAIC374-05|HLC-11082|615[0n]bp
Pomacentrus moluccensis|TZAIC760-06|HLC-11822|619[0n]bp
Pomacentrus moluccensis|TZAIC759-06|HLC-11821|619[0n]bp
Pomacentrus nagasakiensis|TZAIC865-06|HLC-12224|645[0n]bp
Pomacentrus nagasakiensis|TZAIC863-06|HLC-12222|634[0n]bp
Pomacentrus nagasakiensis|TZAIC864-06|HLC-12223|634[1n]bp
Pomacentrus nagasakiensis|TZAIC866-06|HLC-12225|621[0n]bp
Neoglyphidodon melas|TZAIC201-05|HLC-10901|652[0n]bp
Neoglyphidodon melas|TZAIC202-05|HLC-10902|652[0n]bp
Neoglyphidodon melas|TZAIC203-05|HLC-10903|652[1n]bp
Neoglyphidodon melas|TZAIC200-05|HLC-10900|621[0n]bp
Neoglyphidodon melas|TZAIC199-05|HLC-10899|652[0n]bp
Neoglyphidodon nigroris|TZAIB114-06|HLC-12037|652[1n]bp
Neoglyphidodon nigroris|TZAIB113-06|HLC-12036|639[1n]bp
Neoglyphidodon nigroris|TZAIB115-06|HLC-12038|631[0n]bp
Neoglyphidodon nigroris|TZAIB111-06|HLC-12034|652[0n]bp
Neoglyphidodon nigroris|TZAIB112-06|HLC-12035|617[0n]bp
Neoglyphidodon oxyodon|TZAIC124-05|HLC-10824|652[1n]bp
Neoglyphidodon oxyodon|TZAIC122-05|HLC-10822|652[0n]bp
Neoglyphidodon oxyodon|TZAIC125-05|HLC-10825|652[0n]bp
Neoglyphidodon oxyodon|TZAIC126-05|HLC-10826|652[0n]bp
Neoglyphidodon oxyodon|TZAIC123-05|HLC-10823|652[0n]bp
Amphiprion ocellaris|TZAIC208-05|HLC-10908|651[0n]bp
Amphiprion ocellaris|TZAIC205-05|HLC-10905|651[1n]bp
Amphiprion ocellaris|TZAIC206-05|HLC-10906|641[0n]bp
Amphiprion ocellaris|TZAIC068-05|HLC-10768|652[0n]bp
Amphiprion ocellaris|TZAIC071-05|HLC-10771|652[0n]bp
Amphiprion ocellaris|TZAIC069-05|HLC-10769|652[0n]bp
Amphiprion ocellaris|TZAIC209-05|HLC-10909|651[0n]bp
Amphiprion ocellaris|TZAIC207-05|HLC-10907|641[0n]bp
Amphiprion ocellaris|TZAIC067-05|HLC-10767|652[0n]bp
Amphiprion ocellaris|TZAIC070-05|HLC-10770|652[0n]bp
Amphiprion chrysopterus|TZAIC616-06|HLC-11684|600[0n]bp
Amphiprion chrysopterus|TZAIC615-06|HLC-11683|651[0n]bp
Amphiprion chrysopterus|TZAIC614-06|HLC-11682|651[0n]bp
Amphiprion chrysopterus|TZAIC618-06|HLC-11686|651[0n]bp
Amphiprion chrysopterus|TZAIC617-06|HLC-11685|651[0n]bp
Amphiprion nigripes|TZAIC075-05|HLC-10775|652[1n]bp
Amphiprion nigripes|TZAIC413-05|HLC-11121|651[2n]bp
Amphiprion nigripes|TZAIC221-05|HLC-10921|651[1n]bp
Amphiprion nigripes|TZAIC220-05|HLC-10920|651[0n]bp
Amphiprion nigripes|TZAIC415-05|HLC-11123|651[0n]bp
Amphiprion nigripes|TZAIC414-05|HLC-11122|651[0n]bp
Amphiprion nigripes|TZAIC072-05|HLC-10772|652[0n]bp
Amphiprion nigripes|TZAIC073-05|HLC-10773|652[0n]bp
Amphiprion nigripes|TZAIC074-05|HLC-10774|652[0n]bp
Amphiprion nigripes|TZAIC076-05|HLC-10776|652[0n]bp
Amphiprion nigripes|TZAIC502-06|HLC-11570|604[0n]bp
Amphiprion frenatus|TZAIC487-06|HLC-11555|651[0n]bp
Amphiprion melanopus|TZAIC486-06|HLC-11554|651[0n]bp
Amphiprion melanopus|TZAIC142-05|HLC-10842|652[0n]bp
Amphiprion melanopus|TZAIC429-05|HLC-11137|651[0n]bp
Amphiprion melanopus|TZAIC143-05|HLC-10843|651[0n]bp
Amphiprion melanopus|TZAIC145-05|HLC-10845|651[0n]bp
Amphiprion melanopus|TZAIC144-05|HLC-10844|652[0n]bp
Amphiprion melanopus|TZAIC146-05|HLC-10846|651[0n]bp
Amphiprion melanopus|TZAIB053-06|HLC-12164|652[1n]bp
Amphiprion melanopus|TZAIB054-06|HLC-12165|652[2n]bp
Amphiprion melanopus|TZAIB057-06|HLC-12168|652[1n]bp
Amphiprion melanopus|TZAIB056-06|HLC-12167|619[0n]bp
Amphiprion melanopus|TZAIB055-06|HLC-12166|652[0n]bp
Amphiprion melanopus|TZAIC585-06|HLC-11653|651[0n]bp
Amphiprion latifasciatus|TZAIC211-05|HLC-10911|651[0n]bp
Amphiprion chrysogaster|TZAIC214-05|HLC-10914|651[0n]bp
Amphiprion chrysogaster|TZAIC213-05|HLC-10913|651[0n]bp
Amphiprion chrysogaster|TZAIC685-06|HLC-11747|652[1n]bp
Amphiprion chrysogaster|TZAIB285-06|HLC-12302|652[1n]bp
Amphiprion chrysogaster|TZAIB289-06|HLC-12306|652[1n]bp
Amphiprion chrysogaster|TZAIB287-06|HLC-12304|652[0n]bp
Amphiprion chrysogaster|TZAIB286-06|HLC-12303|652[0n]bp
Amphiprion chrysogaster|TZAIB288-06|HLC-12305|652[0n]bp

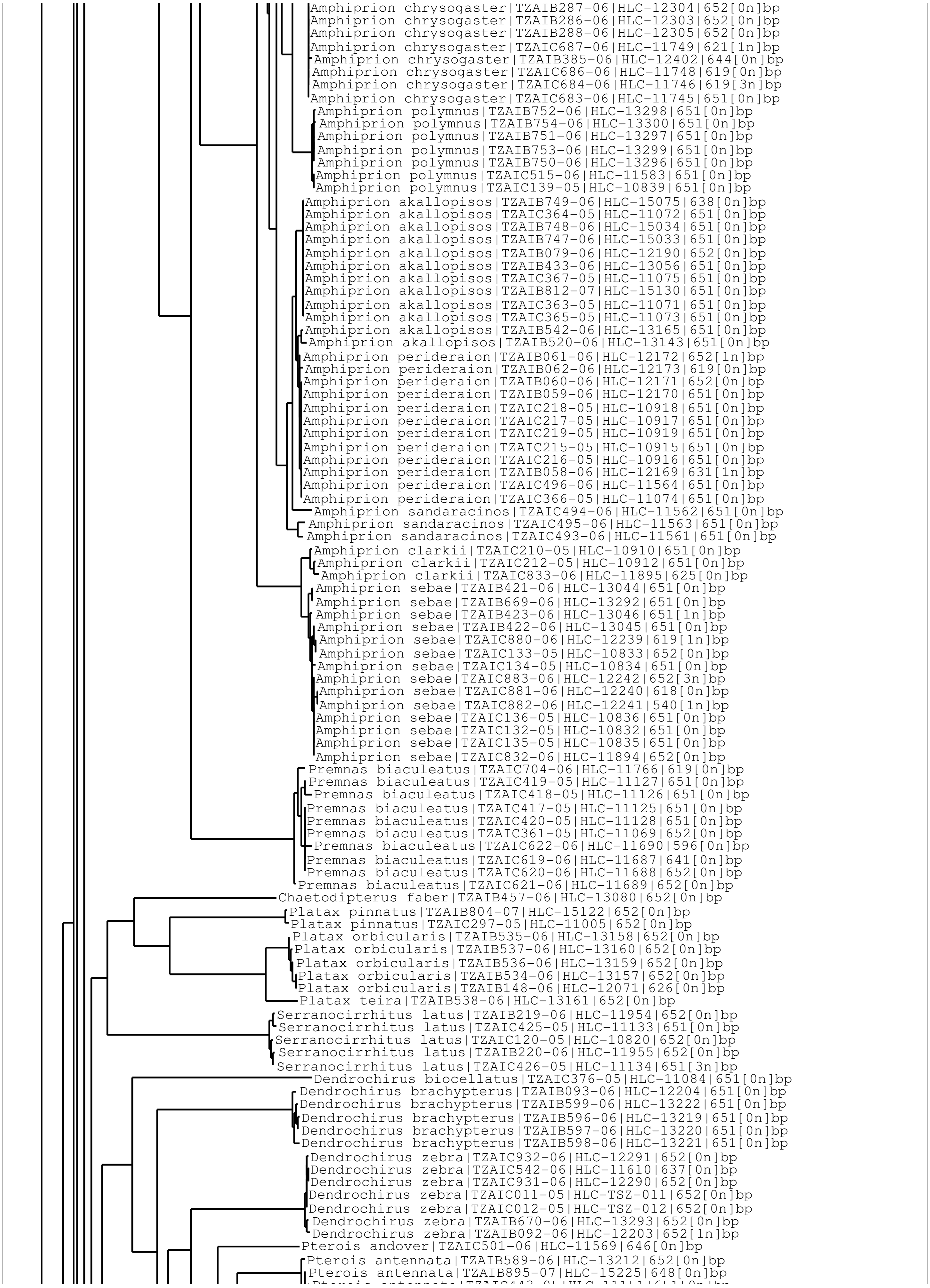
Amphiprion chrysogaster|TZAIB287-06|HLC-12304|652[0n]bp
Amphiprion chrysogaster|TZAIB286-06|HLC-12303|652[0n]bp
Amphiprion chrysogaster|TZAIB288-06|HLC-12305|652[0n]bp
Amphiprion chrysogaster|TZAIC687-06|HLC-11749|621[1n]bp
Amphiprion chrysogaster|TZAIB385-06|HLC-12402|644[0n]bp
Amphiprion chrysogaster|TZAIC686-06|HLC-11748|619[0n]bp
Amphiprion chrysogaster|TZAIC684-06|HLC-11746|619[3n]bp
Amphiprion chrysogaster|TZAIC683-06|HLC-11745|651[0n]bp
Amphiprion polymnus|TZAIB752-06|HLC-13298|651[0n]bp
Amphiprion polymnus|TZAIB754-06|HLC-13300|651[0n]bp
Amphiprion polymnus|TZAIB751-06|HLC-13297|651[0n]bp
Amphiprion polymnus|TZAIB753-06|HLC-13299|651[0n]bp
Amphiprion polymnus|TZAIB750-06|HLC-13296|651[0n]bp
Amphiprion polymnus|TZAIC515-06|HLC-11583|651[0n]bp
Amphiprion polymnus|TZAIC139-05|HLC-10839|651[0n]bp
Amphiprion akallopisos|TZAIB749-06|HLC-15075|638[0n]bp
Amphiprion akallopisos|TZAIC364-05|HLC-11072|651[0n]bp
Amphiprion akallopisos|TZAIB748-06|HLC-15034|651[0n]bp
Amphiprion akallopisos|TZAIB747-06|HLC-15033|651[0n]bp
Amphiprion akallopisos|TZAIB079-06|HLC-12190|652[0n]bp
Amphiprion akallopisos|TZAIB433-06|HLC-13056|651[0n]bp
Amphiprion akallopisos|TZAIC367-05|HLC-11075|651[0n]bp
Amphiprion akallopisos|TZAIB812-07|HLC-15130|651[0n]bp
Amphiprion akallopisos|TZAIC363-05|HLC-11071|651[0n]bp
Amphiprion akallopisos|TZAIC365-05|HLC-11073|651[0n]bp
Amphiprion akallopisos|TZAIB542-06|HLC-13165|651[0n]bp
Amphiprion akallopisos|TZAIB520-06|HLC-13143|651[0n]bp
Amphiprion perideraion|TZAIB061-06|HLC-12172|652[1n]bp
Amphiprion perideraion|TZAIB062-06|HLC-12173|619[0n]bp
Amphiprion perideraion|TZAIB060-06|HLC-12171|652[0n]bp
Amphiprion perideraion|TZAIB059-06|HLC-12170|651[0n]bp
Amphiprion perideraion|TZAIC218-05|HLC-10918|651[0n]bp
Amphiprion perideraion|TZAIC217-05|HLC-10917|651[0n]bp
Amphiprion perideraion|TZAIC219-05|HLC-10919|651[0n]bp
Amphiprion perideraion|TZAIC215-05|HLC-10915|651[0n]bp
Amphiprion perideraion|TZAIC216-05|HLC-10916|651[0n]bp
Amphiprion perideraion|TZAIB058-06|HLC-12169|631[1n]bp
Amphiprion perideraion|TZAIC496-06|HLC-11564|651[0n]bp
Amphiprion perideraion|TZAIC366-05|HLC-11074|651[0n]bp
Amphiprion sandaracinos|TZAIC494-06|HLC-11562|651[0n]bp
Amphiprion sandaracinos|TZAIC495-06|HLC-11563|651[0n]bp
Amphiprion sandaracinos|TZAIC493-06|HLC-11561|651[0n]bp
Amphiprion clarkii|TZAIC210-05|HLC-10910|651[0n]bp
Amphiprion clarkii|TZAIC212-05|HLC-10912|651[0n]bp
Amphiprion clarkii|TZAIC833-06|HLC-11895|625[0n]bp
Amphiprion sebae|TZAIB421-06|HLC-13044|651[0n]bp
Amphiprion sebae|TZAIB669-06|HLC-13292|651[0n]bp
Amphiprion sebae|TZAIB423-06|HLC-13046|651[1n]bp
Amphiprion sebae|TZAIB422-06|HLC-13045|651[0n]bp
Amphiprion sebae|TZAIC880-06|HLC-12239|619[1n]bp
Amphiprion sebae|TZAIC133-05|HLC-10833|652[0n]bp
Amphiprion sebae|TZAIC134-05|HLC-10834|651[0n]bp
Amphiprion sebae|TZAIC883-06|HLC-12242|652[3n]bp
Amphiprion sebae|TZAIC881-06|HLC-12240|618[0n]bp
Amphiprion sebae|TZAIC882-06|HLC-12241|540[1n]bp
Amphiprion sebae|TZAIC136-05|HLC-10836|651[0n]bp
Amphiprion sebae|TZAIC132-05|HLC-10832|651[0n]bp
Amphiprion sebae|TZAIC135-05|HLC-10835|651[0n]bp
Amphiprion sebae|TZAIC832-06|HLC-11894|652[0n]bp
Premnas biaculeatus|TZAIC704-06|HLC-11766|619[0n]bp
Premnas biaculeatus|TZAIC419-05|HLC-11127|651[0n]bp
Premnas biaculeatus|TZAIC418-05|HLC-11126|651[0n]bp
Premnas biaculeatus|TZAIC417-05|HLC-11125|651[0n]bp
Premnas biaculeatus|TZAIC420-05|HLC-11128|651[0n]bp
Premnas biaculeatus|TZAIC361-05|HLC-11069|652[0n]bp
Premnas biaculeatus|TZAIC622-06|HLC-11690|596[0n]bp
Premnas biaculeatus|TZAIC619-06|HLC-11687|641[0n]bp
Premnas biaculeatus|TZAIC620-06|HLC-11688|652[0n]bp
Premnas biaculeatus|TZAIC621-06|HLC-11689|652[0n]bp
Chaetodipterus faber|TZAIB457-06|HLC-13080|652[0n]bp
Platax pinnatus|TZAIB804-07|HLC-15122|652[0n]bp
Platax pinnatus|TZAIC297-05|HLC-11005|652[0n]bp
Platax orbicularis|TZAIB535-06|HLC-13158|652[0n]bp
Platax orbicularis|TZAIB537-06|HLC-13160|652[0n]bp
Platax orbicularis|TZAIB536-06|HLC-13159|652[0n]bp
Platax orbicularis|TZAIB534-06|HLC-13157|652[0n]bp
Platax orbicularis|TZAIB148-06|HLC-12071|626[0n]bp
Platax teira|TZAIB538-06|HLC-13161|652[0n]bp
Serranocirrhitus latus|TZAIB219-06|HLC-11954|652[0n]bp
Serranocirrhitus latus|TZAIC425-05|HLC-11133|651[0n]bp
Serranocirrhitus latus|TZAIC120-05|HLC-10820|652[0n]bp
Serranocirrhitus latus|TZAIB220-06|HLC-11955|652[0n]bp
Serranocirrhitus latus|TZAIC426-05|HLC-11134|651[3n]bp
Dendrochirus biocellatus|TZAIC376-05|HLC-11084|651[0n]bp
Dendrochirus brachypterus|TZAIB093-06|HLC-12204|651[0n]bp
Dendrochirus brachypterus|TZAIB599-06|HLC-13222|651[0n]bp
Dendrochirus brachypterus|TZAIB596-06|HLC-13219|651[0n]bp
Dendrochirus brachypterus|TZAIB597-06|HLC-13220|651[0n]bp
Dendrochirus brachypterus|TZAIB598-06|HLC-13221|651[0n]bp
Dendrochirus zebra|TZAIC932-06|HLC-12291|652[0n]bp
Dendrochirus zebra|TZAIC542-06|HLC-11610|637[0n]bp
Dendrochirus zebra|TZAIC931-06|HLC-12290|652[0n]bp
Dendrochirus zebra|TZAIC011-05|HLC-TSZ-011|652[0n]bp
Dendrochirus zebra|TZAIC012-05|HLC-TSZ-012|652[0n]bp
Dendrochirus zebra|TZAIB670-06|HLC-13293|652[0n]bp
Dendrochirus zebra|TZAIB092-06|HLC-12203|652[1n]bp
Pterois andover|TZAIC501-06|HLC-11569|646[0n]bp
Pterois antennata|TZAIB589-06|HLC-13212|652[0n]bp
Pterois antennata|TZAIB895-07|HLC-15225|648[0n]bp

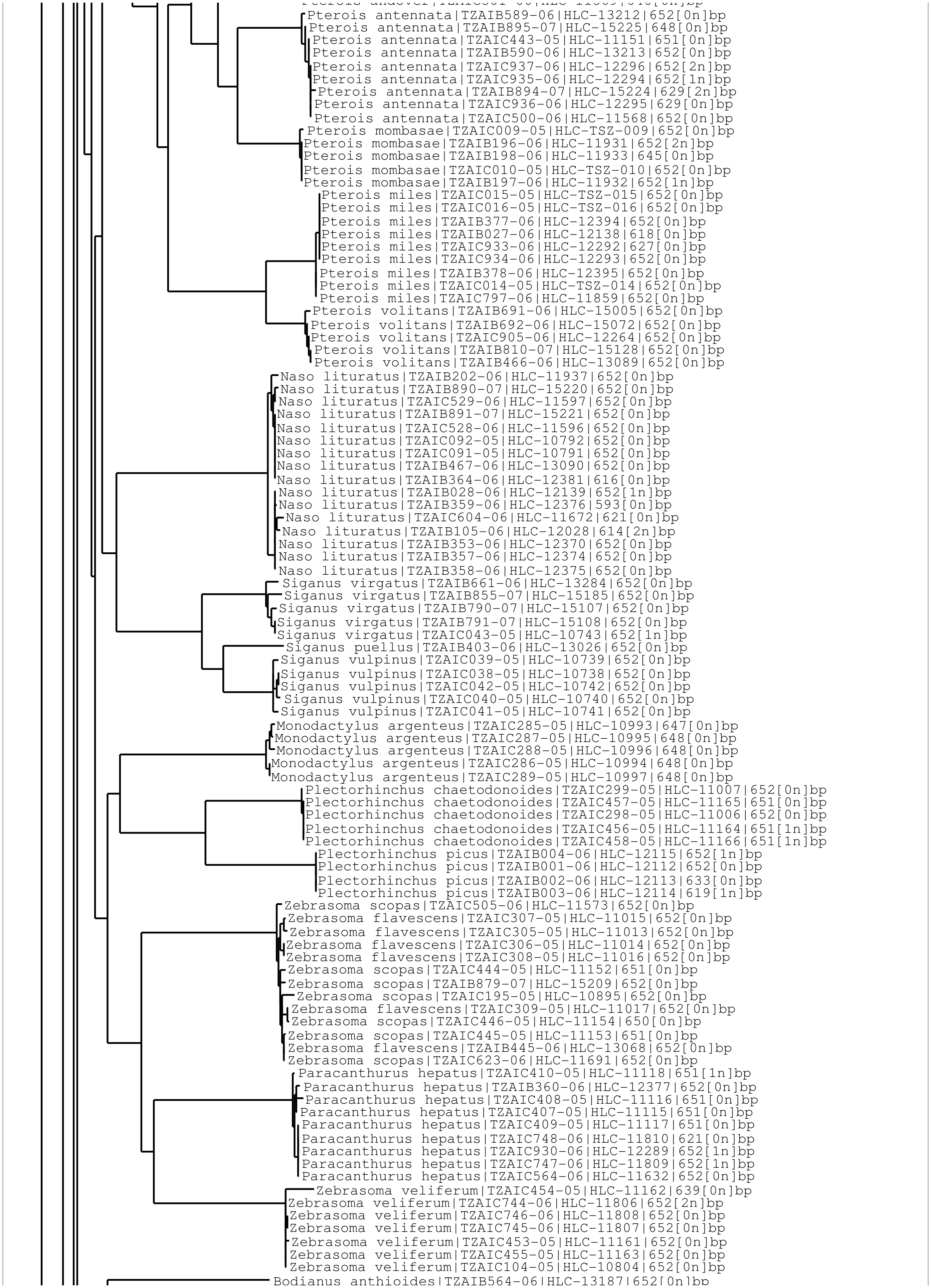
Pterois antennata|TZAIB589-06|HLC-13212|652[0n]bp
Pterois antennata|TZAIB895-07|HLC-15225|648[0n]bp
Pterois antennata|TZAIC443-05|HLC-11151|651[0n]bp
Pterois antennata|TZAIB590-06|HLC-13213|652[0n]bp
Pterois antennata|TZAIC937-06|HLC-12296|652[2n]bp
Pterois antennata|TZAIC935-06|HLC-12294|652[1n]bp
Pterois antennata|TZAIB894-07|HLC-15224|629[2n]bp
Pterois antennata|TZAIC936-06|HLC-12295|629[0n]bp
Pterois antennata|TZAIC500-06|HLC-11568|652[0n]bp
Pterois mombasae|TZAIC009-05|HLC-TSZ-009|652[0n]bp
Pterois mombasae|TZAIB196-06|HLC-11931|652[2n]bp
Pterois mombasae|TZAIB198-06|HLC-11933|645[0n]bp
Pterois mombasae|TZAIC010-05|HLC-TSZ-010|652[0n]bp
Pterois mombasae|TZAIB197-06|HLC-11932|652[1n]bp
Pterois miles|TZAIC015-05|HLC-TSZ-015|652[0n]bp
Pterois miles|TZAIC016-05|HLC-TSZ-016|652[0n]bp
Pterois miles|TZAIB377-06|HLC-12394|652[0n]bp
Pterois miles|TZAIB027-06|HLC-12138|618[0n]bp
Pterois miles|TZAIC933-06|HLC-12292|627[0n]bp
Pterois miles|TZAIC934-06|HLC-12293|652[0n]bp
Pterois miles|TZAIB378-06|HLC-12395|652[0n]bp
Pterois miles|TZAIC014-05|HLC-TSZ-014|652[0n]bp
Pterois miles|TZAIC797-06|HLC-11859|652[0n]bp
Pterois volitans|TZAIB691-06|HLC-15005|652[0n]bp
Pterois volitans|TZAIB692-06|HLC-15072|652[0n]bp
Pterois volitans|TZAIC905-06|HLC-12264|652[0n]bp
Pterois volitans|TZAIB810-07|HLC-15128|652[0n]bp
Pterois volitans|TZAIB466-06|HLC-13089|652[0n]bp
Naso lituratus|TZAIB202-06|HLC-11937|652[0n]bp
Naso lituratus|TZAIB890-07|HLC-15220|652[0n]bp
Naso lituratus|TZAIC529-06|HLC-11597|652[0n]bp
Naso lituratus|TZAIB891-07|HLC-15221|652[0n]bp
Naso lituratus|TZAIC528-06|HLC-11596|652[0n]bp
Naso lituratus|TZAIC092-05|HLC-10792|652[0n]bp
Naso lituratus|TZAIC091-05|HLC-10791|652[0n]bp
Naso lituratus|TZAIB467-06|HLC-13090|652[0n]bp
Naso lituratus|TZAIB364-06|HLC-12381|616[0n]bp
Naso lituratus|TZAIB028-06|HLC-12139|652[1n]bp
Naso lituratus|TZAIB359-06|HLC-12376|593[0n]bp
Naso lituratus|TZAIC604-06|HLC-11672|621[0n]bp
Naso lituratus|TZAIB105-06|HLC-12028|614[2n]bp
Naso lituratus|TZAIB353-06|HLC-12370|652[0n]bp
Naso lituratus|TZAIB357-06|HLC-12374|652[0n]bp
Naso lituratus|TZAIB358-06|HLC-12375|652[0n]bp
Siganus virgatus|TZAIB661-06|HLC-13284|652[0n]bp
Siganus virgatus|TZAIB855-07|HLC-15185|652[0n]bp
Siganus virgatus|TZAIB790-07|HLC-15107|652[0n]bp
Siganus virgatus|TZAIB791-07|HLC-15108|652[0n]bp
Siganus virgatus|TZAIC043-05|HLC-10743|652[1n]bp
Siganus puellus|TZAIB403-06|HLC-13026|652[0n]bp
Siganus vulpinus|TZAIC039-05|HLC-10739|652[0n]bp
Siganus vulpinus|TZAIC038-05|HLC-10738|652[0n]bp
Siganus vulpinus|TZAIC042-05|HLC-10742|652[0n]bp
Siganus vulpinus|TZAIC040-05|HLC-10740|652[0n]bp
Siganus vulpinus|TZAIC041-05|HLC-10741|652[0n]bp
Monodactylus argenteus|TZAIC285-05|HLC-10993|647[0n]bp
Monodactylus argenteus|TZAIC287-05|HLC-10995|648[0n]bp
Monodactylus argenteus|TZAIC288-05|HLC-10996|648[0n]bp
Monodactylus argenteus|TZAIC286-05|HLC-10994|648[0n]bp
Monodactylus argenteus|TZAIC289-05|HLC-10997|648[0n]bp
Plectorhinchus chaetodonoides|TZAIC299-05|HLC-11007|652[0n]bp
Plectorhinchus chaetodonoides|TZAIC457-05|HLC-11165|651[0n]bp
Plectorhinchus chaetodonoides|TZAIC298-05|HLC-11006|652[0n]bp
Plectorhinchus chaetodonoides|TZAIC456-05|HLC-11164|651[1n]bp
Plectorhinchus chaetodonoides|TZAIC458-05|HLC-11166|651[1n]bp
Plectorhinchus picus|TZAIB004-06|HLC-12115|652[1n]bp
Plectorhinchus picus|TZAIB001-06|HLC-12112|652[0n]bp
Plectorhinchus picus|TZAIB002-06|HLC-12113|633[0n]bp
Plectorhinchus picus|TZAIB003-06|HLC-12114|619[1n]bp
Zebrasoma scopas|TZAIC505-06|HLC-11573|652[0n]bp
Zebrasoma flavescens|TZAIC307-05|HLC-11015|652[0n]bp
Zebrasoma flavescens|TZAIC305-05|HLC-11013|652[0n]bp
Zebrasoma flavescens|TZAIC306-05|HLC-11014|652[0n]bp
Zebrasoma flavescens|TZAIC308-05|HLC-11016|652[0n]bp
Zebrasoma scopas|TZAIC444-05|HLC-11152|651[0n]bp
Zebrasoma scopas|TZAIB879-07|HLC-15209|652[0n]bp
Zebrasoma scopas|TZAIC195-05|HLC-10895|652[0n]bp
Zebrasoma flavescens|TZAIC309-05|HLC-11017|652[0n]bp
Zebrasoma scopas|TZAIC446-05|HLC-11154|650[0n]bp
Zebrasoma scopas|TZAIC445-05|HLC-11153|651[0n]bp
Zebrasoma flavescens|TZAIB445-06|HLC-13068|652[0n]bp
Zebrasoma scopas|TZAIC623-06|HLC-11691|652[0n]bp
Paracanthurus hepatus|TZAIC410-05|HLC-11118|651[1n]bp
Paracanthurus hepatus|TZAIB360-06|HLC-12377|652[0n]bp
Paracanthurus hepatus|TZAIC408-05|HLC-11116|651[0n]bp
Paracanthurus hepatus|TZAIC407-05|HLC-11115|651[0n]bp
Paracanthurus hepatus|TZAIC409-05|HLC-11117|651[0n]bp
Paracanthurus hepatus|TZAIC748-06|HLC-11810|621[0n]bp
Paracanthurus hepatus|TZAIC930-06|HLC-12289|652[1n]bp
Paracanthurus hepatus|TZAIC747-06|HLC-11809|652[1n]bp
Paracanthurus hepatus|TZAIC564-06|HLC-11632|652[0n]bp
Zebrasoma veliferum|TZAIC454-05|HLC-11162|639[0n]bp
Zebrasoma veliferum|TZAIC744-06|HLC-11806|652[2n]bp
Zebrasoma veliferum|TZAIC746-06|HLC-11808|652[0n]bp
Zebrasoma veliferum|TZAIC745-06|HLC-11807|652[0n]bp
Zebrasoma veliferum|TZAIC453-05|HLC-11161|652[0n]bp
Zebrasoma veliferum|TZAIC455-05|HLC-11163|652[0n]bp
Zebrasoma veliferum|TZAIC104-05|HLC-10804|652[0n]bp
Bodianus anthioides|TZAIB564-06|HLC-13187|652[0n]bp

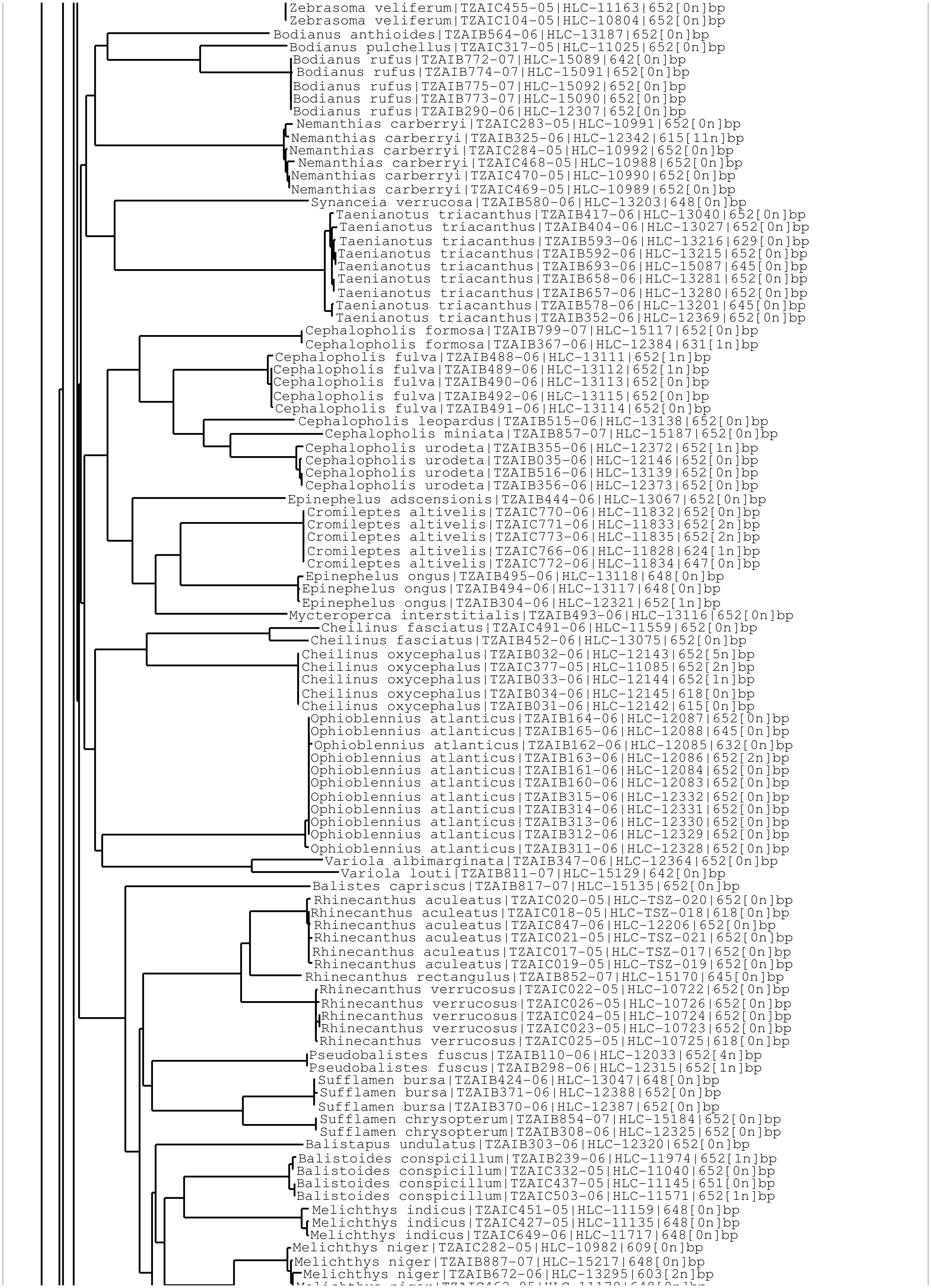

Zebrasoma veliferum|TZAIC455-05|HLC-11163|652[0n]bp
Zebrasoma veliferum|TZAIC104-05|HLC-10804|652[0n]bp
Bodianus anthioides|TZAIB564-06|HLC-13187|652[0n]bp
Bodianus pulchellus|TZAIC317-05|HLC-11025|652[0n]bp
Bodianus rufus|TZAIB772-07|HLC-15089|642[0n]bp
Bodianus rufus|TZAIB774-07|HLC-15091|652[0n]bp
Bodianus rufus|TZAIB775-07|HLC-15092|652[0n]bp
Bodianus rufus|TZAIB773-07|HLC-15090|652[0n]bp
Bodianus rufus|TZAIB290-06|HLC-12307|652[0n]bp
Nemanthias carberryi|TZAIC283-05|HLC-10991|652[0n]bp
Nemanthias carberryi|TZAIB325-06|HLC-12342|615[11n]bp
Nemanthias carberryi|TZAIC284-05|HLC-10992|652[0n]bp
Nemanthias carberryi|TZAIC468-05|HLC-10988|652[0n]bp
Nemanthias carberryi|TZAIC470-05|HLC-10990|652[0n]bp
Nemanthias carberryi|TZAIC469-05|HLC-10989|652[0n]bp
Synanceia verrucosa|TZAIB580-06|HLC-13203|648[0n]bp
Taenianotus triacanthus|TZAIB417-06|HLC-13040|652[0n]bp
Taenianotus triacanthus|TZAIB404-06|HLC-13027|652[0n]bp
Taenianotus triacanthus|TZAIB593-06|HLC-13216|629[0n]bp
Taenianotus triacanthus|TZAIB592-06|HLC-13215|652[0n]bp
Taenianotus triacanthus|TZAIB693-06|HLC-15087|645[0n]bp
Taenianotus triacanthus|TZAIB658-06|HLC-13281|652[0n]bp
Taenianotus triacanthus|TZAIB657-06|HLC-13280|652[0n]bp
Taenianotus triacanthus|TZAIB578-06|HLC-13201|645[0n]bp
Taenianotus triacanthus|TZAIB352-06|HLC-12369|652[0n]bp
Cephalopholis formosa|TZAIB799-07|HLC-15117|652[0n]bp
Cephalopholis formosa|TZAIB367-06|HLC-12384|631[1n]bp
Cephalopholis fulva|TZAIB488-06|HLC-13111|652[1n]bp
Cephalopholis fulva|TZAIB489-06|HLC-13112|652[1n]bp
Cephalopholis fulva|TZAIB490-06|HLC-13113|652[0n]bp
Cephalopholis fulva|TZAIB492-06|HLC-13115|652[0n]bp
Cephalopholis fulva|TZAIB491-06|HLC-13114|652[0n]bp
Cephalopholis leopardus|TZAIB515-06|HLC-13138|652[0n]bp
Cephalopholis miniata|TZAIB857-07|HLC-15187|652[0n]bp
Cephalopholis urodeta|TZAIB355-06|HLC-12372|652[1n]bp
Cephalopholis urodeta|TZAIB035-06|HLC-12146|652[0n]bp
Cephalopholis urodeta|TZAIB516-06|HLC-13139|652[0n]bp
Cephalopholis urodeta|TZAIB356-06|HLC-12373|652[0n]bp
Epinephelus adscensionis|TZAIB444-06|HLC-13067|652[0n]bp
Cromileptes altivelis|TZAIC770-06|HLC-11832|652[0n]bp
Cromileptes altivelis|TZAIC771-06|HLC-11833|652[2n]bp
Cromileptes altivelis|TZAIC773-06|HLC-11835|652[2n]bp
Cromileptes altivelis|TZAIC766-06|HLC-11828|624[1n]bp
Cromileptes altivelis|TZAIC772-06|HLC-11834|647[0n]bp
Epinephelus ongus|TZAIB495-06|HLC-13118|648[0n]bp
Epinephelus ongus|TZAIB494-06|HLC-13117|648[0n]bp
Epinephelus ongus|TZAIB304-06|HLC-12321|652[1n]bp
Mycteroperca interstitialis|TZAIB493-06|HLC-13116|652[0n]bp
Cheilinus fasciatus|TZAIC491-06|HLC-11559|652[0n]bp
Cheilinus fasciatus|TZAIB452-06|HLC-13075|652[0n]bp
Cheilinus oxycephalus|TZAIB032-06|HLC-12143|652[5n]bp
Cheilinus oxycephalus|TZAIC377-05|HLC-11085|652[2n]bp
Cheilinus oxycephalus|TZAIB033-06|HLC-12144|652[1n]bp
Cheilinus oxycephalus|TZAIB034-06|HLC-12145|618[0n]bp
Cheilinus oxycephalus|TZAIB031-06|HLC-12142|615[0n]bp
Ophioblennius atlanticus|TZAIB164-06|HLC-12087|652[0n]bp
Ophioblennius atlanticus|TZAIB165-06|HLC-12088|645[0n]bp
Ophioblennius atlanticus|TZAIB162-06|HLC-12085|632[0n]bp
Ophioblennius atlanticus|TZAIB163-06|HLC-12086|652[2n]bp
Ophioblennius atlanticus|TZAIB161-06|HLC-12084|652[0n]bp
Ophioblennius atlanticus|TZAIB160-06|HLC-12083|652[0n]bp
Ophioblennius atlanticus|TZAIB315-06|HLC-12332|652[0n]bp
Ophioblennius atlanticus|TZAIB314-06|HLC-12331|652[0n]bp
Ophioblennius atlanticus|TZAIB313-06|HLC-12330|652[0n]bp
Ophioblennius atlanticus|TZAIB312-06|HLC-12329|652[0n]bp
Ophioblennius atlanticus|TZAIB311-06|HLC-12328|652[0n]bp
Variola albimarginata|TZAIB347-06|HLC-12364|652[0n]bp
Variola louti|TZAIB811-07|HLC-15129|642[0n]bp
Balistes capriscus|TZAIB817-07|HLC-15135|652[0n]bp
Rhinecanthus aculeatus|TZAIC020-05|HLC-TSZ-020|652[0n]bp
Rhinecanthus aculeatus|TZAIC018-05|HLC-TSZ-018|618[0n]bp
Rhinecanthus aculeatus|TZAIC847-06|HLC-12206|652[0n]bp
Rhinecanthus aculeatus|TZAIC021-05|HLC-TSZ-021|652[0n]bp
Rhinecanthus aculeatus|TZAIC017-05|HLC-TSZ-017|652[0n]bp
Rhinecanthus aculeatus|TZAIC019-05|HLC-TSZ-019|652[0n]bp
Rhinecanthus rectangulus|TZAIB852-07|HLC-15170|645[0n]bp
Rhinecanthus verrucosus|TZAIC022-05|HLC-10722|652[0n]bp
Rhinecanthus verrucosus|TZAIC026-05|HLC-10726|652[0n]bp
Rhinecanthus verrucosus|TZAIC024-05|HLC-10724|652[0n]bp
Rhinecanthus verrucosus|TZAIC023-05|HLC-10723|652[0n]bp
Rhinecanthus verrucosus|TZAIC025-05|HLC-10725|618[0n]bp
Pseudobalistes fuscus|TZAIB110-06|HLC-12033|652[4n]bp
Pseudobalistes fuscus|TZAIB298-06|HLC-12315|652[1n]bp
Sufflamen bursa|TZAIB424-06|HLC-13047|648[0n]bp
Sufflamen bursa|TZAIB371-06|HLC-12388|652[0n]bp
Sufflamen bursa|TZAIB370-06|HLC-12387|652[0n]bp
Sufflamen chrysopterum|TZAIB854-07|HLC-15184|652[0n]bp
Sufflamen chrysopterum|TZAIB308-06|HLC-12325|652[0n]bp
Balistapus undulatus|TZAIB303-06|HLC-12320|652[0n]bp
Balistoides conspicillum|TZAIB239-06|HLC-11974|652[1n]bp
Balistoides conspicillum|TZAIC332-05|HLC-11040|652[0n]bp
Balistoides conspicillum|TZAIC437-05|HLC-11145|651[0n]bp
Balistoides conspicillum|TZAIC503-06|HLC-11571|652[1n]bp
Melichthys indicus|TZAIC451-05|HLC-11159|648[0n]bp
Melichthys indicus|TZAIC427-05|HLC-11135|648[0n]bp
Melichthys indicus|TZAIC649-06|HLC-11717|648[0n]bp
Melichthys niger|TZAIC282-05|HLC-10982|609[0n]bp
Melichthys niger|TZAIB887-07|HLC-15217|648[0n]bp
Melichthys niger|TZAIB672-06|HLC-13295|603[2n]bp

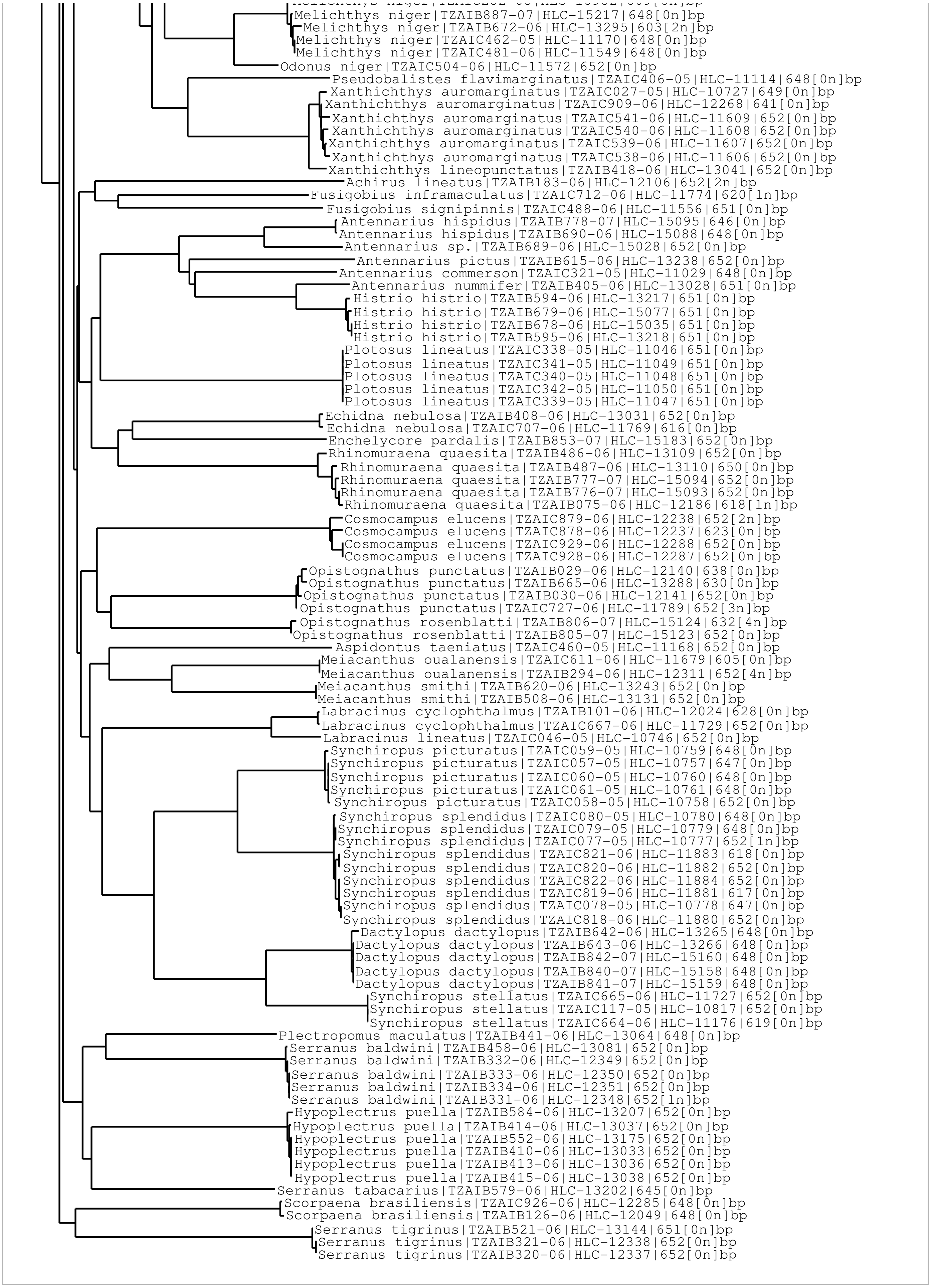
Melichthys niger|TZAIB887-07|HLC-15217|648[0n]bp
Melichthys niger|TZAIB672-06|HLC-13295|603[2n]bp
Melichthys niger|TZAIC462-05|HLC-11170|648[0n]bp
Melichthys niger|TZAIC481-06|HLC-11549|648[0n]bp
Odonus niger|TZAIC504-06|HLC-11572|652[0n]bp
Pseudobalistes flavimarginatus|TZAIC406-05|HLC-11114|648[0n]bp
Xanthichthys auromarginatus|TZAIC027-05|HLC-10727|649[0n]bp
Xanthichthys auromarginatus|TZAIC909-06|HLC-12268|641[0n]bp
Xanthichthys auromarginatus|TZAIC541-06|HLC-11609|652[0n]bp
Xanthichthys auromarginatus|TZAIC540-06|HLC-11608|652[0n]bp
Xanthichthys auromarginatus|TZAIC539-06|HLC-11607|652[0n]bp
Xanthichthys auromarginatus|TZAIC538-06|HLC-11606|652[0n]bp
Xanthichthys lineopunctatus|TZAIB418-06|HLC-13041|652[0n]bp
Achirus lineatus|TZAIB183-06|HLC-12106|652[2n]bp
Fusigobius inframaculatus|TZAIC712-06|HLC-11774|620[1n]bp
Fusigobius signipinnis|TZAIC488-06|HLC-11556|651[0n]bp
Antennarius hispidus|TZAIB778-07|HLC-15095|646[0n]bp
Antennarius hispidus|TZAIB690-06|HLC-15088|648[0n]bp
Antennarius sp.|TZAIB689-06|HLC-15028|652[0n]bp
Antennarius pictus|TZAIB615-06|HLC-13238|652[0n]bp
Antennarius commerson|TZAIC321-05|HLC-11029|648[0n]bp
Antennarius nummifer|TZAIB405-06|HLC-13028|651[0n]bp
Histrio histrio|TZAIB594-06|HLC-13217|651[0n]bp
Histrio histrio|TZAIB679-06|HLC-15077|651[0n]bp
Histrio histrio|TZAIB678-06|HLC-15035|651[0n]bp
Histrio histrio|TZAIB595-06|HLC-13218|651[0n]bp
Plotosus lineatus|TZAIC338-05|HLC-11046|651[0n]bp
Plotosus lineatus|TZAIC341-05|HLC-11049|651[0n]bp
Plotosus lineatus|TZAIC340-05|HLC-11048|651[0n]bp
Plotosus lineatus|TZAIC342-05|HLC-11050|651[0n]bp
Plotosus lineatus|TZAIC339-05|HLC-11047|651[0n]bp
Echidna nebulosa|TZAIB408-06|HLC-13031|652[0n]bp
Echidna nebulosa|TZAIC707-06|HLC-11769|616[0n]bp
Enchelycore pardalis|TZAIB853-07|HLC-15183|652[0n]bp
Rhinomuraena quaesita|TZAIB486-06|HLC-13109|652[0n]bp
Rhinomuraena quaesita|TZAIB487-06|HLC-13110|650[0n]bp
Rhinomuraena quaesita|TZAIB777-07|HLC-15094|652[0n]bp
Rhinomuraena quaesita|TZAIB776-07|HLC-15093|652[0n]bp
Rhinomuraena quaesita|TZAIB075-06|HLC-12186|618[1n]bp
Cosmocampus elucens|TZAIC879-06|HLC-12238|652[2n]bp
Cosmocampus elucens|TZAIC878-06|HLC-12237|623[0n]bp
Cosmocampus elucens|TZAIC929-06|HLC-12288|652[0n]bp
Cosmocampus elucens|TZAIC928-06|HLC-12287|652[0n]bp
Opistognathus punctatus|TZAIB029-06|HLC-12140|638[0n]bp
Opistognathus punctatus|TZAIB665-06|HLC-13288|630[0n]bp
Opistognathus punctatus|TZAIB030-06|HLC-12141|652[0n]bp
Opistognathus punctatus|TZAIC727-06|HLC-11789|652[3n]bp
Opistognathus rosenblatti|TZAIB806-07|HLC-15124|632[4n]bp
Opistognathus rosenblatti|TZAIB805-07|HLC-15123|652[0n]bp
Aspidontus taeniatus|TZAIC460-05|HLC-11168|652[0n]bp
Meiacanthus oualanensis|TZAIC611-06|HLC-11679|605[0n]bp
Meiacanthus oualanensis|TZAIB294-06|HLC-12311|652[4n]bp
Meiacanthus smithi|TZAIB620-06|HLC-13243|652[0n]bp
Meiacanthus smithi|TZAIB508-06|HLC-13131|652[0n]bp
Labracinus cyclophthalmus|TZAIB101-06|HLC-12024|628[0n]bp
Labracinus cyclophthalmus|TZAIC667-06|HLC-11729|652[0n]bp
Labracinus lineatus|TZAIC046-05|HLC-10746|652[0n]bp
Synchiropus picturatus|TZAIC059-05|HLC-10759|648[0n]bp
Synchiropus picturatus|TZAIC057-05|HLC-10757|647[0n]bp
Synchiropus picturatus|TZAIC060-05|HLC-10760|648[0n]bp
Synchiropus picturatus|TZAIC061-05|HLC-10761|648[0n]bp
Synchiropus picturatus|TZAIC058-05|HLC-10758|652[0n]bp
Synchiropus splendidus|TZAIC080-05|HLC-10780|648[0n]bp
Synchiropus splendidus|TZAIC079-05|HLC-10779|648[0n]bp
Synchiropus splendidus|TZAIC077-05|HLC-10777|652[1n]bp
Synchiropus splendidus|TZAIC821-06|HLC-11883|618[0n]bp
Synchiropus splendidus|TZAIC820-06|HLC-11882|652[0n]bp
Synchiropus splendidus|TZAIC822-06|HLC-11884|652[0n]bp
Synchiropus splendidus|TZAIC819-06|HLC-11881|617[0n]bp
Synchiropus splendidus|TZAIC078-05|HLC-10778|647[0n]bp
Synchiropus splendidus|TZAIC818-06|HLC-11880|652[0n]bp
Dactylopus dactylopus|TZAIB642-06|HLC-13265|648[0n]bp
Dactylopus dactylopus|TZAIB643-06|HLC-13266|648[0n]bp
Dactylopus dactylopus|TZAIB842-07|HLC-15160|648[0n]bp
Dactylopus dactylopus|TZAIB840-07|HLC-15158|648[0n]bp
Dactylopus dactylopus|TZAIB841-07|HLC-15159|648[0n]bp
Synchiropus stellatus|TZAIC665-06|HLC-11727|652[0n]bp
Synchiropus stellatus|TZAIC117-05|HLC-10817|652[0n]bp
Synchiropus stellatus|TZAIC664-06|HLC-11176|619[0n]bp
Plectropomus maculatus|TZAIB441-06|HLC-13064|648[0n]bp
Serranus baldwini|TZAIB458-06|HLC-13081|652[0n]bp
Serranus baldwini|TZAIB332-06|HLC-12349|652[0n]bp
Serranus baldwini|TZAIB333-06|HLC-12350|652[0n]bp
Serranus baldwini|TZAIB334-06|HLC-12351|652[0n]bp
Serranus baldwini|TZAIB331-06|HLC-12348|652[1n]bp
Hypoplectrus puella|TZAIB584-06|HLC-13207|652[0n]bp
Hypoplectrus puella|TZAIB414-06|HLC-13037|652[0n]bp
Hypoplectrus puella|TZAIB552-06|HLC-13175|652[0n]bp
Hypoplectrus puella|TZAIB410-06|HLC-13033|652[0n]bp
Hypoplectrus puella|TZAIB413-06|HLC-13036|652[0n]bp
Hypoplectrus puella|TZAIB415-06|HLC-13038|652[0n]bp
Serranus tabacarius|TZAIB579-06|HLC-13202|645[0n]bp
Scorpaena brasiliensis|TZAIC926-06|HLC-12285|648[0n]bp
Scorpaena brasiliensis|TZAIB126-06|HLC-12049|648[0n]bp
Serranus tigrinus|TZAIB521-06|HLC-13144|651[0n]bp
Serranus tigrinus|TZAIB321-06|HLC-12338|652[0n]bp
Serranus tigrinus|TZAIB320-06|HLC-12337|652[0n]bp
